# Nickel-Catalyzed Suzuki Coupling of Phenols Enabled by SuFEx of Tosyl Fluoride

**DOI:** 10.3390/molecules28020636

**Published:** 2023-01-07

**Authors:** Huimin Wang, Shuqin Zhang, Minling Xü, Gang Zou

**Affiliations:** School of Chemistry and Molecular Engineering, East China University of Science and Technology, 130 Meilong Rd, Shanghai 200237, China

**Keywords:** Suzuki coupling, SuFEx, phenols, arylboronic acids, nickel catalysis, tosyl fluoride

## Abstract

A practical and efficient Suzuki coupling of phenols has been developed by using *trans*-NiCl(*o*-Tol)(PCy_3_)_2_/2PCy_3_ as a catalyst in the presence of tosyl fluoride as an activator. The key for the direct use of phenols lies in the compatibility of the nickel catalyst with tosyl fluoride (TsF) and its sulfur(VI) fluoride exchange (SuFEx) with C_Ar_-OH. Water has been found to improve the one-pot process remarkably. The steric and electronic effects and the functional group compatibility of the one-pot Suzuki coupling of phenols appear to be comparable to the conventional one of pre-prepared aryl tosylates. A series of electronically and sterically various biaryls could be obtained in good to excellent yields by using 3–10 mol% loading of the nickel catalyst. The applications of this one-pot procedure in chemoselective derivatization of complex molecules have been demonstrated in 3-phenylation of estradiol and estrone.

## 1. Introduction

Construction of biaryls has attracted the most interest in applications of Suzuki coupling because of their ubiquitousness in important fine chemicals, e.g., pharmaceuticals [1,2], advanced organic materials [3], pesticides, etc. [4]. Since the seminal report from Percec using aryl tosylates in Suzuki coupling [5], many efforts have been devoted to using O-based pseudohalides, e.g., triflates [6,7,8,9,10,11,12,13,14], sulfonates [15], carbonate [16], sulfamates [16], carbamates [16,17,18], etc., as an alternative to aryl halides because of the good diversity, abundance, and availability of phenols [19]. Obviously, from the practical point of view, it is desirable to in situ form these phenol derivatives in the very Suzuki coupling system, instead of pre-preparing them in a separate step. In fact, several reports had described direct use of phenols in palladium or nickel-catalyzed Suzuki coupling through in situ formation of tosylates [20,21], nonaflates [22], pivalates [23], heteroaryl ethers [24], and even phenolic salts [25,26]. Comparing with palladium, nickel-based catalysts are not only less expensive but also have shown higher activities in Suzuki coupling of O-based pseudohalides [27,28,29,30,31,32,33,34,35]. However, the rich redox chemistry of nickel often makes nickel-based catalysts less compatible with the conditions and reagents required for in situ derivatization of C_Ar_-OH in one-pot processes. Therefore, it is important to further develop C_Ar_-OH activation for one-pot nickel-catalyzed Suzuki coupling of phenols to practically construct biaryls.

Sulfur(VI) fluoride exchange (SuFEx), the so-called second generation of click reaction [36,37,38,39], has proven to be a privileged protocol in activating phenolic C_Ar_-OH group via chemoselective formation of sulfonates with SO_2_F-containing reagents in the presence of various other nucleophiles [37,40]. As a part of our continuous efforts to develop practical procedures for construction of biaryls from pseudohalides [41,42,43,44], we report herein a SuFEx enabled, nickel/phosphine catalyzed Suzuki coupling of phenols via in situ formation of tosylates with tosyl fluoride (TsF) using *trans*-NiCl(*o*-Tol)(PCy_3_)_2_/2PCy_3_ as a catalyst to practically and efficiently synthesize various biaryls with good functional group comparability.

## 2. Results and Discussion

The cheap, readily available and easy-to-handle tosyl fluoride (TsF) has been chosen as the sulfur(VI) fluoride in SuFEx for practical purposes. Using the reaction of 4-hydroxyacetophenone (**1a**) with phenylboronic acid (**2a**) as a model, although the common nickel dichloride/phosphine complexes NiCl_2_(PR_3_)_2_Cl_2_ (R = PPh_3_, PCy_3_, dppf) were almost inactive, an aryl nickel/phosphine catalyst, *trans*-NiCl(Ph)(PPh_3_)_2_ (**cat-1**), which had been established for efficient Suzuki coupling of tosylates [42,45], offered a promising result, giving the desired product **3aa** in 26% yield with 5 mol% catalyst loading in the presence of K_3_PO_4_·3H_2_O in aqueous THF (THF/H_2_O = 4/1, vol/vol) (Table 1). Encouraged by the preliminary result of *trans*-NiCl(Ph)(PPh_3_)_2_, structural effects of the nickel complexes with respect to aryl and phosphine ligands were briefly explored to increase catalytic efficiency. A delicate balance between the steric hindrance of the aryl group and catalytic activity of the nickel catalysts was observed. For example, the phenyl groups bearing a small ortho alkyl substituent, i.e., *trans*-NiCl(*o*-Tol)(PPh_3_)_2_ (**cat-2**, 59%) and *trans*-NiCl(*o*-EtPh)(PPh_3_)_2_ (**cat-3**, 56%), as well as anthracen-9-yl (**cat-6**, 46%) could increase **3aa** yields while naphthalen-1-yl (**cat-7**, 30%) and more sterically demanding aryls, e.g., 2-isopropylphenyl (**cat-4**, 20%) and 2,6-dimethylphenyl (**cat-5**, 17%), gave no improvement or even poorer results (Table 1, entries 2–8). The supporting phosphine ligand also played an important role in the catalytic activity of the aryl nickel complexes. The product (**3aa**) yields increased to 55% and 87% using tricyclohexylphosphine complexes of *trans*-NiCl(Ph)(PCy_3_)_2_ (**cat-8**) and *trans*-NiCl(*o*-Tol)(PCy_3_)_2_ (**cat-9**) from 26% and 59% with the triphenyl phosphine analogs, **cat-1** and **cat-2**, respectively (Table 1, entries 2, 3, 9 and 10). In fact, the yield was further increased to 96% by using extra 10 mol% tricyclohexylphosphine ligand with 5 mol% *trans*-NiCl(*o*-Tol)(PCy_3_)_2_ (**cat-9**). A satisfactory yield (93%) could still be obtained by using 3 mol% *trans*-NiCl(*o*-Tol)(PCy_3_)_2_/2PCy_3_ although the reaction became slow and required 10 h to complete. Use of 1 mol% catalyst loading led to incomplete reaction within 12 h and a significantly lower yield (32%) for **3aa** (Table 1, entries 11–13).

Water proved to be crucial for high catalytic efficiency. The desired product **3aa** was isolated in a lower yield (83%) in pure THF solution using hydrous base, 5 equiv. K_3_PO_4_·3H_2_O, while only a 12% yield could be isolated in strict anhydrous conditions (Table 1, entries 14 and 15). Increasing the volume ratio of water to THF from 1/4 to 1/2 significantly decreased **3aa** yield to 78%, possibly due to the limited solubility of the tosylate intermediate in water. Bases and organic co-solvents screening failed to improve the reaction comparing with the K_3_PO_4_·3H_2_O in THF/H_2_O system. A couple of common solvents, e.g., DMF, DMSO, toluene, DME, etc., gave lower yields while CH_3_CN and dioxane performed similarly to THF (Table 1, entries 19–25). K_3_PO_4_·3H_2_O appeared to be the choice of base. In fact, when weaker or stronger bases, e.g., K_2_CO_3_, KOAc, NaOH and KOH, were used to replace K_3_PO_4_·3H_2_O, much lower yields were obtained (Table 1, entries 26–29). No reaction was observed using NaF and KF as bases, excluding any positive role played by the SuFEx by-product fluoride in the system. Therefore, the optimal conditions for the SuFEx enabled Suzuki coupling of phenols were set as 3 mol% *trans*-NiCl(*o*-Tol)(PCy_3_)_2_/2PCy_3_ in THF/H_2_O (4/1, vol/vol) using 5 equiv. K_3_PO_4_·3H_2_O as base (Table 1, entry 12). A control experiment using 1.1 equiv. TsCl instead of TsF under the otherwise identical conditions confirmed the necessary role of SuFEx in the one-pot procedure. The nickel catalyst appeared to be completely deactivated by TsCl since no coupling product was detected while formation of tosylate intermediate could still take place. Indeed, when a second dose of the catalyst, 3 mol% *trans*-NiCl(*o*-Tol)(PCy_3_)_2_/2PCy_3_, was added to the very reaction mixture after TsCl was consumed, **3aa** could be isolated in 57% yield after 6 h reaction.

With the optimal conditions in hand, the scope of the SuFEx enabled, nickel-catalyzed Suzuki coupling of phenols with arylboronic acids was briefly explored (Table 2). Given the fact that there are two sequential steps, i.e., tosylation via SuFEx and the following nickel-catalyzed cross-coupling, for the phenol counterpart in the one-pot procedure, the structural effects of phenols were investigated at first. Phenols bearing 4-CO_2_Et (**1b**, 96%) or 4-CN (**1c**, 95%) group at *para*-position reacted similarly to **1a** (4-Ac) affording the products 4-ethoxycarbonylbiphenyl (**3ba**, 96%) and 4-cyanobiphenyl (**3ca**, 95%) in excellent yields, respectively, while the 4-NO_2_ (**1d**) substituted one gave a low yield (45%).

Similar to the report in literature [46], 4-biphenyl aldehyde (**3ea**) appeared to be labile to air-oxidation to acid and was isolated in only 50% yield along with carboxylic acid in 42% yield. A modest yield (40%) was obtained for 4-chlorobiphenyl (**3ga**) because of the intramolecular competition between C_Ar_-Cl and the 4-tosyloxy groups in **1g** [47,48,49], consistent with the comparable reactivities of aryl tosylates to chlorides in nickel-catalyzed Suzuki coupling. In fact, the by-products 4-tosyloxybiphenyl and 4-phenylbiphenyl were also obtained in 35% and 12% yields, respectively. Phenols with electron-rich and electron-neutral substituents, such as 4-CH(CH_3_)_2_ (**1h**) and 4-OMe (**1i**), displayed low reactivities and required 10 mol% catalyst loading to reach good yields (**3ha**, 90% and **3ia**, 90%). The amino group (4-NH_2_, **3ja**, 18%) obviously disturbed the tosylation of phenolic OH group, while the C_Ar_-OH group could be preferentially coupled in the presence of an aliphatic hydroxyl group (**1l**), and the desired product **3la** could be obtained in 74% yield. *ortho*-Substituted phenols gave lower yields than *para*-substituted analogs e.g., **3ma** (*o*-Ac, 70%), **3na** (*o*-CHO, 38%), **3pa** (*o*-^t^Bu, 16%), **3qa** (*o*-OMe, 30%) and **3ra** (*o*-OMe, 40%), although the yields **3pa** (*o*-^t^Bu, 43%), **3qa** (*o*-OMe, 65%), and **3ra** (*o*-OMe, 86%) could increase with 10 mol% catalyst loading, indicating a large steric effect from phenol substrates. Furthermore, when catechol **1s** was investigated, biphenyl-2-yl tosylate (**3sa**, 93%) was isolated as a sole cross-coupling product even with 2.5 equiv. TsF and phenylboronic acid (**2a**), indicating that it is the cross-coupling step, instead of tosylation via SuFEx, should be sensitive to the steric hindrance. In contrast, 4-phenylbiphenyl (**3ta**) could be obtained in 94% yield from hydroquinone (**1t**) under the otherwise identical conditions for catechol. 3-Phenylation of estradiol (**1u**) and estrone (**1v**) clearly demonstrated the feasibility of this one-pot Suzuki coupling of phenols in chemoselective derivatization of complex molecules.

Unlike the large structural effects of phenol counterpart, electronically and sterically various aryl boronic acids could be cross-coupled smoothly to provide biaryls in good to excellent yields. Aryl boronic acids bearing an electron-donating group, e.g., 4-Me (**2b**, 95%) or 4-OMe (**2d**, 90%) appeared to be some more reactive than their analogs with an electron-withdrawing group, e.g., 4-CF_3_ (**2f**, 74%) or 4-CHO (**2g**, 80%). Steric hindrance from aryl boronic acids could be also tolerated to a large extent. For example, 2-Et (**2h**), 2-CH(CH_3_)_2_ (**2i**), 2-Ph (**2j**) and 2-CHO (**2k**) phenyl boronic acids reacted with **1a** to give **3ah**, **3ai, 3aj,** and **3ak** in 91%, 87%, 72%, and 68% yields, respectively. 2-Thiophenyl (**2l**) and 3-furanyl boronic acids (**2m**) could also react to give the products (**3al**, 79%) and (**3am**, 75%), although 3-pyridyl boronic acid failed, possibly, at least in part, due to its poor solubility.

**Scheme 1:**
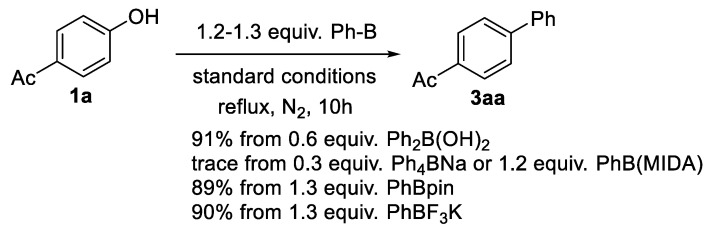
Cross-coupling of **1a** with alternative phenyl boron compounds.

Given the small structural effects from aryl boronic acid counterpart, we anticipated that the other aryl boron reagents, e.g., the shelf-stable and easy-to-handle derivatives of aryl boronic acids, potassium trifluoroboronates, N-methyliminodiacetic acid (MIDA) boronates or boronic acid pinacol esters, and the cost-effective diarylborinic acids, should be also usable in the one-pot procedure. In fact, excellent yields could be obtained for **3aa** with potassium phenyltrifluoroboronate (PhBF_3_K, 1.3 equiv.), phenylboronic acid pinacol ester (PhB(pin), 1.3 equiv.), and diphenyl borinic acid (Ph_2_B(OH), 0.6 equiv.), although MIDA phenylboronate and sodium tetraphenylboronate failed (Scheme 1).

**Scheme 2:**
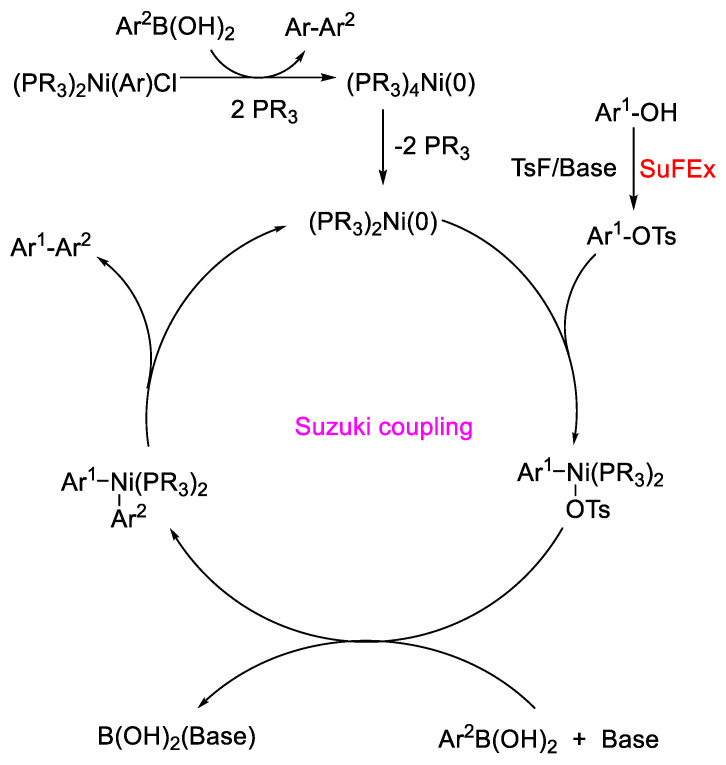
A plausible catalytic cycle for the nickel-catalyzed Suzuki coupling of phenols enabled by SuFEx of tosyl fluoride.

A plausible mechanism has been proposed for the nickel-catalyzed Suzuki coupling of phenols enabled by SuFEx of tosyl fluoride (Scheme 2). Given the observation of the homocoupling product of aryl boronic acids, the aryl nickel(II) di(phosphine) complexes are most likely reduced to nickel(0) phosphine complexes, which require 2 equiv. more phosphine ligand to be stable, by aryl boronic acids via sequential transmetalation and reductive elimination. The facts that sharply different structural effects of phenol vs. boron counterparts, as well as the isolation of biphenyl-2-yl tosylate as a sole product from catechol, imply that the oxidative addition of nickel to tosylate intermediates, instead of the SuFEx or aryl transmetalation from boron, should be the rate-determining step in the catalytic cycle. The better performance of electron-richer PCy_3_ than PPh_3_ and the requirement of extra 2 equiv. PCy_3_ with respect to *trans*-NiCl(*o*-Tol)(PCy_3_)_2_ support the speculation of rate-determining oxidative addition of tosylate intermediate to the nickel(0) species.

## 3. Materials and Methods

### 3.1. General Information

All reactions were carried out under nitrogen by using standard Schlenk techniques unless otherwise stated. Commercially available chemicals were used as received without further purification. Nickel complexes, **Cat 1**–**Cat 9**, were prepared according to previously reported procedures [45,50,51]. The reaction progress was monitored by TLC. Column chromatograph was performed on 300–400 mesh silica gel. ^1^H and ^13^C NMR spectra were recorded in CDCl_3_ or Acetone-d_6_ at ambient temperature on a Bruker DPX-400 spectrometer (Bruker BioSpin GmbH, Germany). Chemical shifts (δ) in NMR are reported in ppm, relative to the internal standard of tetramethylsilane (TMS) or residues of the deuterated solvents. Coupling constants J are reported in Hz. Proton coupling patterns were described as singlet (s), doublet (d), triplet (t), quartet (q), and multiple (m). High-resolution mass spectra (HRMS) were measured with an Agilent mass spectrometer (HR-TOF-MS, EI).

### 3.2. General Procedure for Nickel/Phosphine Catalyzed Suzuki Coupling of Phenols with Aryl Boronic Acids Enabled by SuFEx of TsF

To a 25 mL dry flask were added phenol **1** (1.0 mmol), aryl boronic acid **2** (1.3 mmol), TsF (1.1 mmol, 0.193g), *trans*-NiCl(*o*-Tol)(PCy_3_)_2_ (3 mol%, 0.022g), PCy_3_ (6 mol%, 0.017g), and K_3_PO_4_·3H_2_O (5.0 mmol, 1.332g). The air in the flask was replaced by N_2_ using standard Schlenk techniques before solvents THF (4.0 mL) and H_2_O (1.0 mL) were added by a syringe. The mixture was stirred under N_2_ atmosphere at 70 °C (bath temperature) for a given time or monitored by TLC until the reaction completed. The reaction mixture was diluted with CH_2_Cl_2_ (15 mL), followed by washing with H_2_O (3 × 10 mL). The organic layer was separated, dried over anhydrous Na_2_SO_4_, filtered, and evaporated under reduced pressure to give a crude product, which was purified by column chromatography on silica gel to afford biaryl compound **3**.

All known products were characterized by comparing their NMR with those reported in literature and the new compound, 2-tosyloxybiphenyl (**3sa**), was further characterized by HRMS. For details, see Supplementary Materials.

## 4. Conclusions

In summary, a practical Suzuki coupling of phenols enabled by SuFEx of tosyl fluoride has been developed by using *trans*-NiCl(*o*-Tol)(PCy_3_)_2_/2PCy_3_ as catalyst in the presence of 5 equiv. K_3_PO_4_·3H_2_O in aqueous THF. Both aryl and phosphine ligands in the aryl nickel complexes have proven to affect their catalytic performance significantly. Water in the system has also been found to play an important role for high catalytic efficiency. Large structural effects from phenols have been observed while the electronic and steric influences from the boron counterpart appeared to be comparably small, if not negligible. A series of electronically and sterically various biaryls could be obtained in good to excellent yields by using the SuFEx enabled, nickel-catalyzed one-pot Suzuki coupling, eliminating the pre-preparation of tosylates. The potentials to apply the practical one-pot procedure in derivatization of complex molecules have also been demonstrated.

## Figures and Tables

**Table 1:** Optimization of the SuFEx-enabled, Ni-catalyzed Suzuki coupling of phenols ^a^.

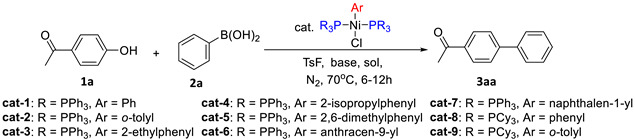					
Entry	Cat. (mol%)	Ligand	Base (equiv.)	Sol. (vol/vol)	Yield(%) ^b^
1	NiCl_2_(PR_3_)_2_ (5) ^c^	/	K_3_PO_4_·3H_2_O (5)	THF/H_2_O (4/1)	trace
2	**cat-1** (5)	/	K_3_PO_4_·3H_2_O (5)	THF/H_2_O (4/1)	26
3	**cat-2** (5)	/	K_3_PO_4_·3H_2_O (5)	THF/H_2_O (4/1)	59
4	**cat-3** (5)	/	K_3_PO_4_·3H_2_O (5)	THF/H_2_O (4/1)	56
5	**cat-4** (5)	/	K_3_PO_4_·3H_2_O (5)	THF/H_2_O (4/1)	20
6	**cat-5** (5)	/	K_3_PO_4_·3H_2_O (5)	THF/H_2_O (4/1)	17
7	**cat-6** (5)	/	K_3_PO_4_·3H_2_O (5)	THF/H_2_O (4/1)	46
8	**cat-7** (5)	/	K_3_PO_4_·3H_2_O (5)	THF/H_2_O (4/1)	30
9	**cat-8** (5)	/	K_3_PO_4_·3H_2_O (5)	THF/H_2_O (4/1)	55
10	**cat-9** (5)	/	K_3_PO_4_·3H_2_O (5)	THF/H_2_O (4/1)	87
11	**cat-9** (5)	PCy_3_(10)	K_3_PO_4_·3H_2_O (5)	THF/H_2_O (4/1)	96
12	**cat-9** (3)	PCy_3_(6)	K_3_PO_4_·3H_2_O (5)	THF/H_2_O (4/1)	93 ^d^
13	**cat-9** (1)	PCy_3_(2)	K_3_PO_4_·3H_2_O (5)	THF/H_2_O (4/1)	32 ^d^
14	**cat-9** (3)	PCy_3_(6)	K_3_PO_4_·3H_2_O (5)	THF	83
15	**cat-9** (3)	PCy_3_(6)	K_3_PO_4_ (5)	THF	12
16	**cat-9** (3)	PCy_3_(6)	K_3_PO_4_·3H_2_O (5)	THF/H_2_O (2/1)	78 ^d^
17	**cat-9** (3)	PCy_3_(6)	K_3_PO_4_·3H_2_O (5)	THF/H_2_O (6/1)	93 ^d^
18	**cat-9** (3)	PCy_3_(6)	K_3_PO_4_·3H_2_O (5)	THF/H_2_O (8/1)	90 ^d^
19	**cat-9** (3)	PCy_3_(6)	K_3_PO_4_·3H_2_O (5)	Diox/H_2_O (4/1)	87
20	**cat-9** (3)	PCy_3_(6)	K_3_PO_4_·3H_2_O (5)	DME/H_2_O (4/1)	15
21	**cat-9** (3)	PCy_3_(6)	K_3_PO_4_·3H_2_O (5)	Tol/H_2_O (4/1)	78
22	**cat-9** (3)	PCy_3_(6)	K_3_PO_4_·3H_2_O (5)	MeCN/H_2_O(4/1)	90
23	**cat-9** (3)	PCy_3_(6)	K_3_PO_4_·3H_2_O (5)	DMF/H_2_O (4/1)	66
24	**cat-9** (3)	PCy_3_(6)	K_3_PO_4_·3H_2_O (5)	DMA/H_2_O (4/1)	77
25	**cat-9** (3)	PCy_3_(6)	K_3_PO_4_·3H_2_O (5)	DMSO/H_2_O(4/1)	75
26	**cat-9** (3)	PCy_3_(6)	K_2_CO_3_ (5)	THF/H_2_O (4/1)	20
27	**cat-9** (3)	PCy_3_(6)	NaOH (5)	THF/H_2_O (4/1)	18
28	**cat-9** (3)	PCy_3_(6)	AcOK (5)	THF/H_2_O (4/1)	47
29	**cat-9** (3)	PCy_3_(6)	KOH (5)	THF/H_2_O (4/1)	30
30	**cat-9** (3)	PCy_3_(6)	NaF/KF (5)	THF/H_2_O (4/1)	trace
31	**cat-9** (3)	PCy_3_(6)	K_3_PO_4_·3H_2_O (4)	THF/H_2_O (4/1)	82

^a^ Reaction conditions: **1a** (1.0 mmol), **2a** (1.3 mmol), TsF (1.1 mmol), solvent (5.0 mL), N_2_, at reflux or 70 °C, 6 h. ^b^ Isolated yields. ^c^ PR_3_ = PPh_3_, PCy_3_ and dppf (1,1′- bis(diphenylphosphino)ferrocene). ^d^ 10–12 h.

**Table 2:** Nickel/phosphine catalyzed Suzuki coupling of phenols with arylboronic acids enabled by SuFEx of TsF ^a^.

Nickel/Phosphine Catalyzed Suzuki Coupling of Phenols with Arylboronic Acids Enabled by SuFEx of TsF
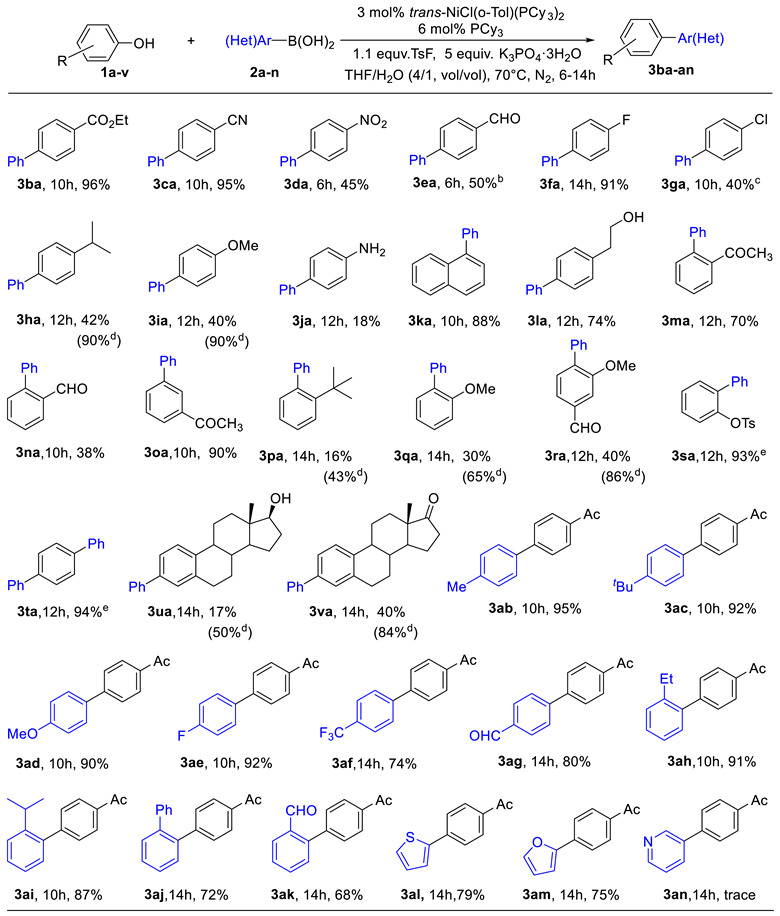

^a^ Reaction conditions: **1a-v** (1.0 mmol), **2a-n** (1.3 mmol), TsF (1.1 mmol), *trans*-NiCl(*o*-Tol)(PCy_3_)_2_ (3.0 mol%), PCy_3_ (6.0 mol%), K_3_PO_4_·3H_2_O (5.0 mmol), THF (4.0 mL), H_2_O (1.0 mL), N_2_, 70 °C. ^b^ 4-Biphenylcarboxylic acid isolated in 42%. ^c^ 4-Tosyloxybiphenyl (35%) and 4-phenylbiphenyl (12%) isolated. ^d^ *trans*-NiCl(*o*-Tol)(PCy_3_)_2_ (10.0 mol%), PCy_3_ (20.0 mol%) used. ^e^ 2.5 equiv. TsF and **2a** used with respect to **1s** and **1t**.

## Data Availability

Data are in the Supplementary Materials.

## Supplementary Materials

The following supporting information can be downloaded at: https://www.mdpi.com/article/10.3390/molecules28020636/s1, Characterization data and copies of ^1^H & ^13^C NMR spectra of products (References [21,42,43,52,53,54,55,56,57,58,59,60,61,62] are cited in the Supplementary Materials).

## Sample Availability

Samples of the products are available from the authors.
