# Supplementary material for: Nickel-Catalyzed Suzuki Coupling of Phenols Enabled by SuFEx of Tosyl Fluoride
Source: Molecules. 2023 Jan 7;28(2):636. doi: 10.3390/molecules28020636 (PMC9864267; doi:10.3390/molecules28020636)

Supplementary Materials
For

Nickel-Catalyzed Suzuki Coupling of Phenols Enabled by SuFEx of Tosyl Fluoride

Huimin Wang, Shuqin Zhang, Minling Xü, Gang Zou*

School of Chemistry and Molecular Engineering, East China University of Science and Technology, 130 Meilong Rd, Shanghai, China

Table of Contents

General information......2
Characterization data of compounds......2
References......12
^{1}H & ^{13}C NMR Spectra......13

General information

^{1}H and ^{13}C NMR spectra were recorded in $CDCl_3$ or Acetone-d_6 at ambient temperature on a Bruker DPX-400 spectrometer (Bruker BioSpin GmbH, Germany). Chemical shifts (δ) in NMR are reported in ppm, relative to the internal standard of tetramethylsilane (TMS) or residues of the deuterated solvents. Coupling constants J are reported in Hz. Proton coupling patterns were described as singlet (s), doublet (d), triplet (t), quartet (q), and multiple (m). High-resolution mass spectra (HRMS) were measured with an Agilent mass spectrometer (HR-TOF-MS, EI).

Characterization data of compounds

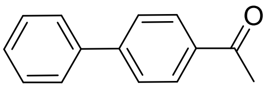

3aa

4-Acetylbiphenyl (3aa)[1] (0.182g, 93% isolated yield as white solid), M.P. 120-122 °C. ^{1}H NMR (400 MHz, $CDCl_3$) δ (ppm): 8.02 (d, J = 8.4 Hz, 2H), 7.69 (d, J = 8.4 Hz, 2H), 7.63 (d, J = 7.2 Hz, 2H), 7.48 (t, J = 7.2 Hz, 2H), 7.42 - 7.39 (m, 1H), 2.64 (s, 3H). ^{13}C NMR (100 MHz, $CDCl_3$) δ (ppm): 197.8, 145.8, 139.9, 135.8, 129.0, 129.0, 128.3, 127.3, 127.2, 26.7.

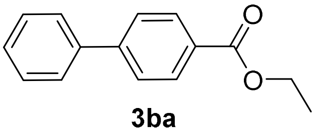

3ba

4-Ethoxycarbonylbiphenyl (3ba)[2] (0.217g, 96% isolated yield as white solid), M.P. 92-94 °C. ^{1}H NMR (400 MHz, $CDCl_3$) δ (ppm): 8.13 (d, J = 8.4 Hz, 2H), 7.62 - 7.68 (m, 4H), 7.47 (t, J = 7.2 Hz, 2H), 7.40 (t, J = 7.2 Hz, 1H), 4.41 (q, J =7.2 Hz, 2H), 1.43 (t, J =7.2 Hz, 3H). ^{13}C NMR (100 MHz, $CDCl_3$) δ (ppm): 166.6, 145.6, 140.1, 130.1, 129.2, 129.0, 128.1, 127.3, 127.0, 61.0, 14.4.

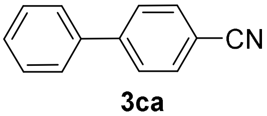

3ca

4-Cyanobiphenyl (3ca)[3] (0.170g, 95% isolated yield as white solid), M.P. 86-89 °C. ^{1}H NMR (400 MHz, $CDCl_3$) δ (ppm): 7.73 - 7.67 (m, 4H), 7.61 - 7.58 (m, 2H), 7.51 - 7.41 (m, 3H). ^{13}C NMR (100 MHz, $CDCl_3$) δ (ppm): 145.6, 139.1, 132.6, 129.1, 128.7, 127.7, 127.2, 119.0, 110.9.

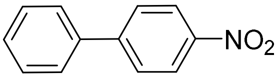

3da

4-Nitrobiphenyl (3da)[1] (0.089g, 45% isolated yield as yellow solid), M.P. 113-114 ºC. ^{1}H NMR (400 MHz, $CDCl_3$) δ (ppm): 8.30 (d, *J* = 8.8 Hz, 2H), 7.74 (d, *J* = 8.8 H, 2Hz), 7.63 (d, *J* = 7.2 Hz, 2H), 7.52 - 7.43 (m, 3H). ^{13}C NMR (100 MHz, $CDCl_3$) δ (ppm): 147.7, 147.1, 138.8, 129.2, 129.0, 127.8, 127.4, 124.1.

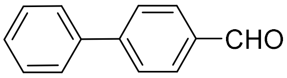

3ea

4-Aldehydebiphenyl (3ea)[2] (0.091g, 50% isolated yield as yellow solid), M.P. 57-58 ºC. ^{1}H NMR (400 MHz, D_6-Acetone) δ (ppm): 10.09 (s, 1H), 8.01 (d, *J* = 8.4 Hz, 2H), 7.88 (d, *J* = 8.4 Hz, 2H), 7.75 (d, *J* = 7.2 Hz, 2H), 7.51 (t, *J* =7.2 Hz, 2H), 7.44 (t, *J* = 7.2 Hz, 1H). ^{13}C NMR (100 MHz, D_6-Acetone) δ (ppm): 192.6, 147.6, 140.5, 136.6, 131.0, 130.0, 129.4, 128.5, 128.2.

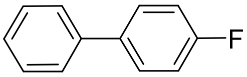

3fa

4-Fluorobiphenyl (3fa)[4] (0.157g, 91% isolated yield as white solid), M.P. 71-73 °C. ^{1}H NMR (400 MHz, $CDCl_3$) *δ* (ppm): 7.59 - 7.54 (m, 4H), 7.46 (t, *J* = 7.2 Hz, 2H), 7.39 - 7.35 (m, 1H), 7.17 - 7.13 (m, 2H). ^{13}C NMR (100 MHz, $CDCl_3$) *δ* (ppm): 163.7, 161.3, 140.3, 137.4 (d, *J* = 3.3 Hz), 128.9, 128.8, 128.7, 127.2, 127.1, 115.6 (d, *J* = 21.3 Hz).

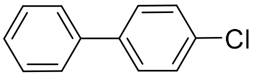

3ga

4-Chlorobiphenyl (3ga)[4] (0.075g, 40% isolated yield as white solid), M.P. 77-79 °C. ^{1}H NMR (400 MHz, $CDCl_3$) *δ* (ppm): 7.58 - 7.53 (m, 4H), 7.48 - 7.36 (m, 5H). ^{13}C NMR (100 MHz, $CDCl_3$) *δ* (ppm): 140.0, 139.7, 133.4, 129.0, 128.9, 128.4, 127.6, 127.0.

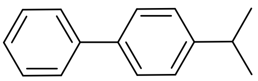

3ha

4-Isopropylbiphenyl (3ha)[5] (0.082g, 42%; 0.176, 90% isolated yield as colourless oil). ^{1}H NMR (400 MHz, CDCl$_3$) δ (ppm): 7.64 (d, J = 7.2 Hz, 2H), 7.58 (d, J = 8.4 Hz, 2H), 7.47 (t, J = 7.6 Hz, 2H), 7.39 - 7.35 (m, 3H), 3.04 - 2.97 (sept, J = 8.0 Hz, 1H), 1.35 (d, J = 6.8 Hz, 6H). ^{13}C NMR (100 MHz, CDCl$_3$) δ (ppm): 148.0, 141.2, 138.8, 128.8, 127.1, 127.1, 127.1, 126.9, 33.9, 24.1.

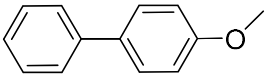

3ia

4-Methoxybiphenyl (3ia)[1] (0.074g, 40%; 0.166g, 90% isolated yield as white solid), M.P. 88-89 ºC. ^{1}H NMR (400 MHz, CDCl$_3$) δ (ppm): 7.60 - 7.56 (m, 4H), 7.45 (t, J = 7.6 Hz, 2H), 7.34 (t, J = 7.6 Hz,1H), 7.01 (d, J = 8.8 Hz, 2H), 3.88 (s, 3H). ^{13}C NMR (100 MHz, CDCl$_3$) δ (ppm): 159.2, 140.8, 133.8, 128.8, 128.2, 126.8, 126.7, 114.2, 55.4.

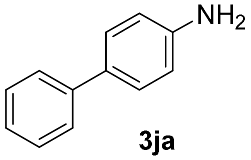

3ja

4-Phenylaniline (3ja)[3] (0.030, 18% isolated yield as brown solid), M.P. 45-47 ºC. ^{1}H NMR (400 MHz, CDCl$_3$) δ (ppm): 7.57 (d, J = 8.4 Hz, 2H), 7.46 - 7.41 (m, 4H), 7.32 - 7.28 (m, 1H), 6.78 (d, J = 8.4 Hz, 2H), 3.72 (br, 2H). ^{13}C NMR (100 MHz, CDCl$_3$) δ (ppm): 145.8, 141.1, 131.4, 128.7, 127.9, 126.4, 126.2, 115.4.

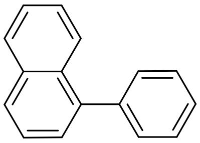

3ka

1-Phenylnaphthalene (3ka)[6] (0.179g, 88% isolated yield as white solid), M.P. 44-46 ºC. ^{1}H NMR (400 MHz, CDCl$_3$) δ (ppm): 8.02 - 7.93 (m, 3H), 7.62 - 7.49 (m, 9H). ^{13}C NMR (100 MHz, CDCl$_3$) δ (ppm): 140.8, 140.3, 133.8, 131.6, 130.1, 128.3, 127.7, 127.3, 127.0, 126.1, 125.8, 125.4.

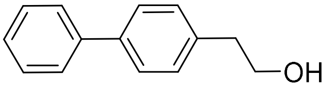

3la

1-Biphenyl-4-ethanol (3la)[7] (0.146g, 74% isolated yield as white solid), M.P. 93-94 °C. ^{1}H NMR (400 MHz, $CDCl_3$) δ (ppm): 7.64 - 7.58 (m, 4H), 7.49 (t, J = 7.6 Hz, 2H), 7.41 - 7.33 (m, 3H), 3.91 (t, J = 6.4 Hz, 2H), 2.94 (t, J = 6.4 Hz, 2H), 2.08 (s, 1H). ^{13}C NMR (100 MHz, $CDCl_3$) δ (ppm): 140.9, 139.4, 137.7, 129.4, 128.8, 127.2, 127.1, 127.0, 63.5, 38.8.

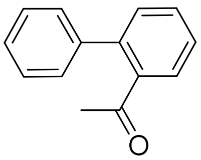

3ma

2-Acetylbiphenyl (3ma)[1] (0.137g, 70% isolated yield as white solid), M.P. 54-57 °C. ^{1}H NMR (400 MHz, $CDCl_3$) δ (ppm): 8.20 (s, 1H), 7.94 (d, J = 7.6 Hz, 1H), 7.79 (d, J = 8.0 Hz, 1H), 7.63 (d, J = 7.6 Hz, 2H), 7.54 (t, J = 8.0 Hz, 1H), 7.48 (t, J = 8.0 Hz, 2H), 7.39 (t, J = 7.2 Hz, 1H), 2.66 (s, 3H). ^{13}C NMR (100 MHz, $CDCl_3$) δ (ppm): 198.2, 141.7, 140.2, 137.6, 131.8, 129.1, 128.9, 127.8, 127.2, 127.0, 26.8.

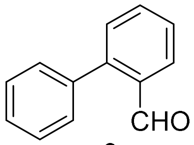

3na

2-Aldehydebiphenyl (3na)[8] (0.069g, 38% isolated yield as colourless oil). ^{1}H NMR (400 MHz, $CDCl_3$) δ (ppm): 9.99 (s, 1H), 8.04 (dd, J = 8.0, 1.2 Hz, 1H), 7.64 (td, J = 7.6, 1.6 Hz, 1H), 7.52 - 7.44 (m, 5H), 7.40 - 7.37 (m, 2H). ^{13}C NMR (100 MHz, $CDCl_3$) δ (ppm): 192.5, 146.0, 137.8, 133.7, 133.6, 130.8, 130.1, 128.5, 128.2, 127.8, 127.6.

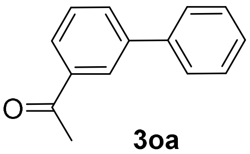

3oa

3-Acetylbiphenyl (3oa)[1] (0.177g, 90% isolated yield as yellow oil). ^{1}H NMR (400 MHz, $CDCl_3$) δ (ppm): 8.19 (t, J = 1.6 Hz, 1H), 7.94 (dd, J = 1.6, 8.0 Hz, 1H), 7.80 (dd, J = 7.6, 1.2 Hz, 1H), 7.62 (d, J = 7.6 Hz, 2H), 7.54 (t, J = 7.6 Hz, 1H), 7.48 (t, J = 8.0 Hz, 2H), 7.39 (t, J = 7.6 Hz, 1H), 2.66 (s, 3H). ^{13}C NMR (100 MHz, $CDCl_3$) δ (ppm): 198.2, 141.8, 140.2, 137.6, 131.8, 129.1, 129.0, 127.9, 127.2, 127.0, 26.8.

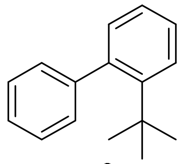

3pa

2-Tert-butylbiphenyl (3pa)[9] (0.034g, 16%; 0.090g, 43% isolated yield as colourless oil). ^{1}H NMR (400 MHz, $CDCl_3$) δ (ppm): 7.65 - 7.58 (m, 4H), 7.52 - 7.45 (m, 4H), 7.38 - 7.34 (m, 1H), 1.41 (s, 9H). ^{13}C NMR (100 MHz, $CDCl_3$) δ (ppm): 146.4, 142.2, 141.1, 130.0, 129.4, 128.8, 128.0, 127.7, 127.2, 126.8, 125.6, 125.4, 29.4, 24.4.

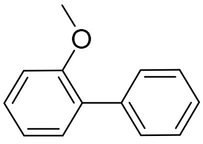

3qa

2-Methoxybiphenyl (3qa)[8] (0.055g, 30%; 0.120g, 65% isolated yield as colourless oil). ^{1}H NMR (400 MHz, $CDCl_3$) δ (ppm): 7.62 - 7.59 (m, 2H), 7.47 (t, J = 7.5 Hz, 2H), 7.40 - 7.37 (m, 3H), 7.12 - 7.04 (m, 2H), 3.86 (s, 3H). ^{13}C NMR (100 MHz, $CDCl_3$) δ (ppm): 156.5, 138.6, 130.9, 129.6, 128.6, 128.0, 127.0, 120.9, 111.3, 55.4.

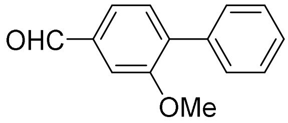

3ra

3-Methoxy-4-phenylbenzaldehyde (3ra)[10] (0.085g 40%; 0.182g 86% isolated yield as white solid), ^{1}H NMR (400 MHz, $CDCl_3$) δ (ppm): 10.02 (s, 1H), 7.57 - 7.53 (m, 3H), 7.51 - 7.39 (m, 5H), 3.90 (s, 3H). ^{13}C NMR (100 MHz, $CDCl_3$) δ (ppm): 191.9, 157.1, 137.3, 137.2, 131.4, 129.5, 128.2, 128.0, 124.5, 109.6, 55.8.

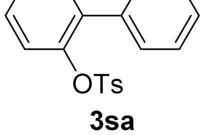

3sa

2-Tosyloxybiphenyl (3sa) (0.301g, 93% isolated yield as white solid). ^{1}H NMR (400 MHz, $CDCl_3$) δ (ppm): 7.52 (d, J = 8.0 Hz, 1H), 7.40 - 7.34 (m, 2H), 7.28 - 7.26 (m, 4H), 7.20 (d, J = 8.0 Hz, 2H), 7.15 - 7.13 (m, 2H), 7.67 (d, J = 8.0 Hz, 2H), 2.37 (s, 3H). ^{13}C NMR (100 MHz, $CDCl_3$) δ (ppm): 146.4, 144.6, 136.6, 135.4, 132.0, 131.1, 129.2, 128.6, 128.0, 127.9, 127.4, 127.2, 124.1, 21.6. HRMS (EI-TOF) **m/z:** (M^+) calcd for $C_{19}H_{16}O_3S$ 324.08; found 324.0818.

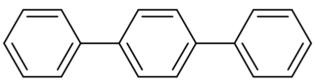

3ta

4-Phenylbiphenyl (3ta)[11] (0.216g, 94% isolated yield as white solid), M. P. 211-213 °C. ^{1}H NMR (400 MHz, CDCl$_3$) δ (ppm): δ 7.70 - 7.66 (m, 8H), 7.48 (t, J = 7.6 Hz, 4H), 7.40 - 7.36 (m, 2H). ^{13}C NMR (100 MHz, CDCl$_3$) δ (ppm): 140.9, 140.3, 129.0, 127.6, 127.5, 127.2.

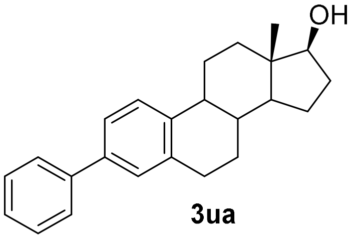

3ua

13-Methyl-3-phenyl-7,8,9,11,12,13,14,15,16,17-decahydro-6H-cyclopenta[a]phenanthren-17-ol (3ua)[12] (0.056g, 17%; 0.166g, 50% isolated yield as white solid), M. P. 172-173 °C. ^{1}H NMR (400 MHz, CDCl$_3$) δ (ppm): 7.60 - 7.58 (m, 2H), 7.45 - 7.39 (m, 4H), 7.35 - 7.31 (m, 2H), 3.76 (t, J = 8.4 Hz, 1H), 2.97 - 2.94 (m, 2H), 2.44 - 2.37 (m, 1H), 2.34 - 2.27 (m, 1H), 2.19 - 2.10 (m, 1H), 2.02 - 1.92 (m, 2H), 1.78 - 1.70 (m, 1H), 1.63 - 1.47 (m, 4H), 1.42 - 1.36 (m, 2H), 1.34 - 1.30 (m, 1H), 1.27 - 1.20 (m, 1H), 0.81 (s, 3H). ^{13}C NMR (100 MHz, CDCl$_3$) δ (ppm): 141.2, 139.6, 138.6, 137.2, 128.7, 127.8, 127.0, 127.0, 125.9, 124.5, 82.0, 50.2, 44.4, 43.3, 38.7, 36.8, 30.6, 29.7, 27.3, 26.2, 23.2, 11.1.

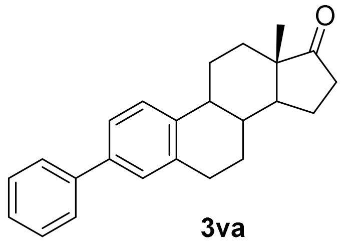

3va

13-Methyl-3-phenyl-6,7,8,9,11,12,13,14,15,16-decahydro-17H-cyclopenta[a]phenanthren-17-one (3va)[13] (0.132g, 40%; 0.278g, 84% isolated yield as white solid), M. P. 166-168 °C. ^{1}H NMR (400 MHz, CDCl$_3$) δ (ppm): 7.60 - 7.58 (m, 2H), 7.45 - 7.39 (m, 4H), 7.35 - 7.32 (m, 2H), 3.01 (dd, J = 8.8, 4.0 Hz, 2H), 2.57 - 2.46 (m, 2H), 2.40 - 2.34 (m, 1H), 2.21 - 1.98 (m, 4H), 1.71 - 1.50 (m, 6H), 0.94 (s, 3H). ^{13}C NMR (100 MHz, CDCl$_3$) δ (ppm): 221.0, 141.0, 138.9, 138.8, 136.9, 128.7, 127.8, 127.1, 127.0,

125.9, 124.6, 50.5, 48.0, 44.4, 38.2, 35.9, 31.6, 29.8, 29.6, 26.6, 25.8, 21.6, 13.9.

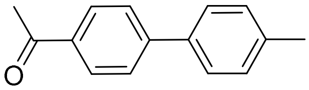

3ab

4-Acetyl-4'-methylbiphenyl (3ab)[1] (0.200g, 95% isolated yield as white solid), M.P. 118-120 °C. ^{1}H NMR (400 MHz, $CDCl_3$) δ (ppm): 8.02 (d, J = 8.4 Hz, 2H), 7.67 (d, J = 8.4 Hz, 2H), 7.54 (d, J = 8.0 Hz, 2H), 7.28 (d, J = 8.0 Hz, 2H), 2.64 (s, 3H), 2.42 (s, 3H). ^{13}C NMR (100 MHz, $CDCl_3$) δ (ppm): 197.8, 145.7, 138.3, 137.0, 135.6, 129.7, 129.0, 127.1, 127.0, 26.7, 21.2.

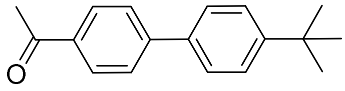

3ac

4-Acetyl-4'-tertiarybutylbiphenyl (3ac)[3] (0.232g, 92% isolated yield as white solid), M. P. 129-131 °C. ^{1}H NMR ($CDCl_3$, 400 MHz) δ (ppm): 8.03 (d, J = 8.4 Hz, 2H), 7.69 (d, J = 8.4 Hz, 2H), 7.59 (d, J = 8.4 Hz, 2H), 7.51 (d, J = 8.4 Hz, 2H), 2.64 (s, 3H), 1.38 (s, 9H). ^{13}C NMR (100 MHz, $CDCl_3$) δ (ppm): 197.9, 151.5, 145.7, 136.9, 135.6, 129.0, 127.0, 127.0, 126.0, 34.7, 31.4, 26.7.

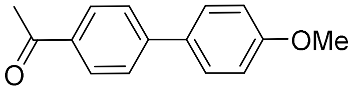

3ad

4-Acetyl-4'-methoxybiphenyl (3ad)[1] (0.203g, 90% isolated yield as white solid), M.P. 156-158 °C. ^{1}H NMR (400 MHz, $CDCl_3$) δ (ppm): 8.00 (d, J = 8.4 Hz, 2H), 7.64 (d, J = 8.4 Hz, 2H), 7.58 (d, J = 8.8 Hz, 2H), 7.00 (d, J = 8.8 Hz, 2H), 3.86 (s, 3H), 2.62 (s, 3H). ^{13}C NMR (100 MHz, $CDCl_3$) δ (ppm): 197.8, 160.0, 145.4, 135.3, 132.3, 129.0, 128.4, 126.6, 114.4, 55.4, 26.7.

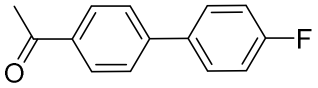

3ae

4-Acetyl-4'-fluorobiphenyl (3ae)[1] (0.197g, 92% isolated yield as white solid), M.P. 108-109 °C. ^{1}H NMR (400 MHz, $CDCl_3$) δ (ppm): 8.02 (d, J = 8.4 Hz, 2H), 7.64 - 7.57 (m, 4H), 7.15 (t, J = 8.4 Hz, 2H), 2.63 (s, 3H). ^{13}C NMR (100 MHz, $CDCl_3$) δ

(ppm): 197.7, 163.0 (d, J = 246.0 Hz), 144.8, 136.1 (d, J = 4.0 Hz), 129.0, 128.9, 127.1, 115.9 (d, J = 22.0 Hz), 26.7.

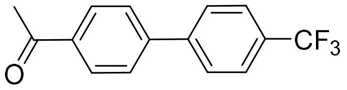

3af

4-Acetyl-4'-trifluoromethylbiphenyl (3af)[1] (0.196g, 74% isolated yield as white solid), M.P. 122-123 °C. ^{1}H NMR ($CDCl_3$, 400 MHz) δ (ppm): 8.06 (d, J = 8.4 Hz, 2H), 7.72 (s, 4 H), 7.69 (d, J = 8.4 Hz, 2H), 2.65 (s, 3H). ^{13}C NMR (100 MHz, $CDCl_3$) δ (ppm): 197.7, 144.2, 143.4, 136.6, 130.2 (q, J = 32.4 Hz), 129.8, 129.1, 127.7, 127.5, 125.9 (q, J = 3.7 Hz), 122.8, 26.8.

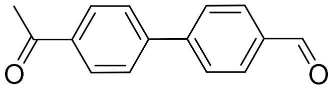

3ag

4-Acetyl-4'-formylbiphenyl (3ag)[11] (0.179g, 80% isolated yield as white solid), M. P. 145-147 °C. ^{1}H NMR (400 MHz, $CDCl_3$) δ (ppm): 10.07 (s, 1H), 8.06 (d, J = 8.4 Hz, 2H), 7.98 (d, J = 8.4 Hz, 2H), 7.78 (d, J = 8.0 Hz, 2H), 7.72 (d, J = 8.0 Hz, 2H), 2.65 (s, 3H). ^{13}C NMR (100 MHz, $CDCl_3$) δ (ppm): 197.7, 191.8, 145.7, 144.2, 136.7, 135.8, 130.4, 129.1, 127.9, 127.6, 26.8.

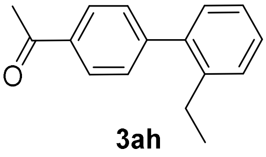

3ah

4-Acetyl-2'-ethylbiphenyl (3ah)[11] (0.204g, 91% isolated yield as yellow oil). ^{1}H NMR ($CDCl_3$, 400 MHz) δ (ppm): 7.90 (d, J = 8.0 Hz, 2H), 7.31 (d, J = 8.0 Hz, 2H), 7.24 - 7.21 (m, 2H), 7.17 - 7.13 (m, 1H), 7.08 (d, J = 7.6 Hz, 1H), 2.54 (s, 3H), 2.48 (q, J = 7.6 Hz, 2H), 0.99 (t, J = 7.6 Hz, 3H). ^{13}C NMR (100 MHz, $CDCl_3$) δ (ppm): 198.0, 147.1, 141.5, 140.5, 135.6, 129.7, 129.5, 128.8, 128.2, 128.1, 125.8, 26.7, 26.1, 15.7.

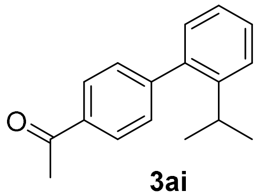

3ai

4-Acetyl-2'-isopropylbiphenyl (3ai)[14] (0.207g, 87% isolated yield as yellow oil). ^{1}H NMR (400 MHz, CDCl$_3$) δ (ppm): 7.94 (d, J = 8.4 Hz, 2H), 7.36 - 7.28 (m, 4H), 7.18 - 7.14 (m, 1H), 7.10 - 7.07 (m, 1H), 2.93 (sept, J = 6.8 Hz, 1H), 2.58 (s, 3H), 1.09 (d, J = 6.8 Hz, 6H). ^{13}C NMR (100 MHz, CDCl$_3$) δ (ppm): 197.9, 147.2, 146.2, 139.9, 135.6, 129.6, 129.6, 128.3, 128.2, 125.8, 125.5, 29.5, 26.7, 24.2.

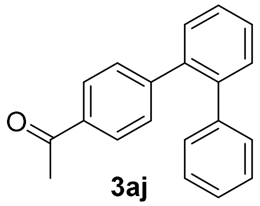

3aj

4-Acetylphenyl-2-phenylbenzene (3aj)[14] (0.196g, 72% isolated yield as white solid), M. P. 94-95 °C. ^{1}H NMR (400 MHz, CDCl$_3$) δ (ppm): 7.72 (d, J = 8.4 Hz, 2H), 7.37 - 7.34 (m, 4H), 7.16 - 7.12 (m, 5H), 7.05 - 7.03 (m, 2H), 2.48 (s, 3H). ^{13}C NMR (100 MHz, CDCl$_3$) δ (ppm): 198.0, 146.7, 141.0, 140.7, 139.4, 135.1, 130.8, 130.4, 130.1, 129.9, 128.2, 128.1, 128.0, 127.7, 126.8, 26.7.

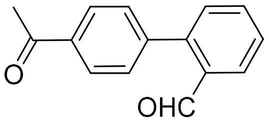

3ak

4-Acetyl-2'-formylbiphenyl (3ak)[7] (0.152g, 68% isolated yield as white solid), M.P. 89-91 °C. ^{1}H NMR (400 MHz, CDCl$_3$) δ (ppm): 9.95 (s, 1H), 8.06 - 8.02 (m, 3H), 7.68 - 7.64 (m, 1H), 7.56 - 7.51 (m, 1H), 7.55 - 7.47 (m, 3H), 2.66 (s, 3H). ^{13}C NMR (100 MHz, CDCl$_3$) δ (ppm): 197.6, 191.7, 144.5, 142.6, 136.5, 133.7, 133.6, 130.6, 130.3, 128.5, 128.4, 128.1, 26.8.

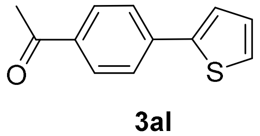

3al

4-(2'-Thienyl)acetophenone (3al)[3] (0.160g, 79% isolated yield as yellow solid), M.P. 115-117 °C. ^{1}H NMR (400 MHz, CDCl$_3$) δ (ppm): 7.96 (d, J = 8.4 Hz, 2H), 7.69 (d, J

= 8.4 Hz, 2H), 7.43 (d, J = 3.6 Hz, 1H), 7.37 (d, J = 4.8 Hz, 1H), 7.13 - 7.11 (m, 1H), 2.61 (s, 3H). ^{13}C NMR (100 MHz, $CDCl_3$) δ (ppm): 197.4, 143.0, 138.8, 135.8, 129.1, 128.4, 126.5, 125.7, 124.6, 26.6.

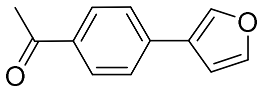

3am

4-(3'-Furyl)acetophenone (3am)[11] (0.140g, 75% isolated yield as white solid), M.P. 83-85 ºC. ^{1}H NMR (400 MHz, $CDCl_3$) δ (ppm): 7.96 (d, J = 8.4 Hz, 2H), 7.82 (s, 1H), 7.56 (d, J = 8.4 Hz, 2H), 7.51 - 7.50 (m, 1H), 6.74 (d, J = 0.9 Hz, 1H), 2.61 (s, 3H). ^{13}C NMR (100 MHz, $CDCl_3$) δ (ppm): 197.5, 144.2, 139.7, 137.2, 135.6, 129.1, 125.8, 108.6, 26.5.

References

1. Ren, C.; Zeng, J.S.; Zou, G. Nickel-catalyzed cross-coupling of O, N-chelated diarylborinates with aryl chlorides and mesylates. *New J. Chem.* **2019,** *43*, 1589-1596.

2. Zhang. G. Efficient Protocol for the Phosphine-Free Suzuki-miyaura reaction catalyzed by palladium on carbon at room temperature. *Synthesis.* **2005**, *4*, 537-542.

3. Chen, L.S.; Lang, H. Y.; Fang, L.; Zhu, M.Y.; Liu, J. Q.; Yu, J.J.; Wang, L.M. Nickel-catalyzed one-Pot suzuki-miyaura cross-coupling of phenols and arylboronic acids mediated by N,N-ditosylaniline. *Eur. J. Org. Chem.* **2014**, 4953-4957.

4. Xu, T.F.; Lu, P.; Wohlrab, S.; Chen, W.X.; Springer, A.; Wu, X.F.; Lu, W.Y. In situ grown palladium nanoparticles on polyester fabric as easy-separable and recyclable catalyst for suzuki-miyaura reaction. *Cat. Commun.* **2021**, *157*, 106328-106333.

5. Schmidt, A.; Rahimia, A. A versatile catalyst system for suzuki-miyaura syntheses of sterically hindered biaryls employing a cyclobutene-1,2-bis(imidazolium)salt. *Chem. Commun.* **2010**, *46*, 2995-2997.

6. Zhang, D.L.; Wu, F.S.; Wan, Z.J.; Wang, Y.C.; He, X.; Guo, B.; You, H.Z.; Chen, F.E. A palladium polyaniline complex: a simple and efficient catalyst for batch and flow suzuki-miyaura cross-couplings. *Chem. Commun.* **2022**, *58*, 10845-10848.

7. Woolard, K.J.; Sandala, J.L.; Melander, R.J.; Gunn, J. S. Melander, C.; Development of small molecules that work cooperatively with ciprofloxacin to clear salmonella biofilms in a chronic gallbladder carriage model. *Eur. J. Med. Chem.* **2022**, *232*, 114203-114211.

8. Zhang, L.Y.; Long, S.Y.; Jiao, H. B.; Liu, Z.Y.; Zhang, P.; Lei, A.W.; Gong, W.; Pei, X.L. Cellulose derived Pd nano-catalyst for efficient catalysis. *RSC Adv*. **2022**, *12*, 18676-18684.

9. Watson, M.B.; Rath, N.P.; Mirica, L.M. Oxidative C-C bond formation reactivity of organometallic Ni(II), Ni(III), and Ni(IV) complexes. *J. Am. Chem. Soc*. **2017**,*139*, 35-38.

10. Ohgi, A.; Semba, K.; Hiyama, T.; Nakao, Y. Silicon-based cross-coupling of aryl tosylates by cooperative palladium/copper catalysis. *Chem. Lett*. **2016**, *45*, 973-975.

11. Guo, D.S.; Shi, W.J.; Zou, G. Suzuki coupling of activated aryltriazenes for practical synthesis of biaryls from anilines. *Adv. Synth. Catal.* **2022**, *364*, 2438-2442.

12. Rühl, P.; Rosato, A.S.; Urban, N.; Gerndt, S.; Tang, R. Abrahamian, C.; Leser, C.; Sheng, J.S.; Jha, A.; Vollmer, G.; Schaefer, M.; Bracher, F.; Grimm, C. Estradiol analogs attenuate autophagy, cell migration and invasion by direct and selective inhibition of trpml1, independent of estrogen receptors. *Scientifc Reports*. **2021**, *11*, 8313-8326.

13. Song, C.l.; Dong, X.; Wang, Z.J.; Liu, K.; Chiang, C.W.; Lei, A. Visible-light induced [4+2] annulation of thiophenes and alkynes to construct benzene rings. *Angew. Chem., Int. Ed.* **2019**, *58*, 12206-12210.

14. Hagiwara, H.; Sato, K.; Hoshi, T.; Suzuki, T.; A highly sustainable and active catalyst for suzuki-miyaura reaction: palladium-supported ionic liquid catalyst (SILC) coated with polymer. *Synlett.* **2011**, *17*, 2545-2550.

^{1}H and ^{13}C NMR spectra of the biphenyl compounds

4-Acetylbiphenyl 3aa

^{1}H NMR

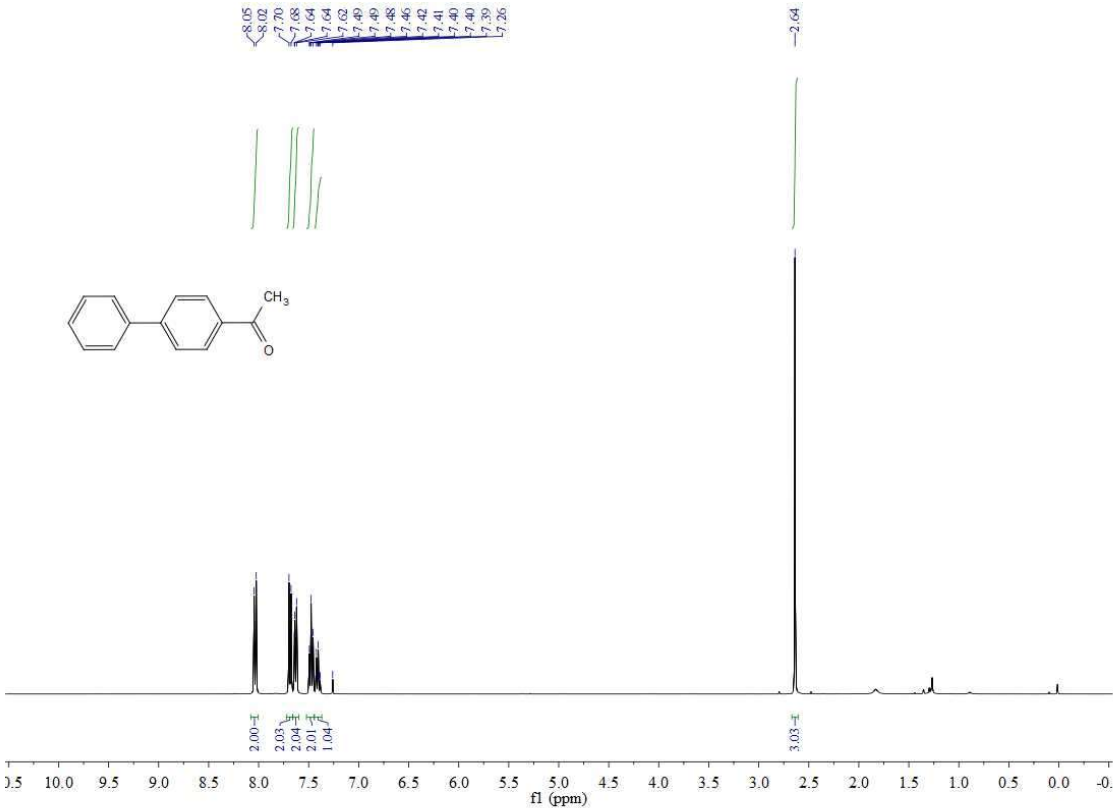

^{13}C NMR

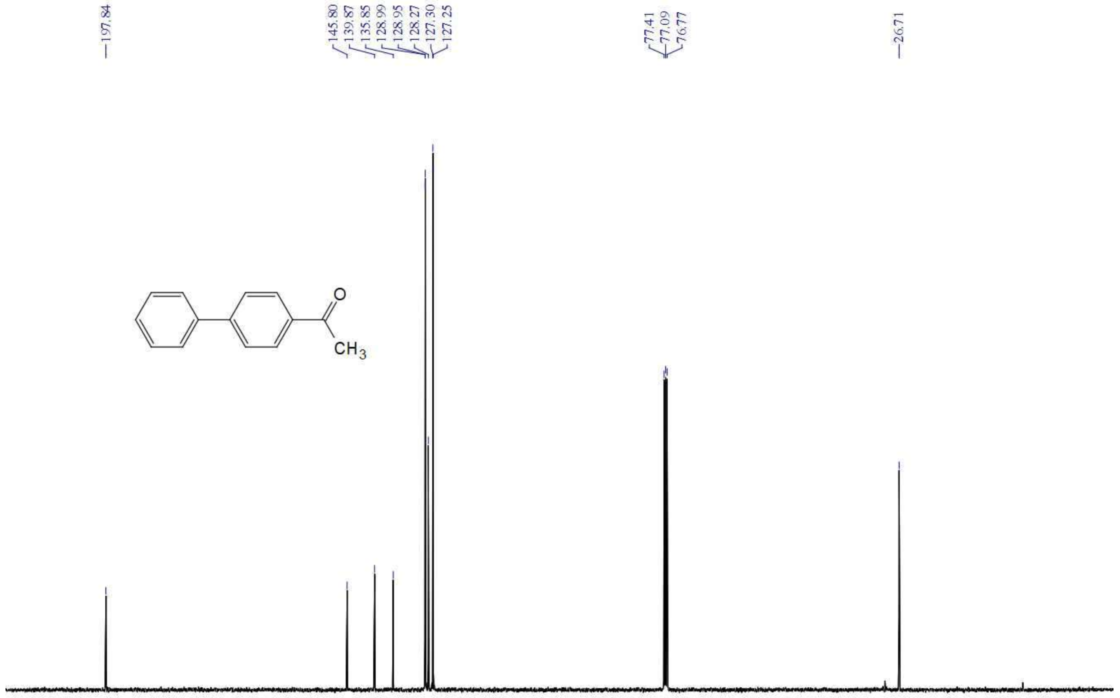

4-Ethoxycarbonylbiphenyl 3ba

^{1}H NMR

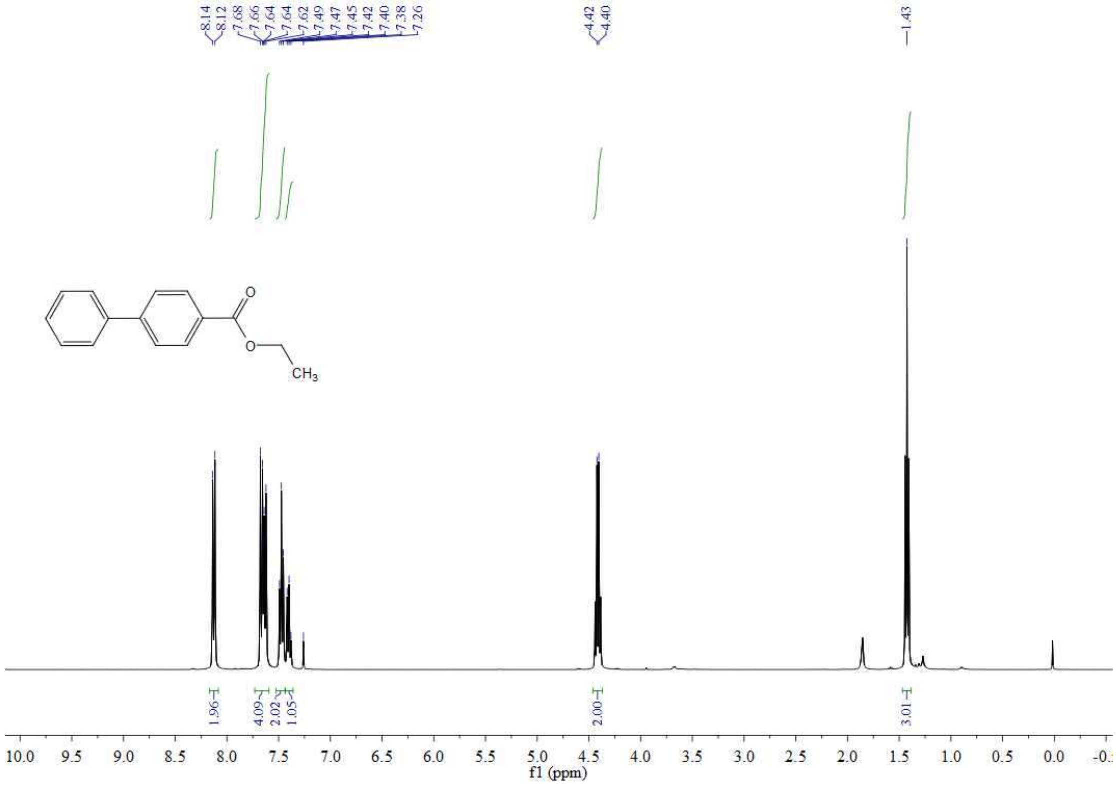

^{13}C NMR

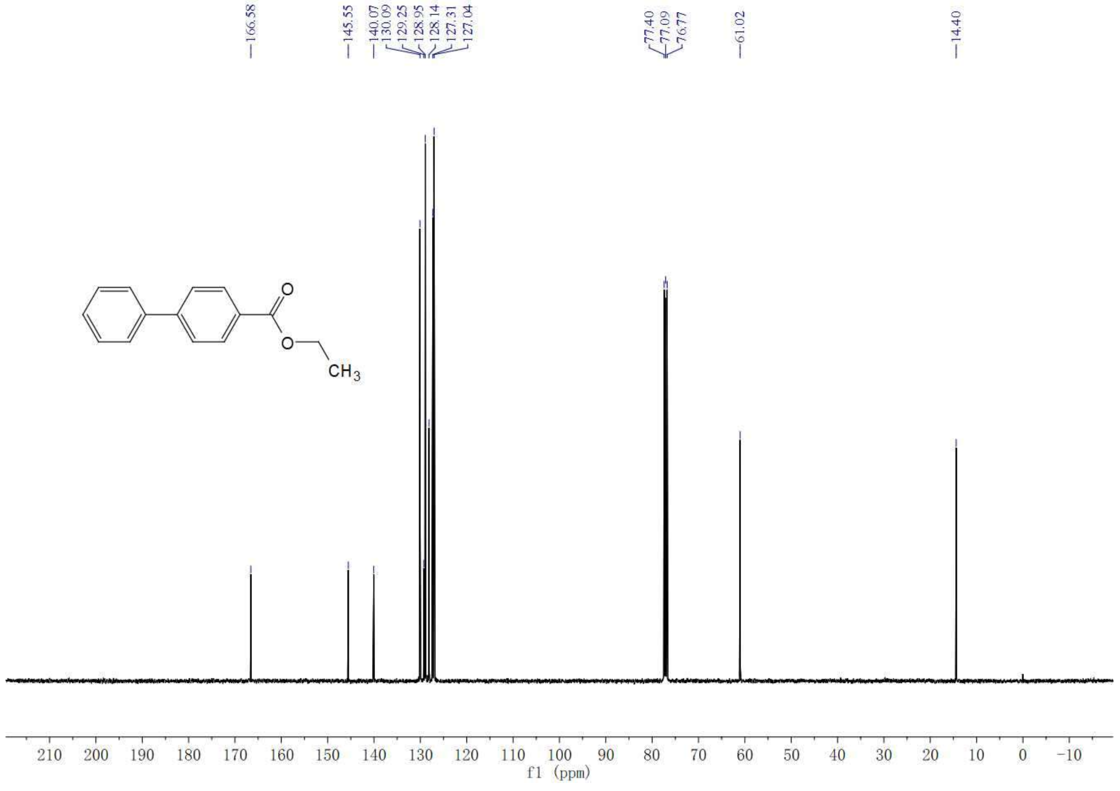

4-Cyanobiphenyl 3ca

^{1}H NMR

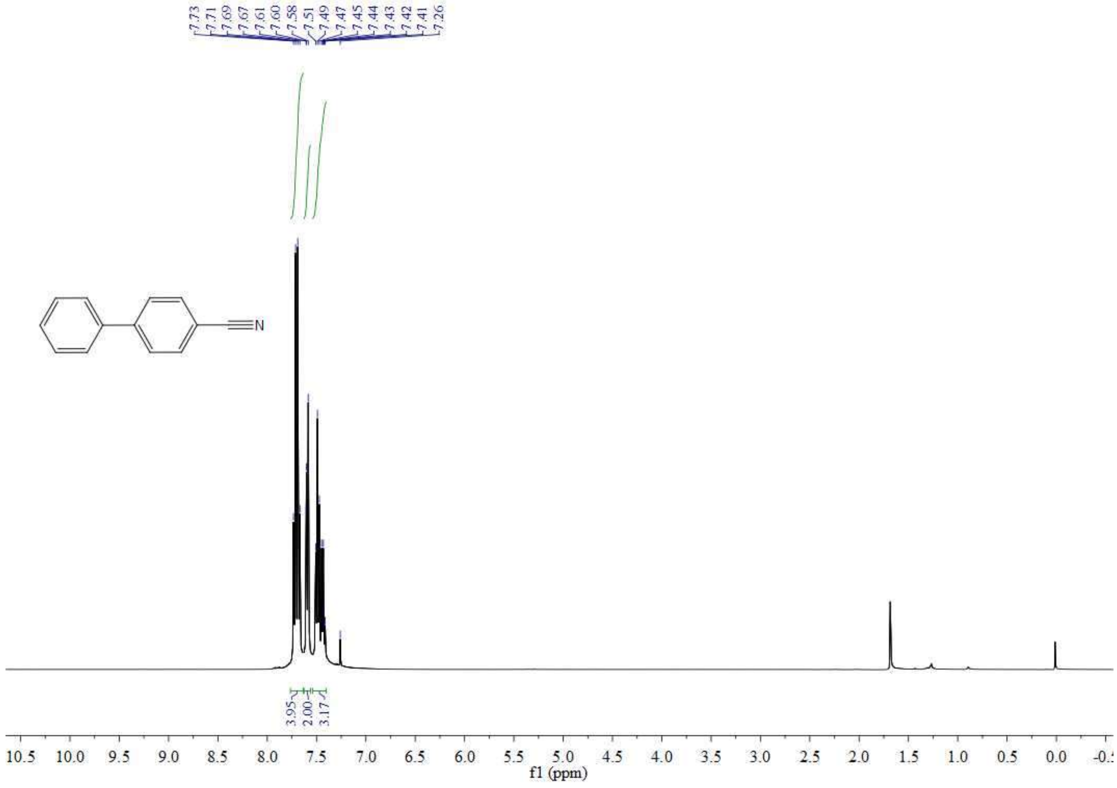

^{13}C NMR

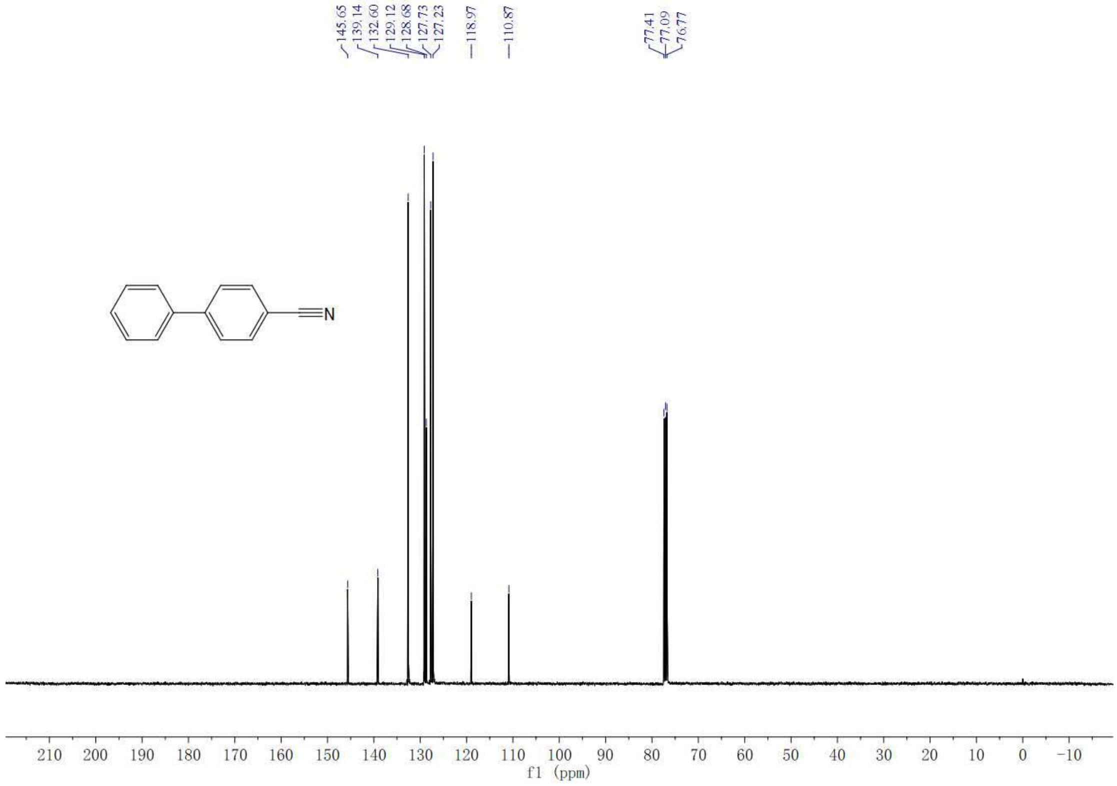

4-Nitrobiphenyl 3da

^{1}H NMR

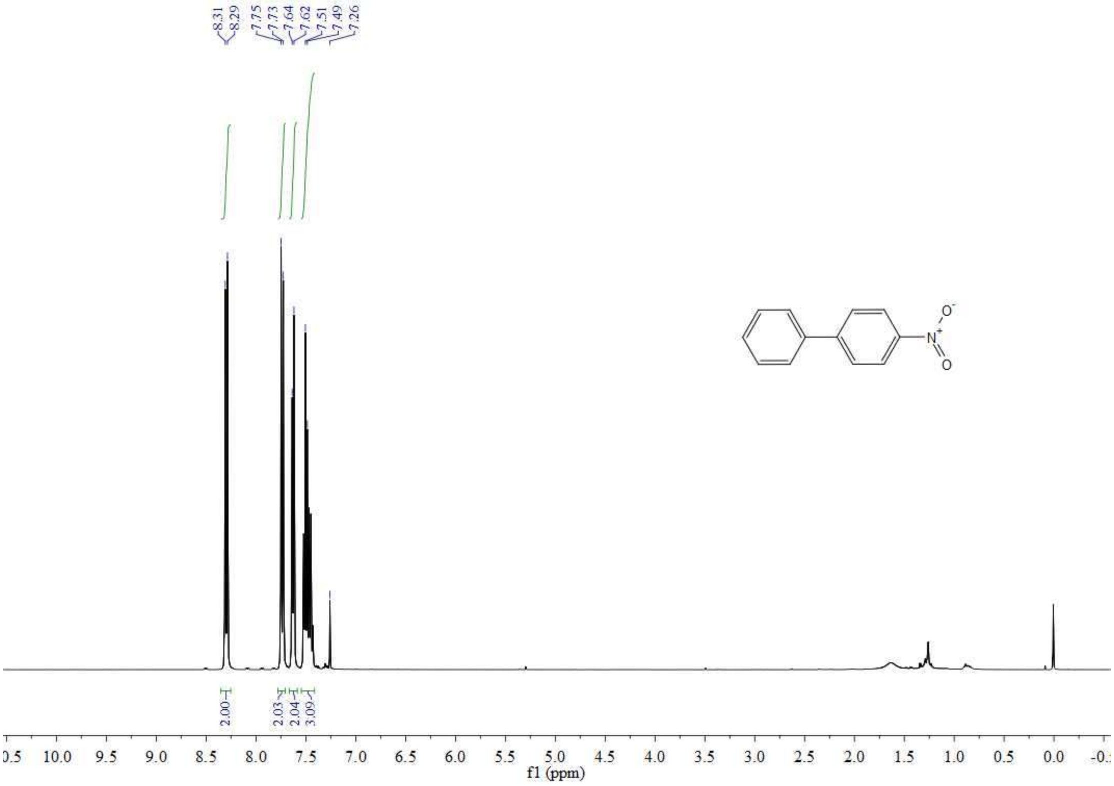

^{13}C NMR

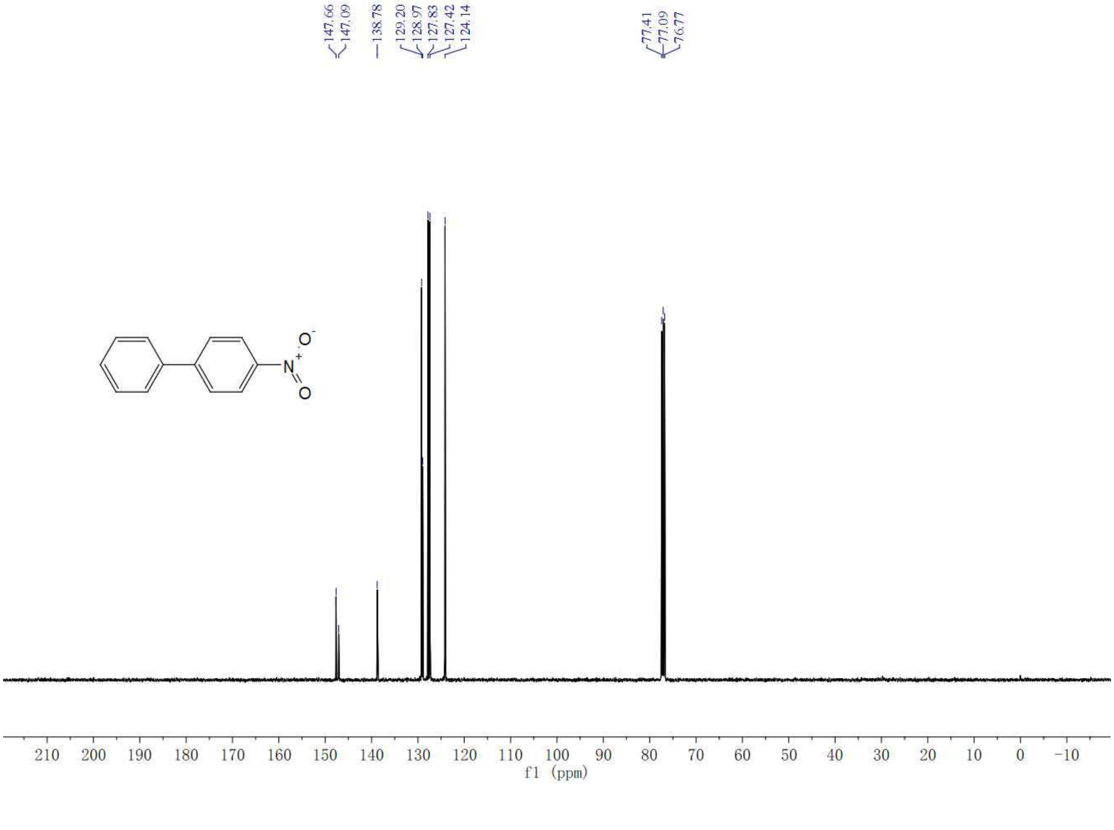

4-Aldehydebiphenyl 3ea

^{1}H NMR

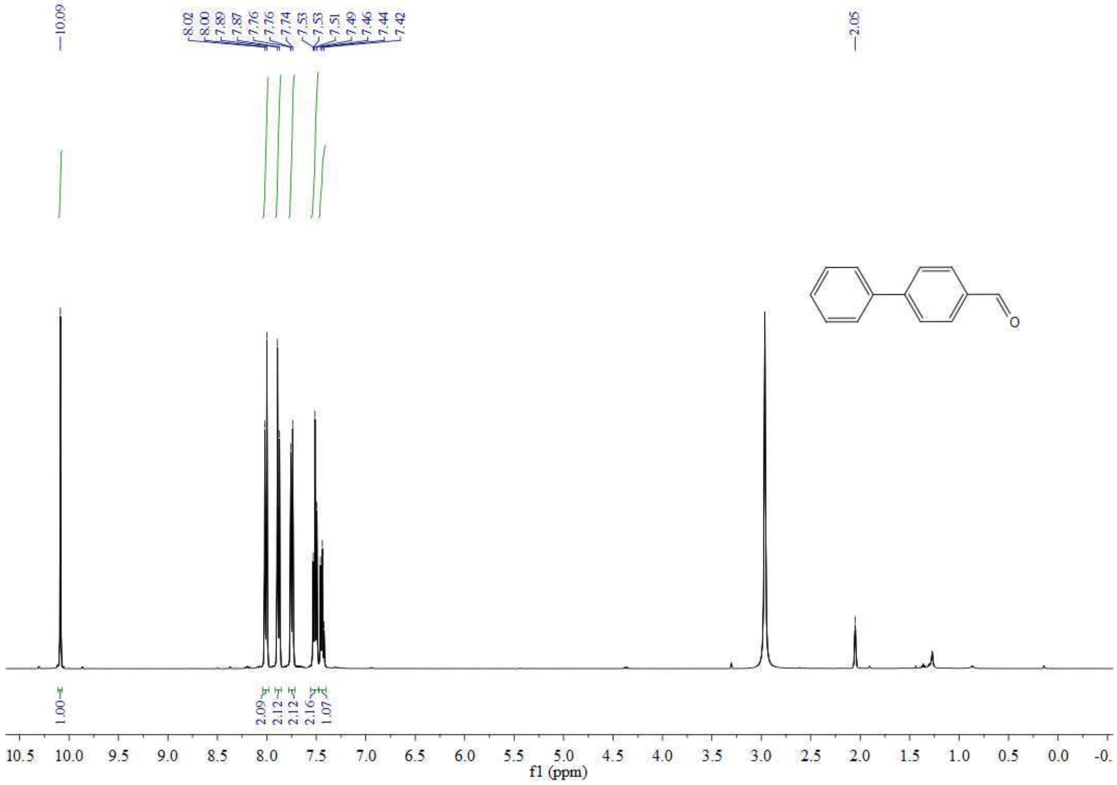

^{13}C NMR

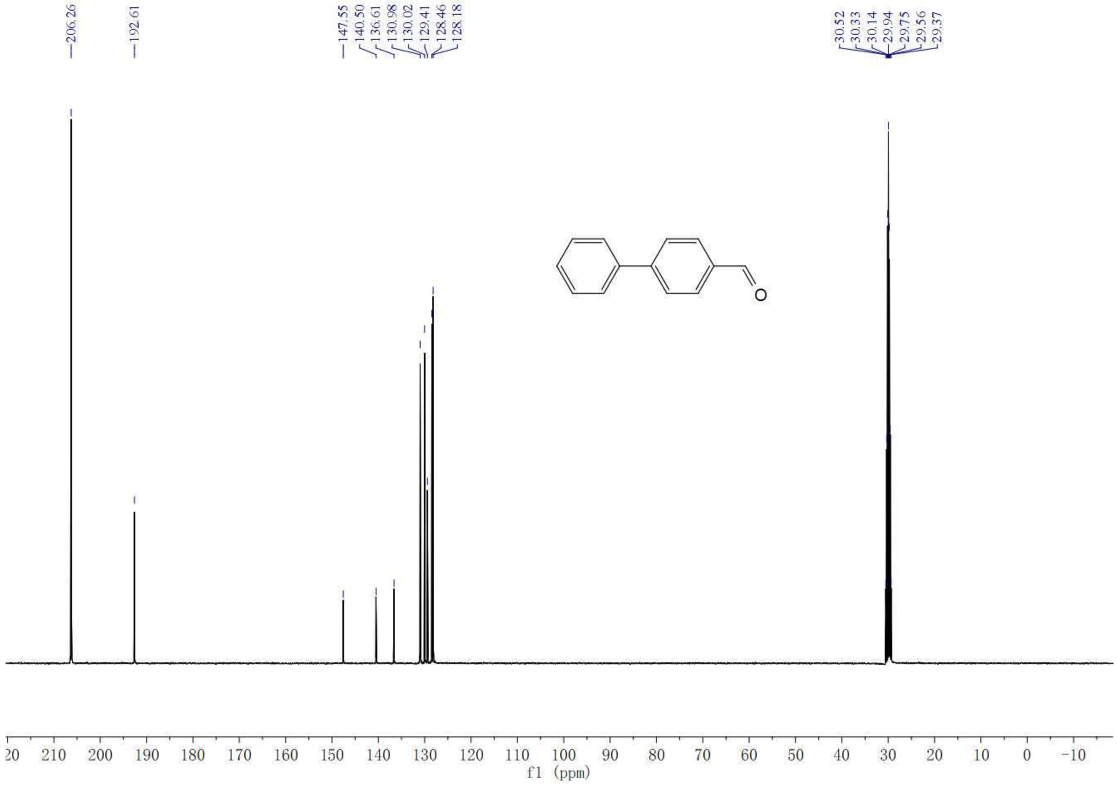

4-Fluorobiphenyl 3fa

^{1}H NMR

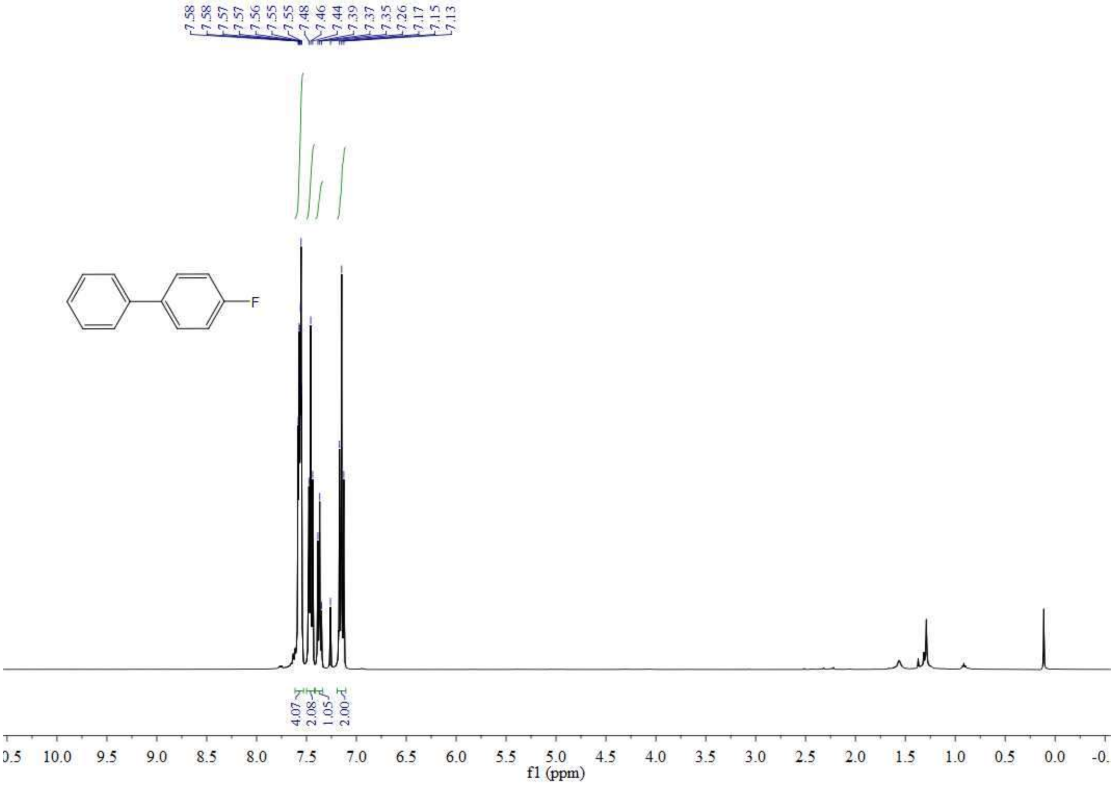

^{13}C NMR

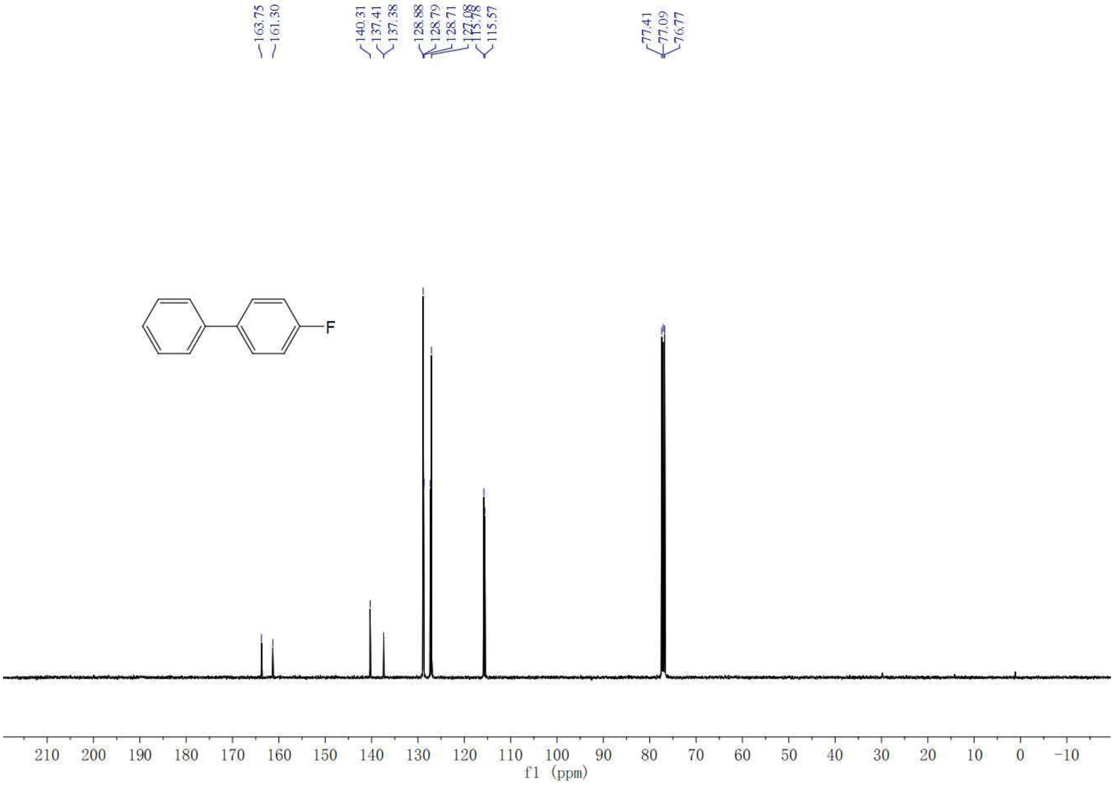

4-Chlorobiphenyl 3ga

^{1}H NMR

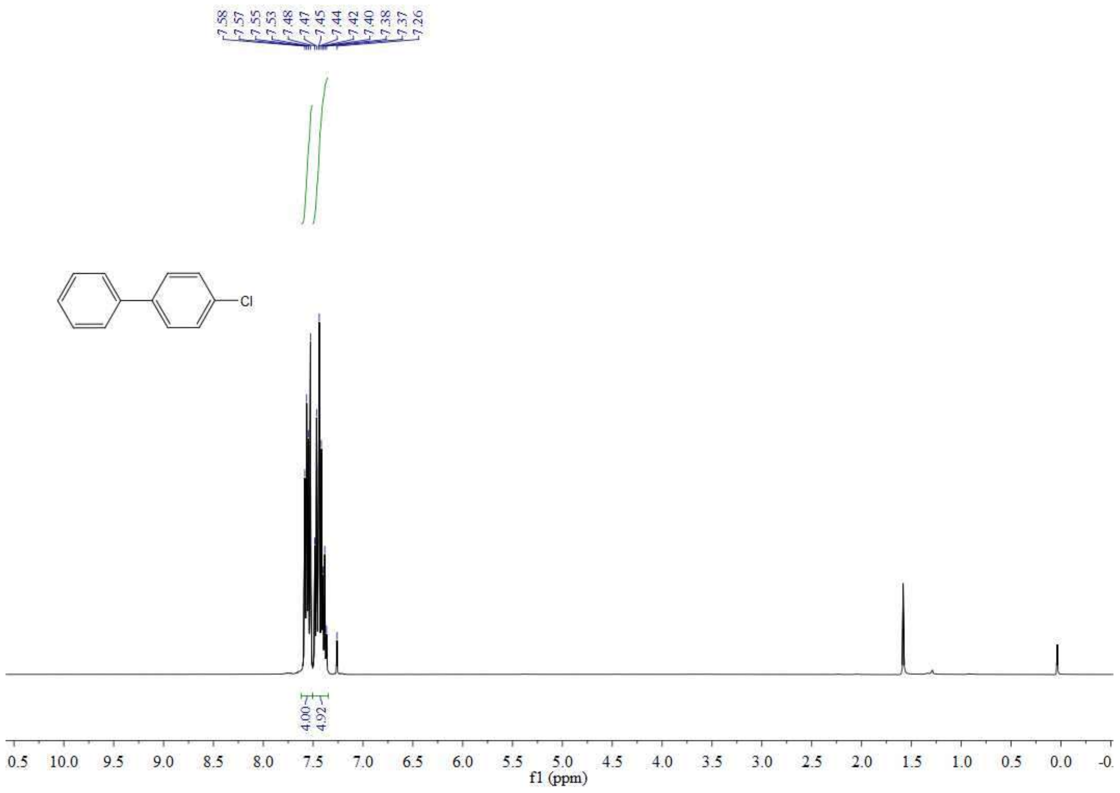

^{13}C NMR

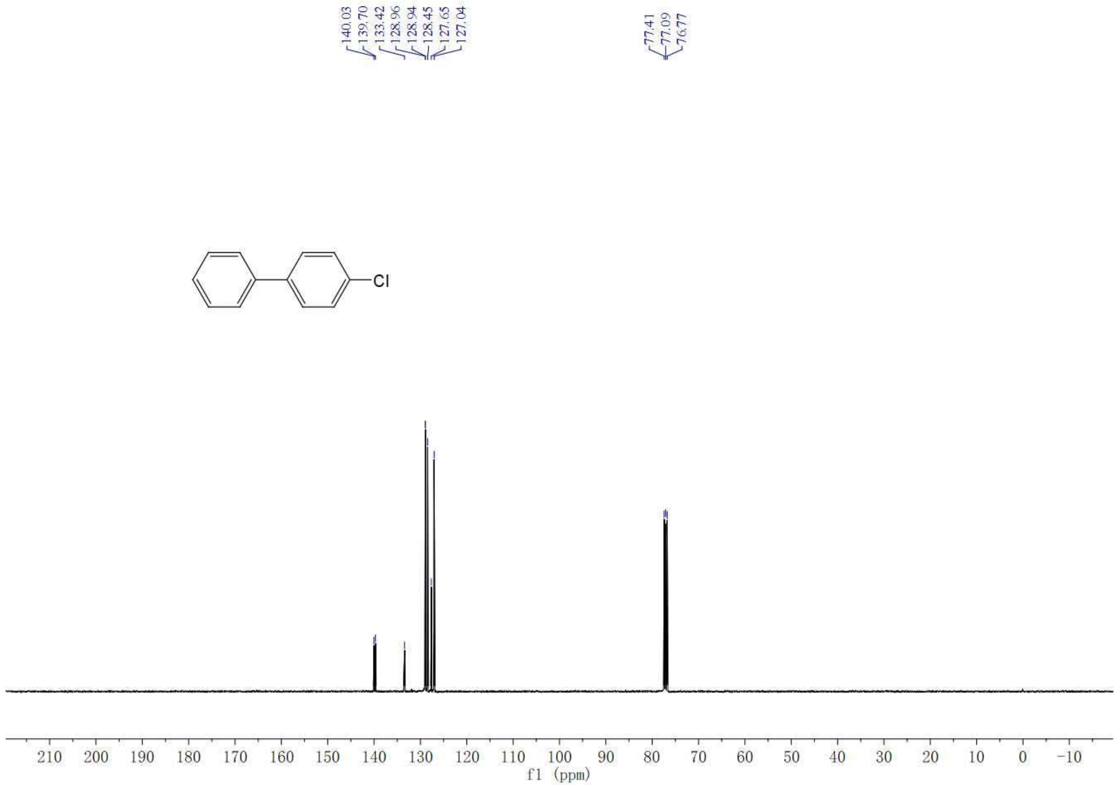

4-Isopropylbiphenyl 3ha

^{1}H NMR

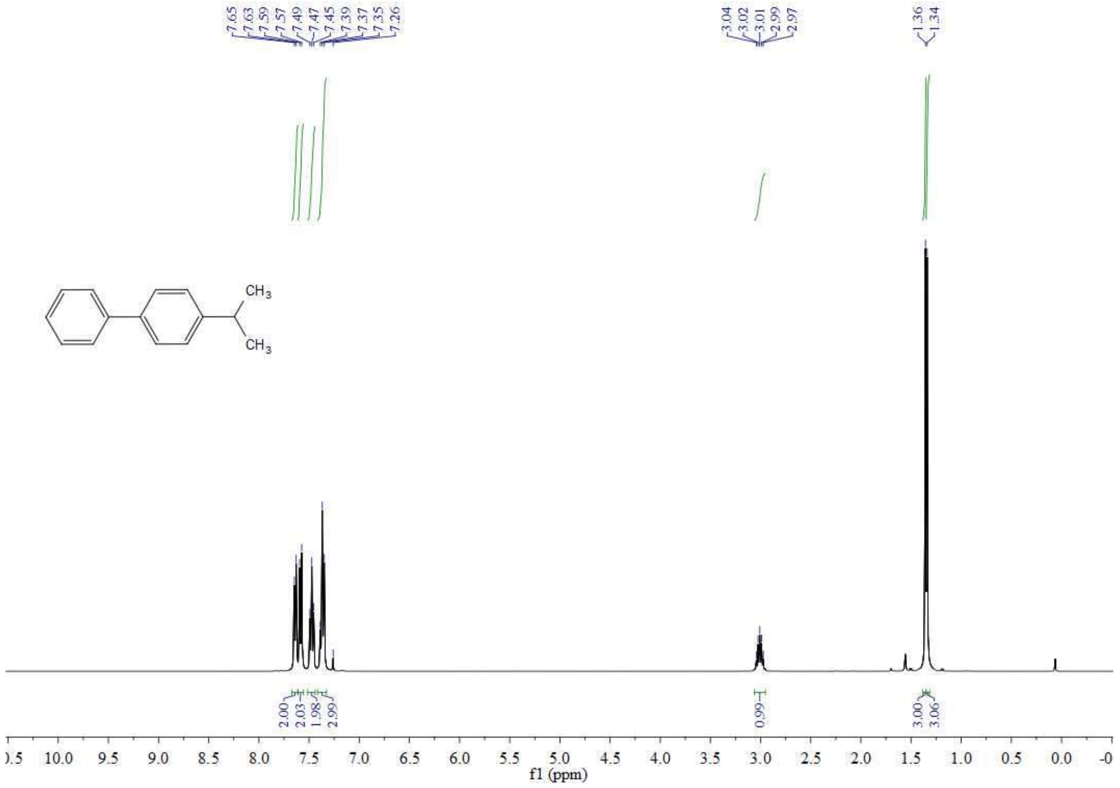

^{13}C NMR

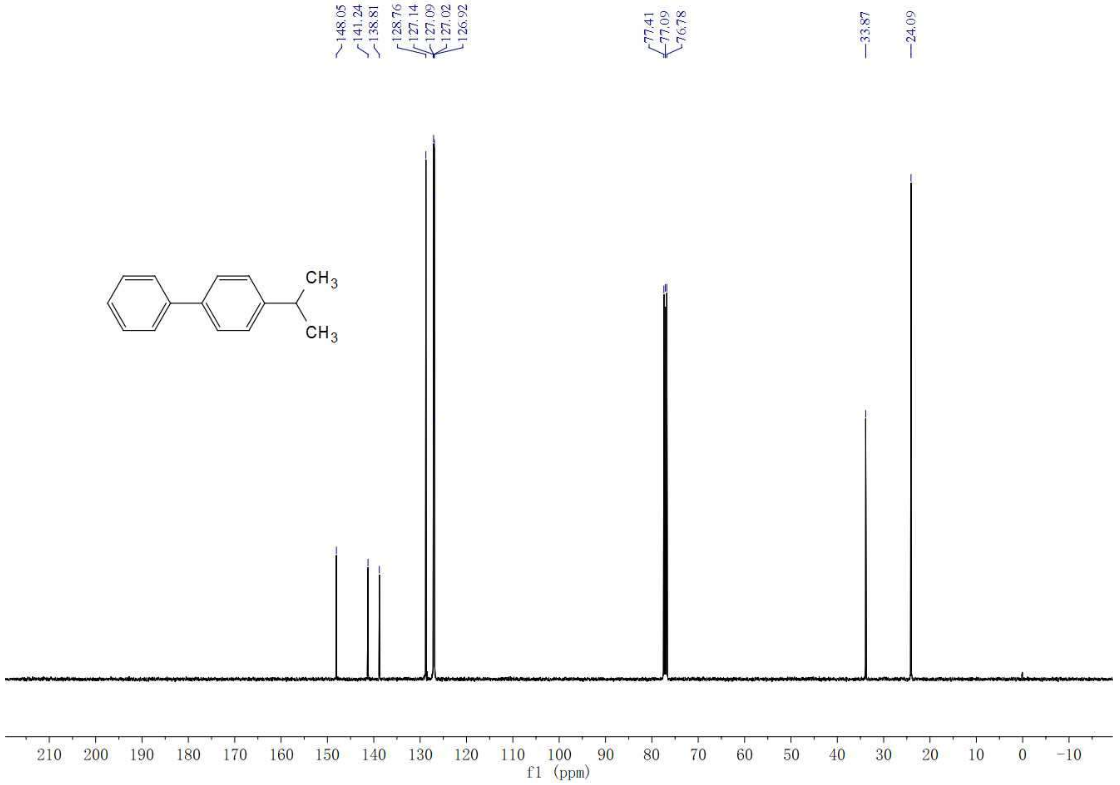

4-Methoxybiphenyl 3ia

^{1}H NMR

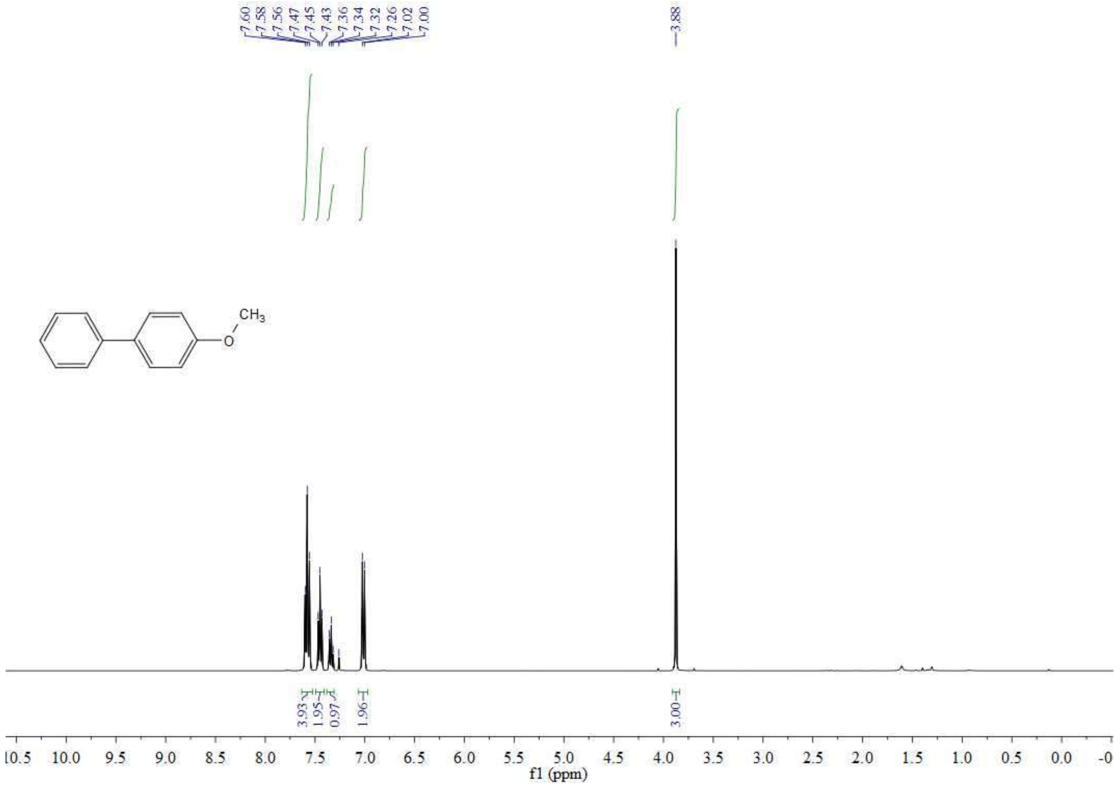

^{13}C NMR

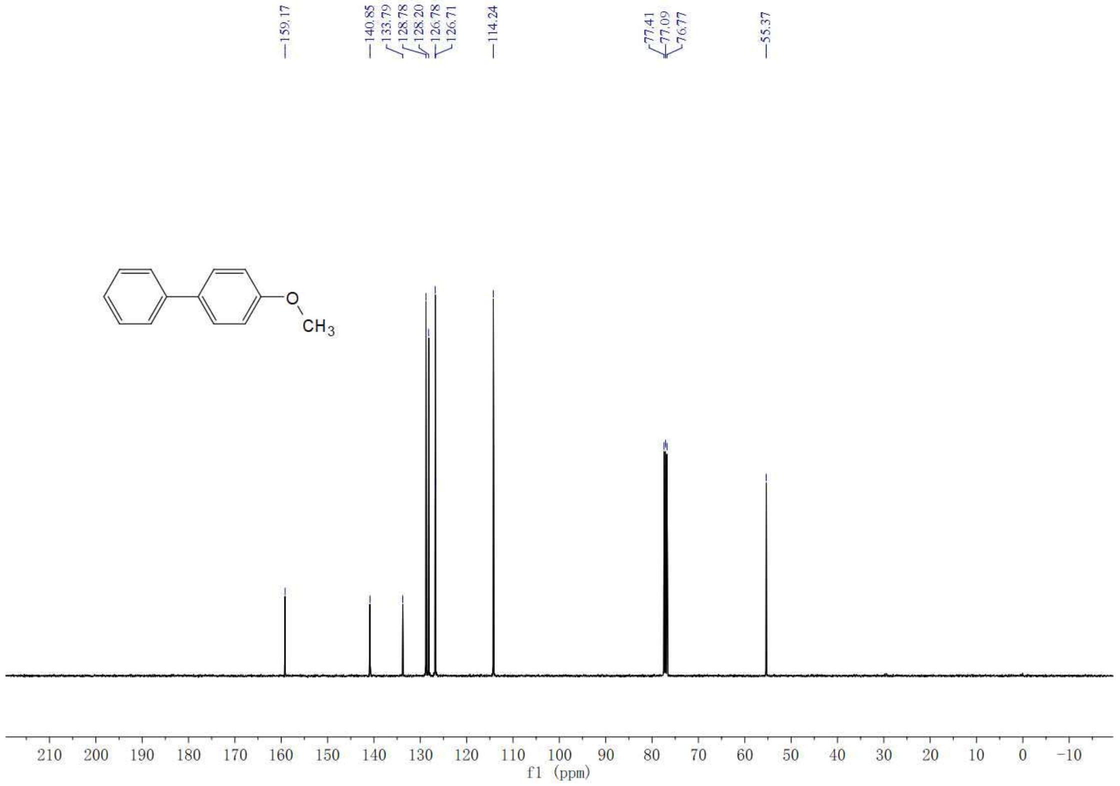

4-Phenylaniline 3ja

^{1}H NMR

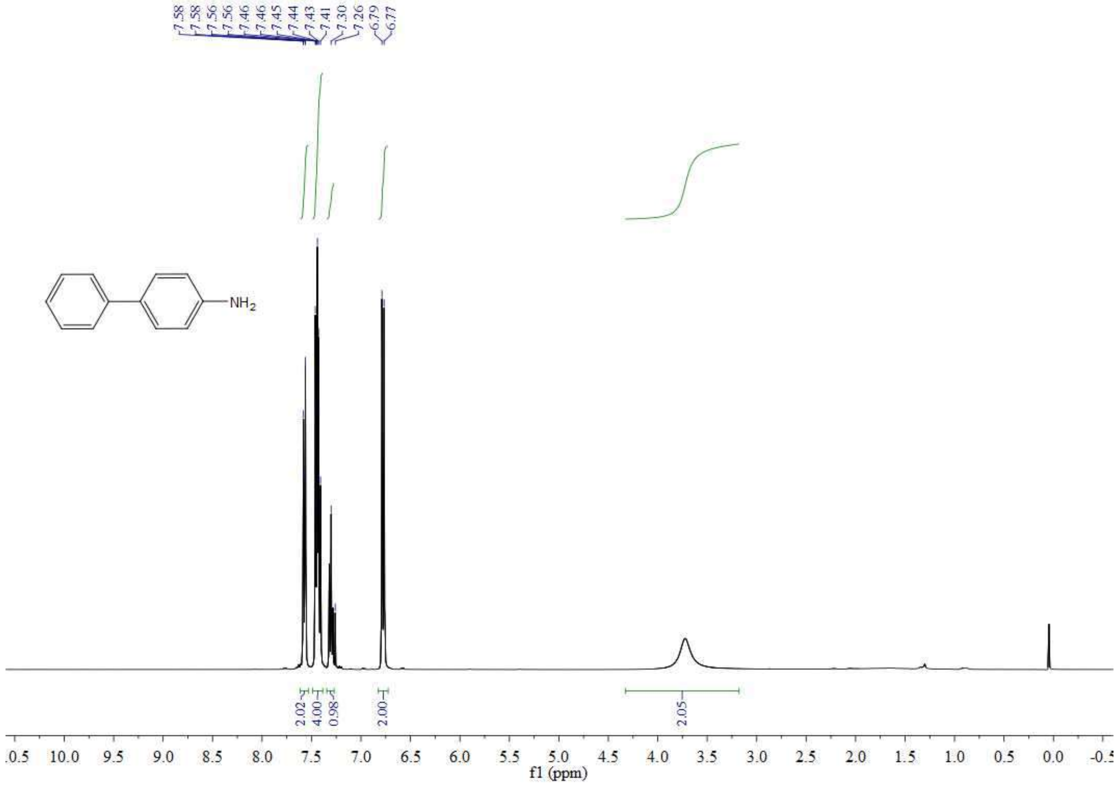

^{13}C NMR

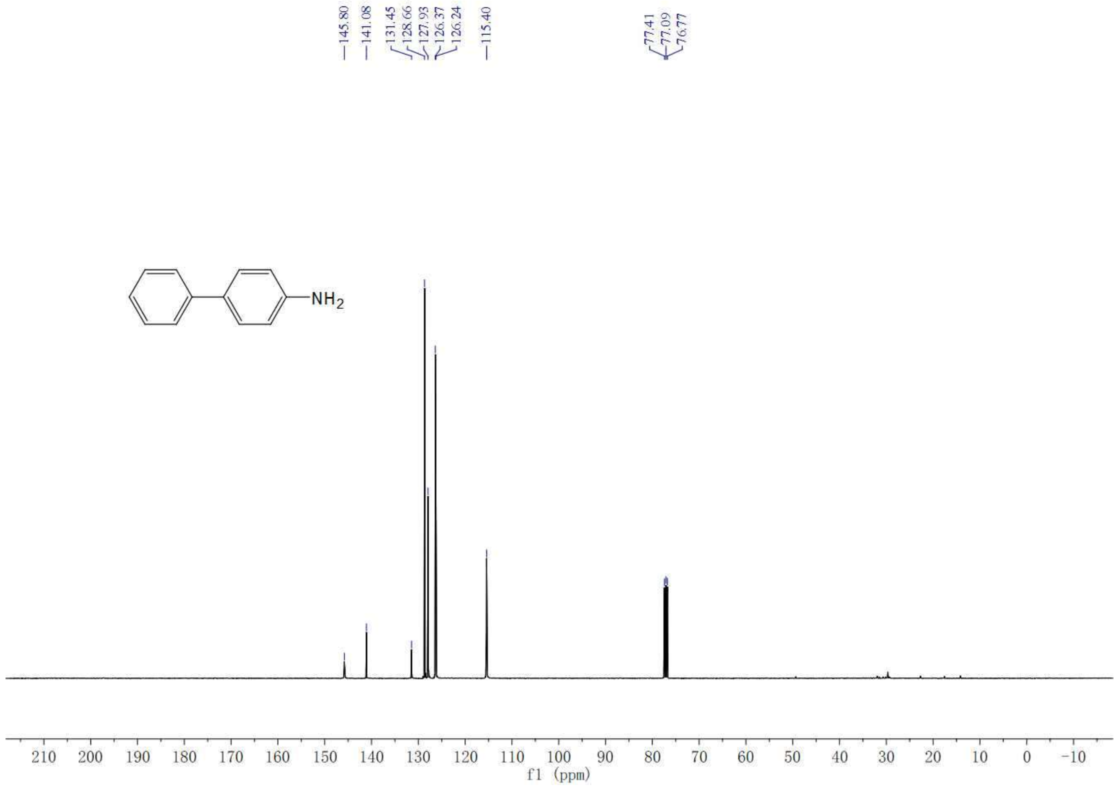

1-Phenylnaphthalene 3ka

^{1}H NMR

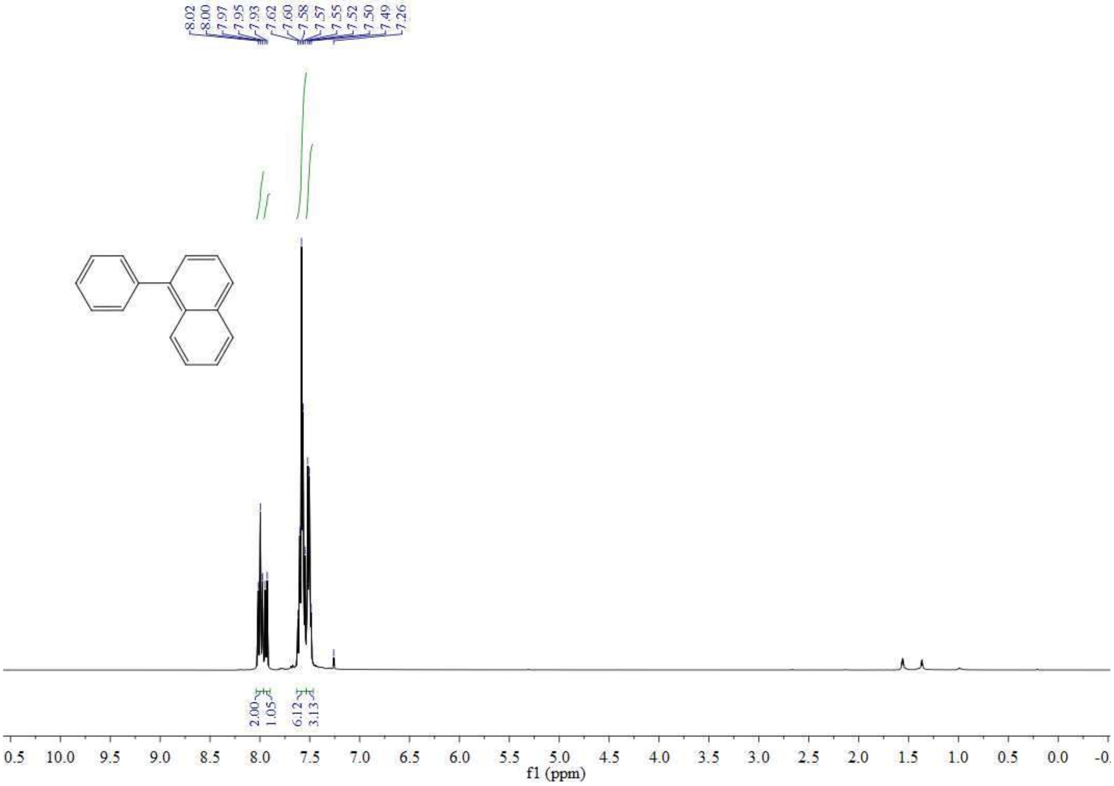

^{13}C NMR

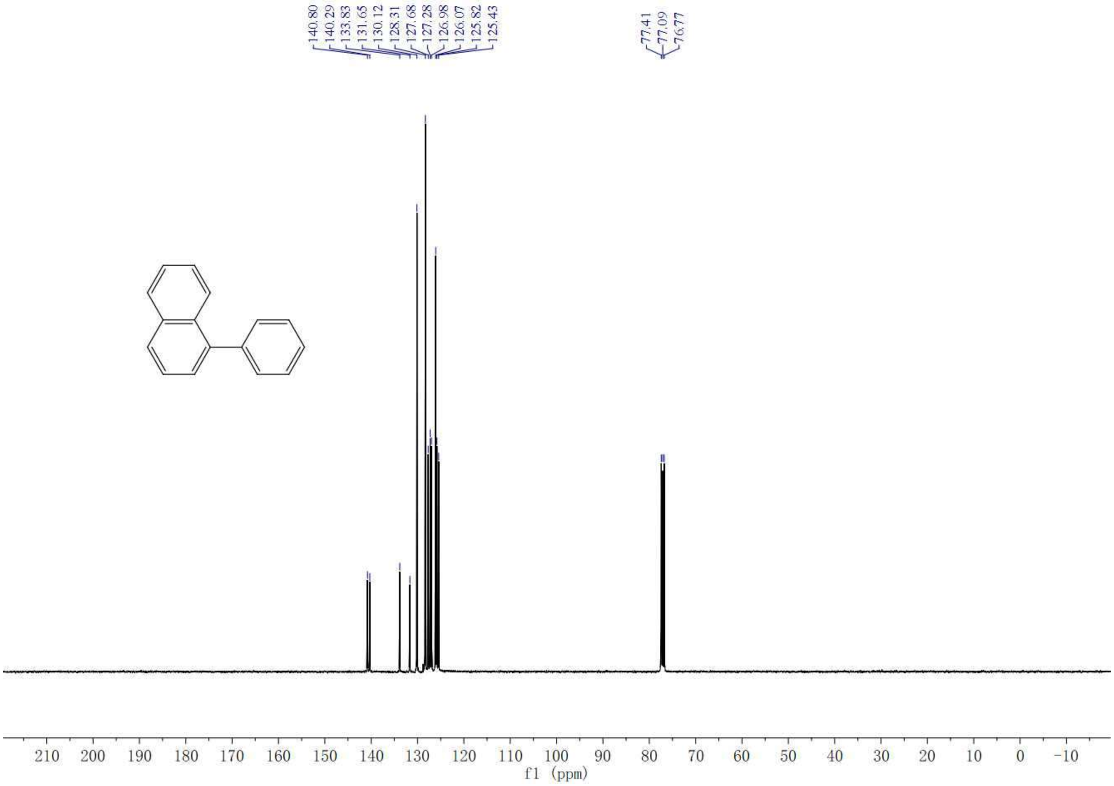

1-Biphenyl-4-ethanol 3la

^{1}H NMR

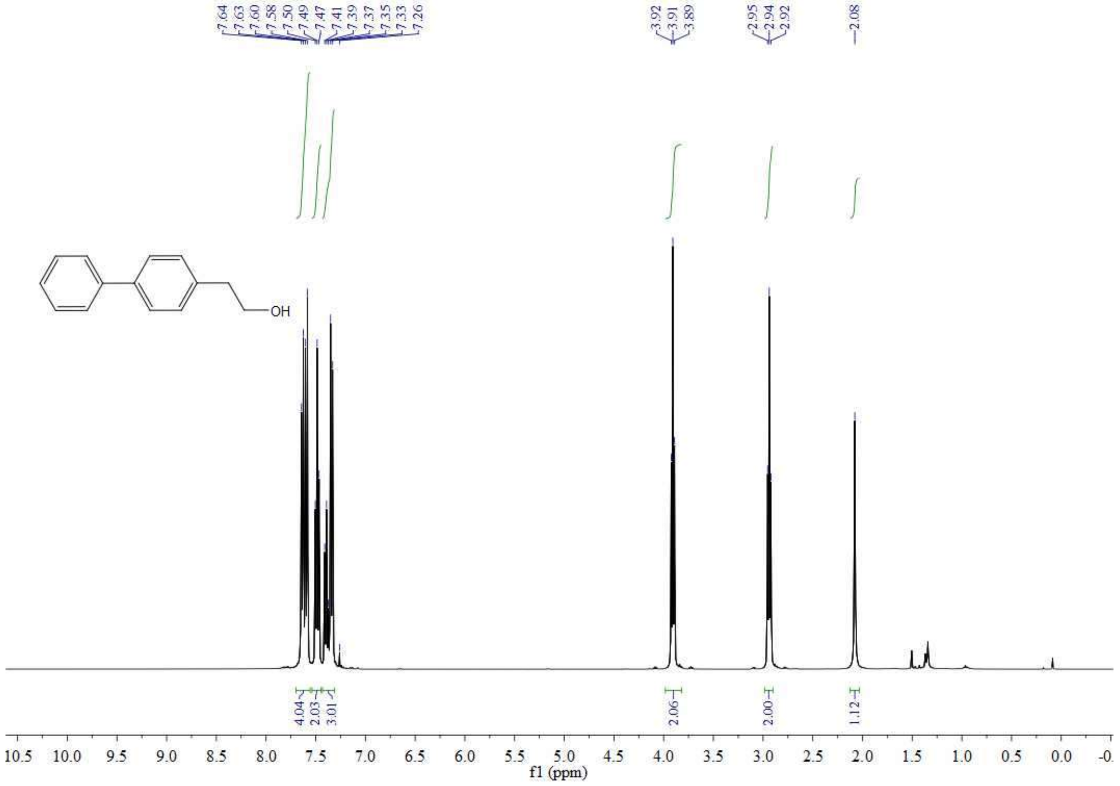

^{13}C NMR

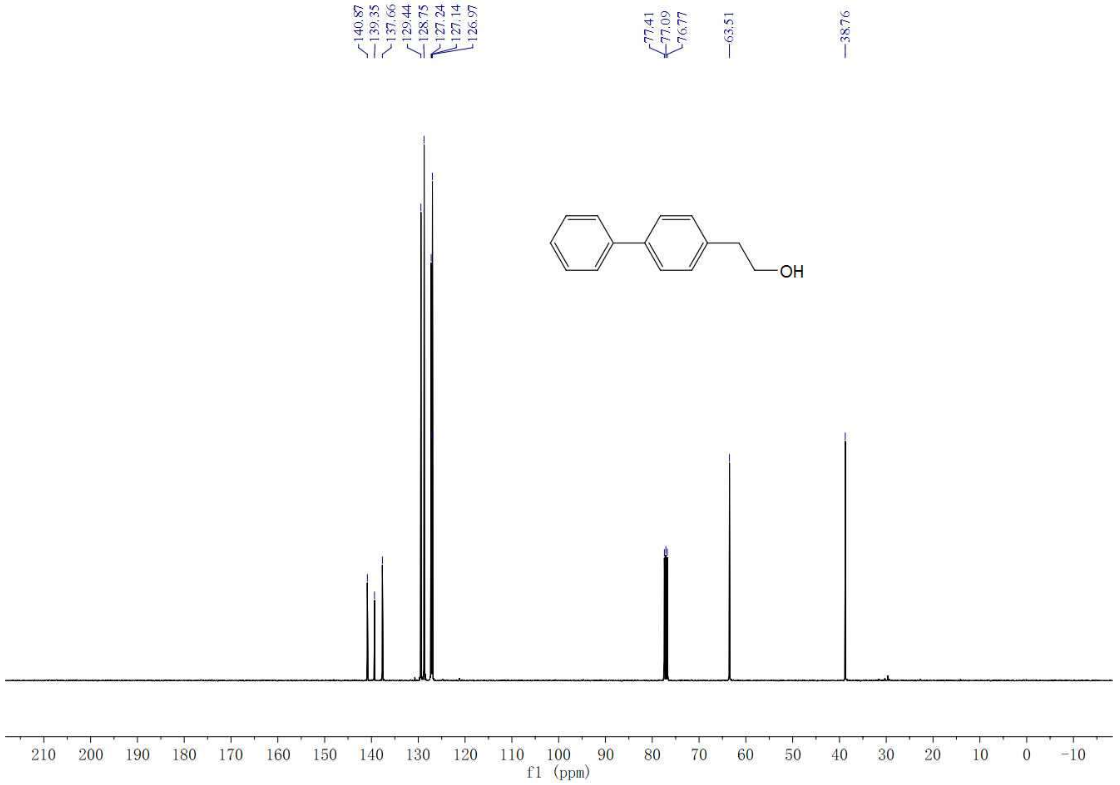

2-Acetylbiphenyl 3ma

^{1}H NMR

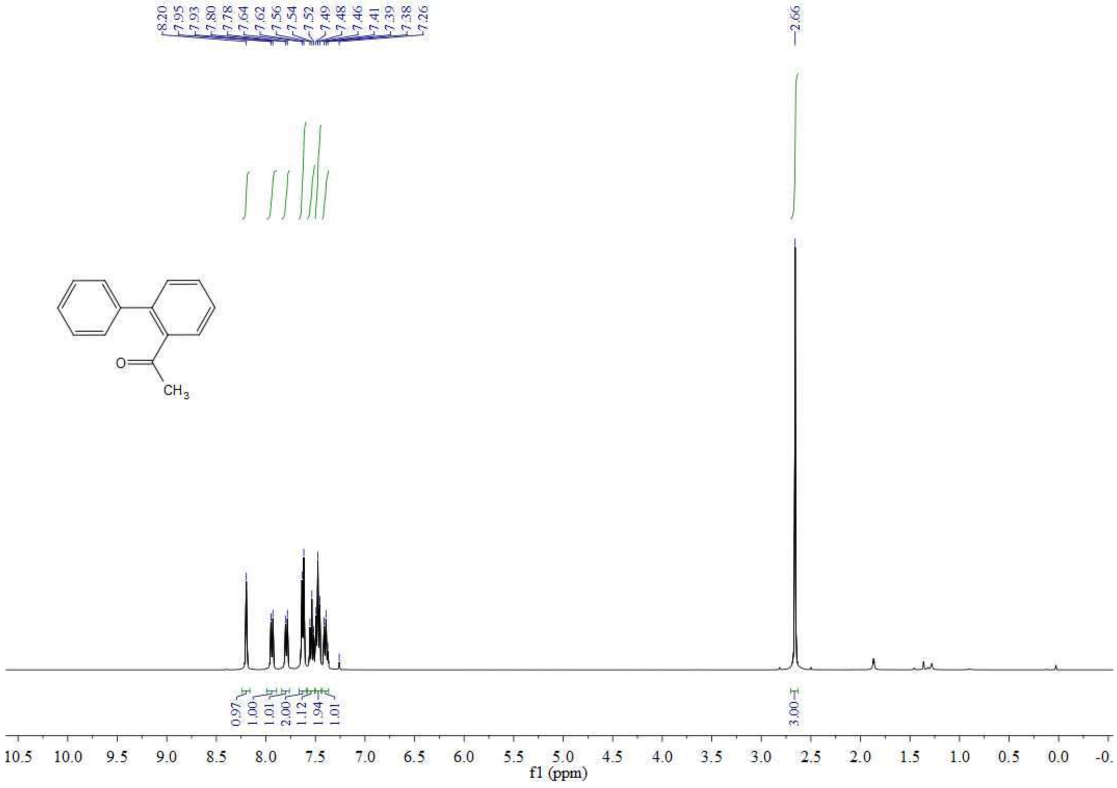

^{13}C NMR

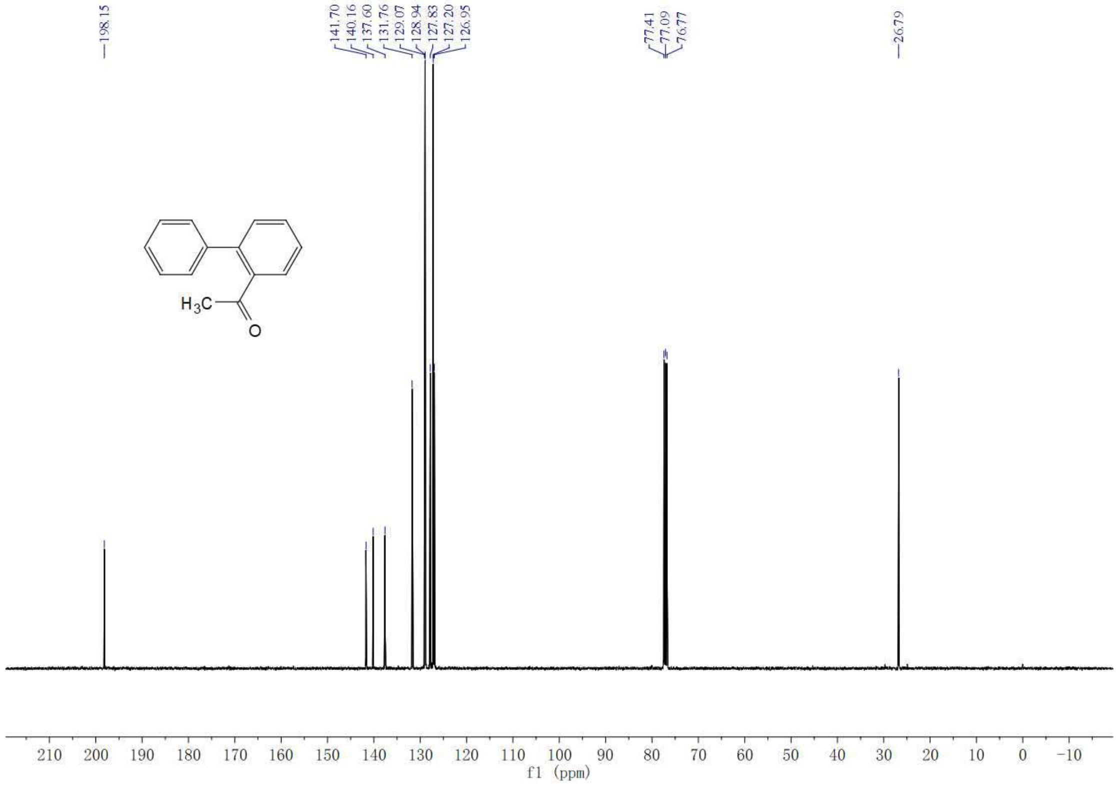

2-Aldehydebiphenyl 3na

^{1}H NMR

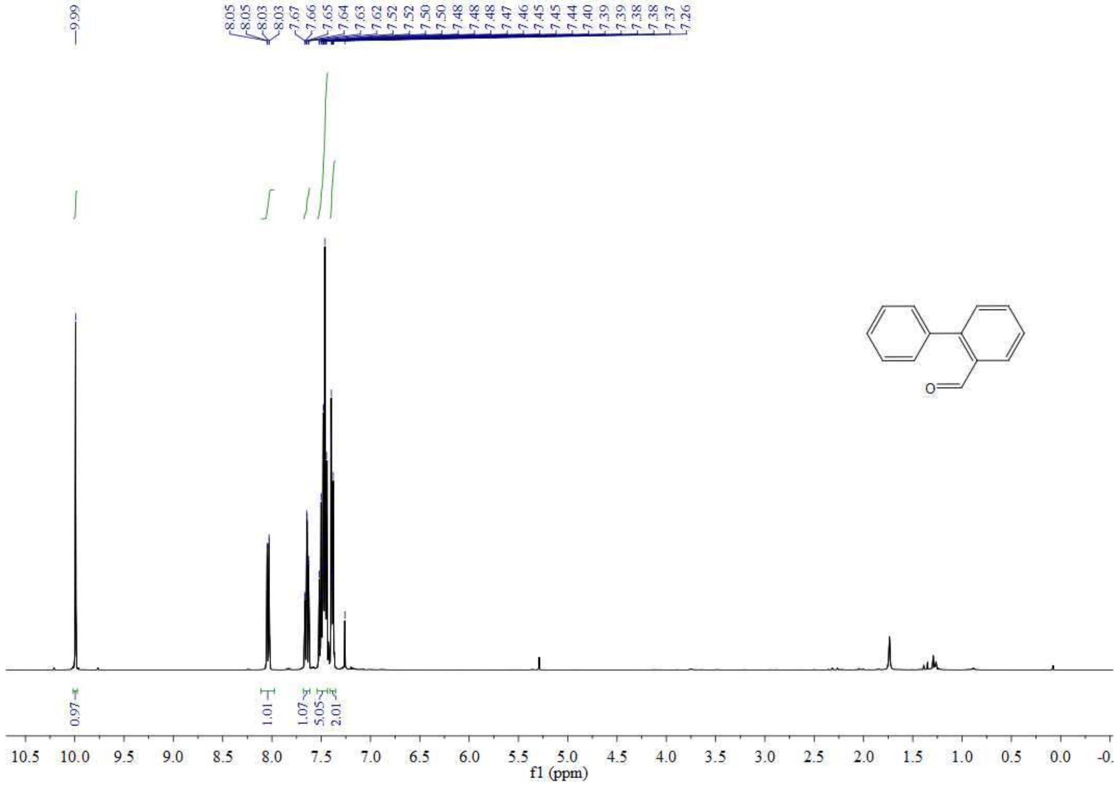

^{13}C NMR

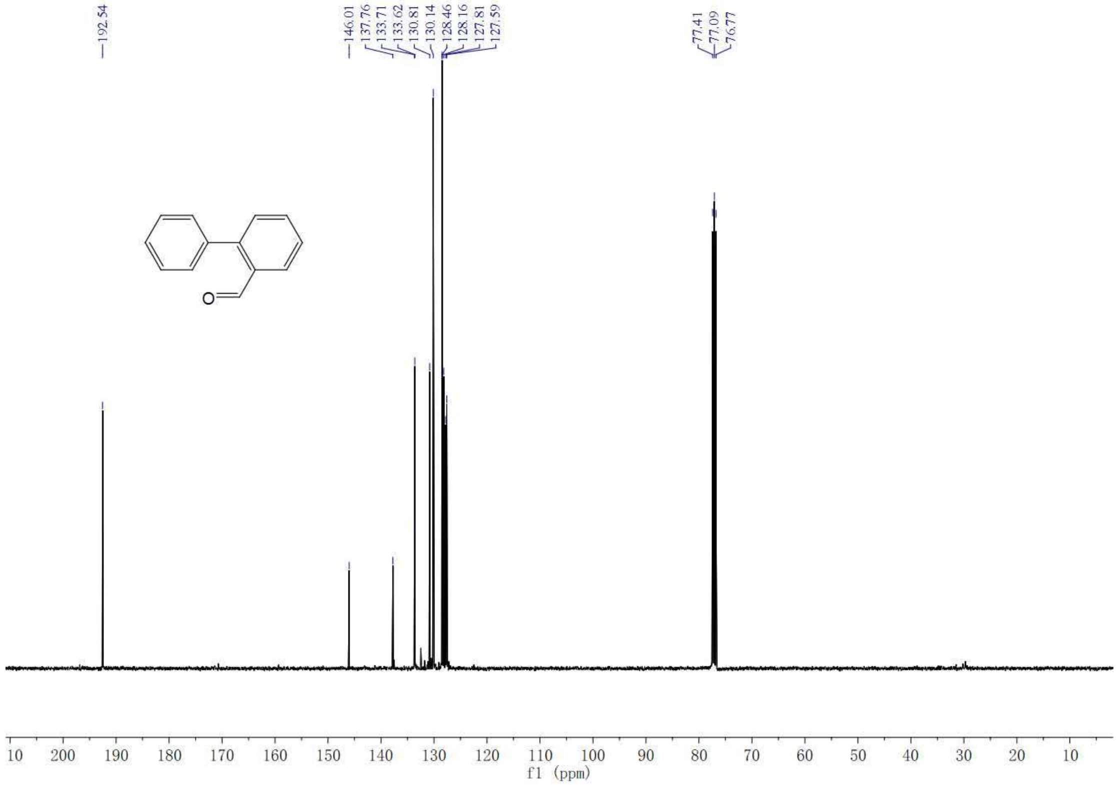

3-Acetylbiphenyl 3oa

^{1}H NMR

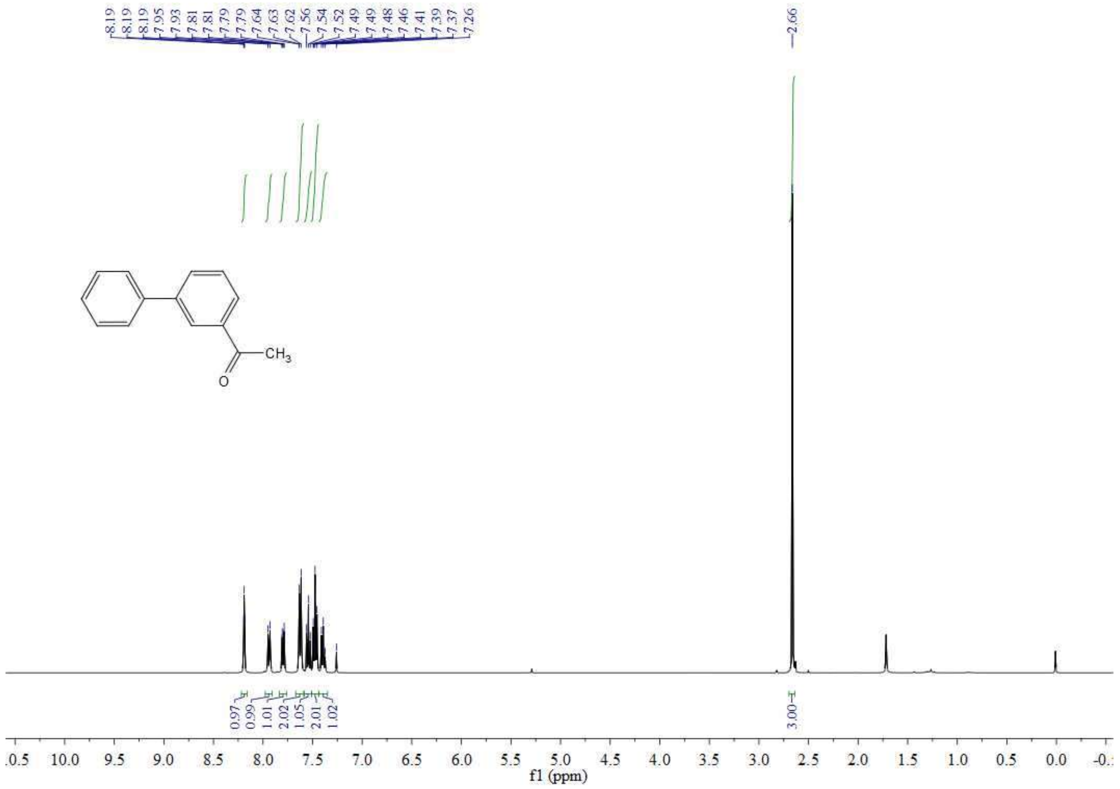

^{13}C NMR

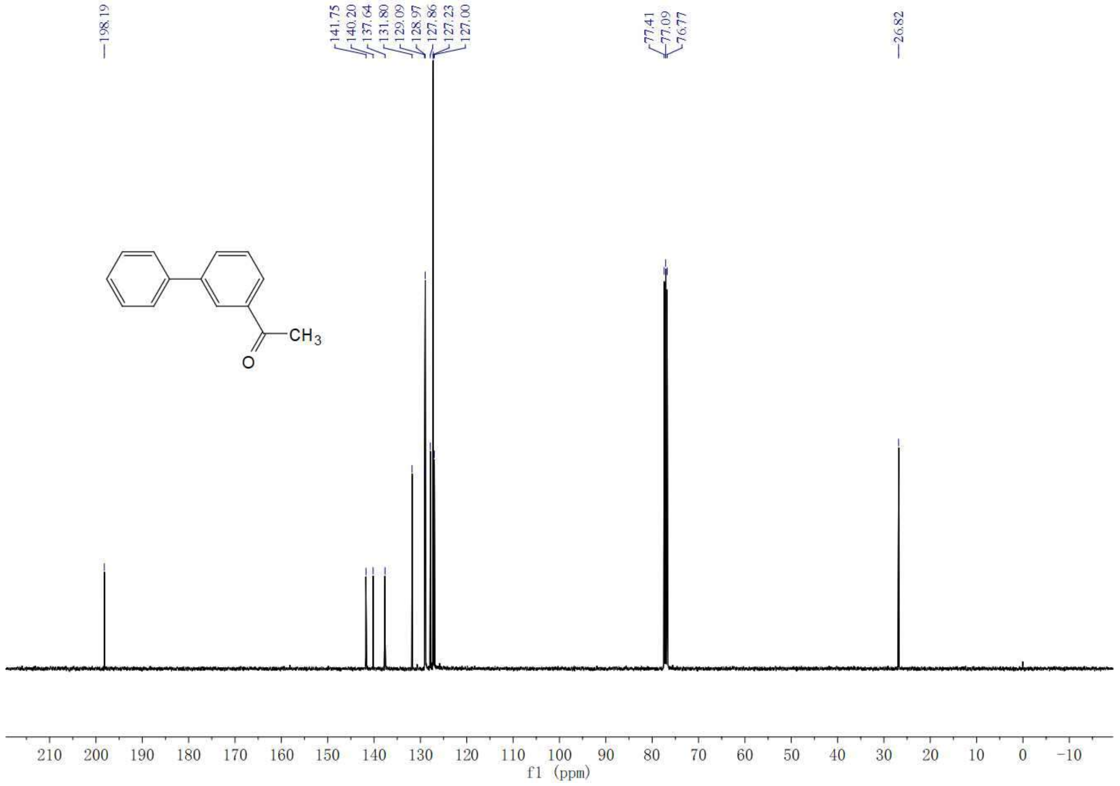

2-Tert-butylbiphenyl 3pa

^{1}H NMR

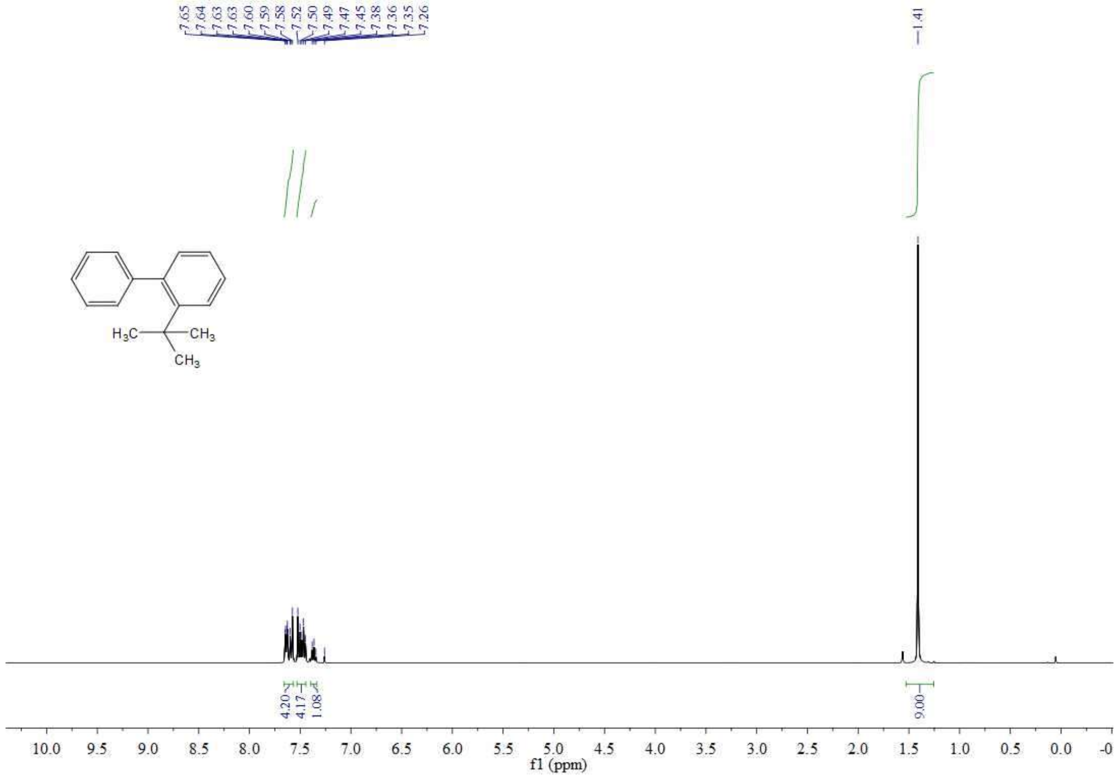

^{13}C NMR

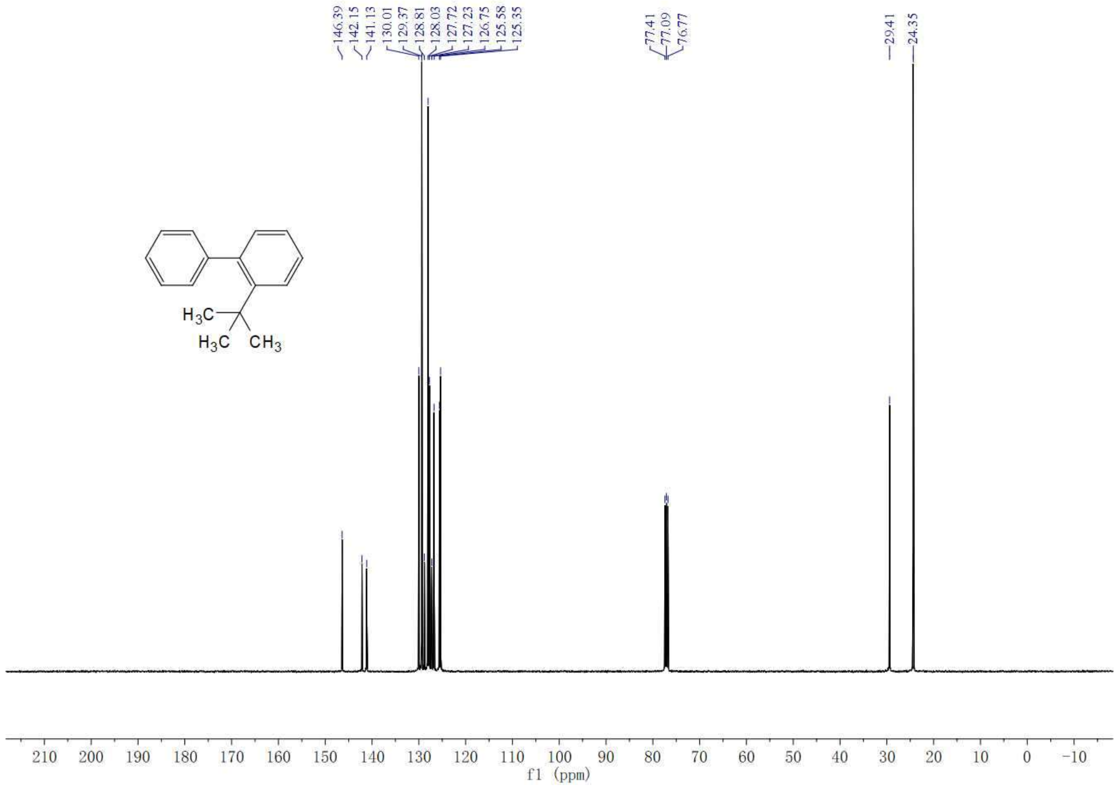

2-Methoxybiphenyl 3qa

^{1}H NMR

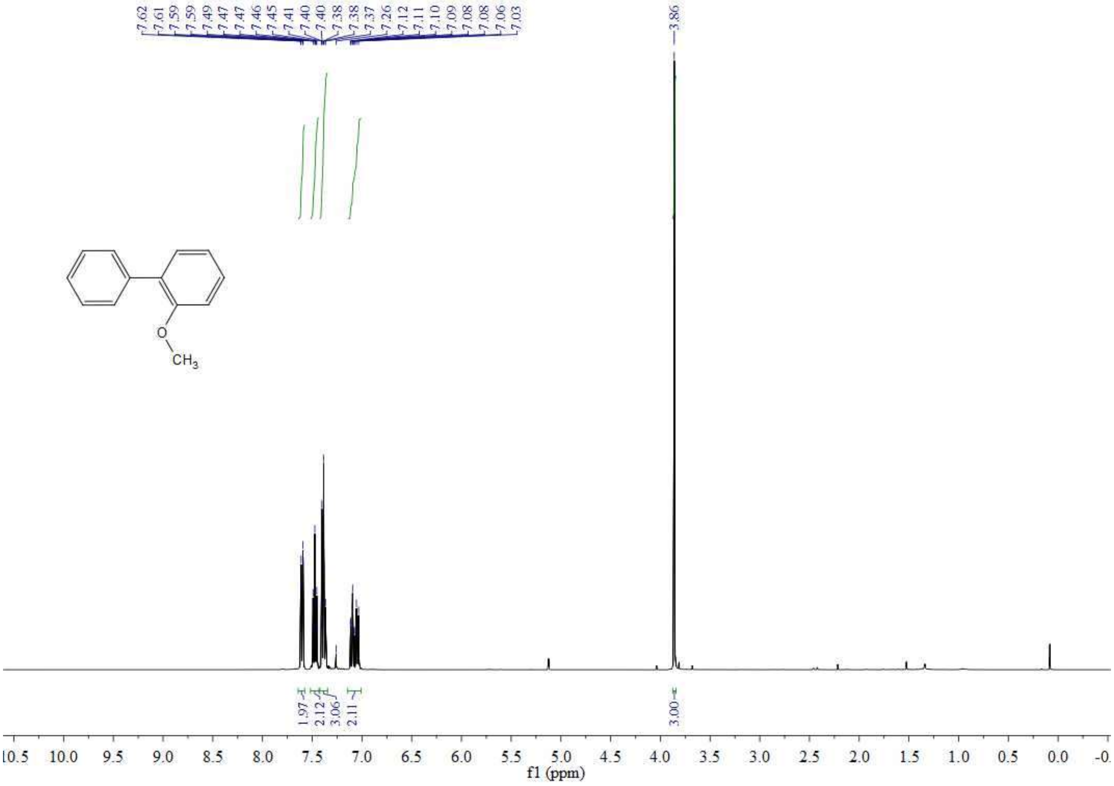

^{13}C NMR

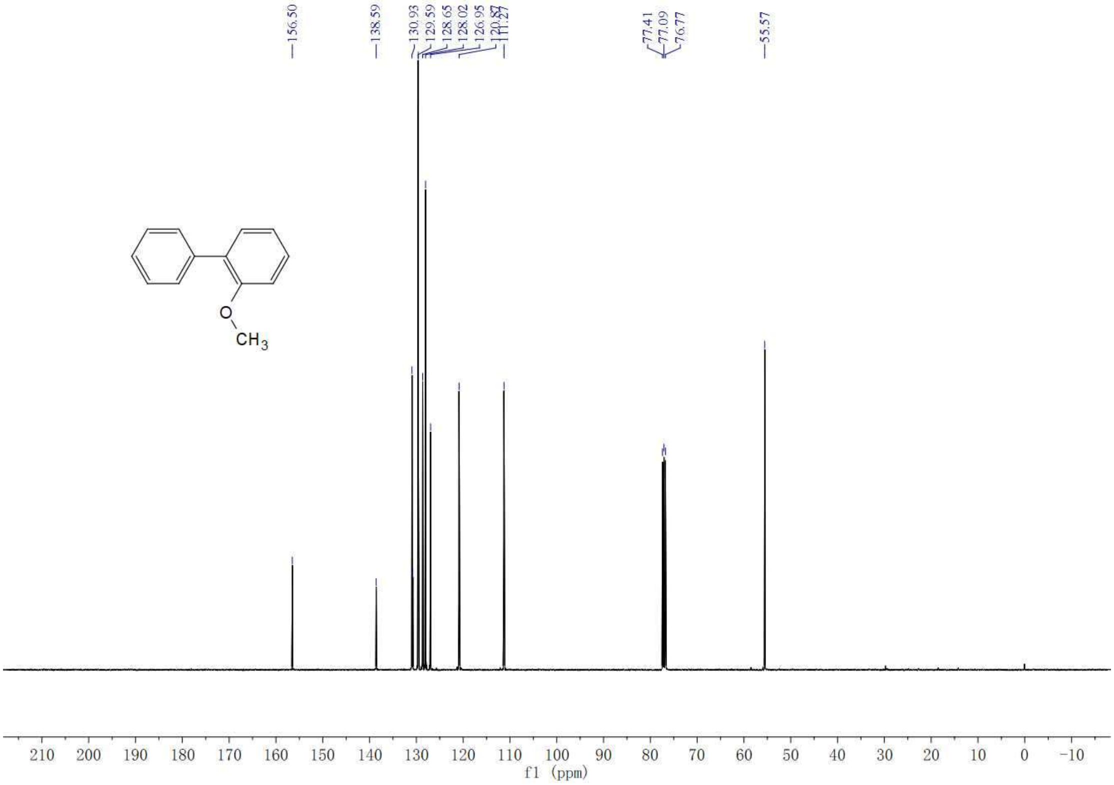

2-Methoxy-[1,1'-biphenyl]-4-carbaldehyde 3ra

^{1}H NMR

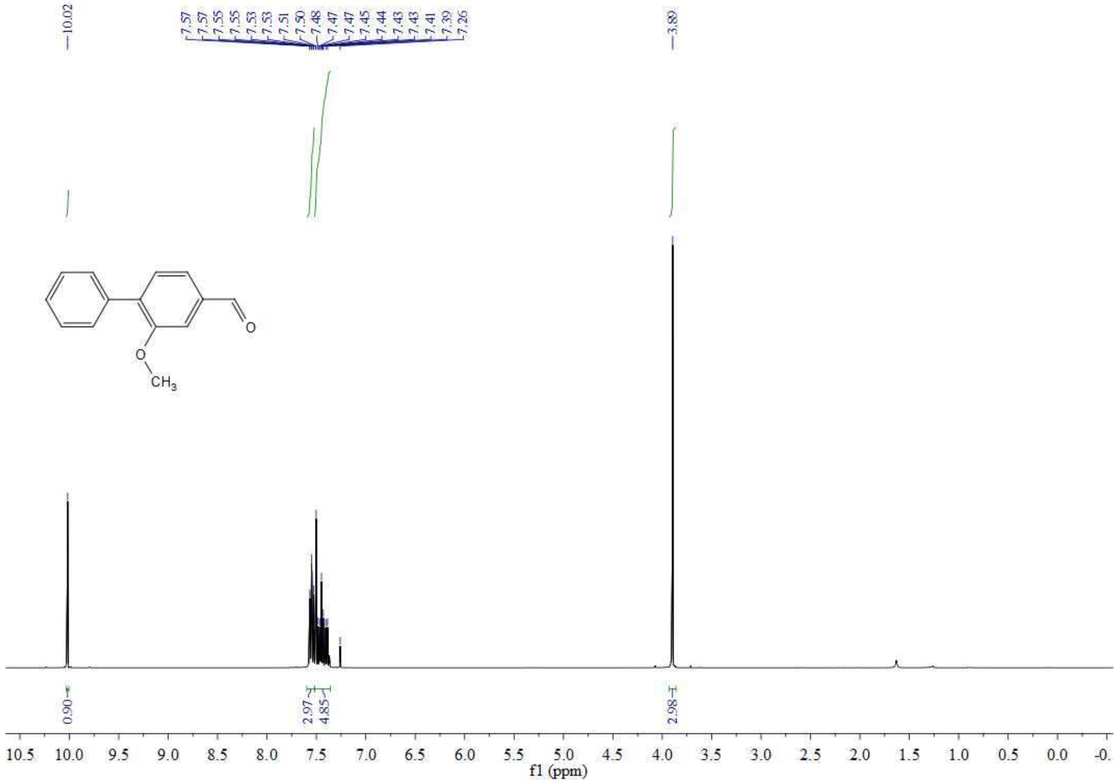

^{13}C NMR

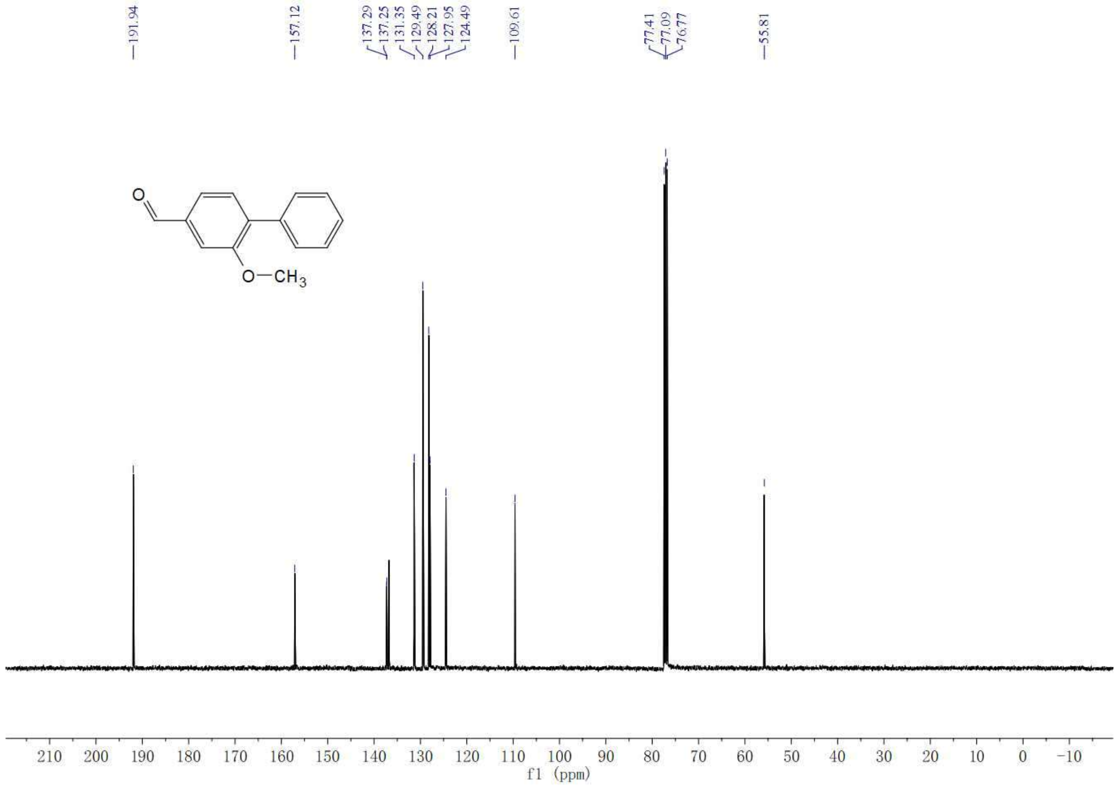

2-Tosyloxybiphenyl 3sa

^{1}H NMR

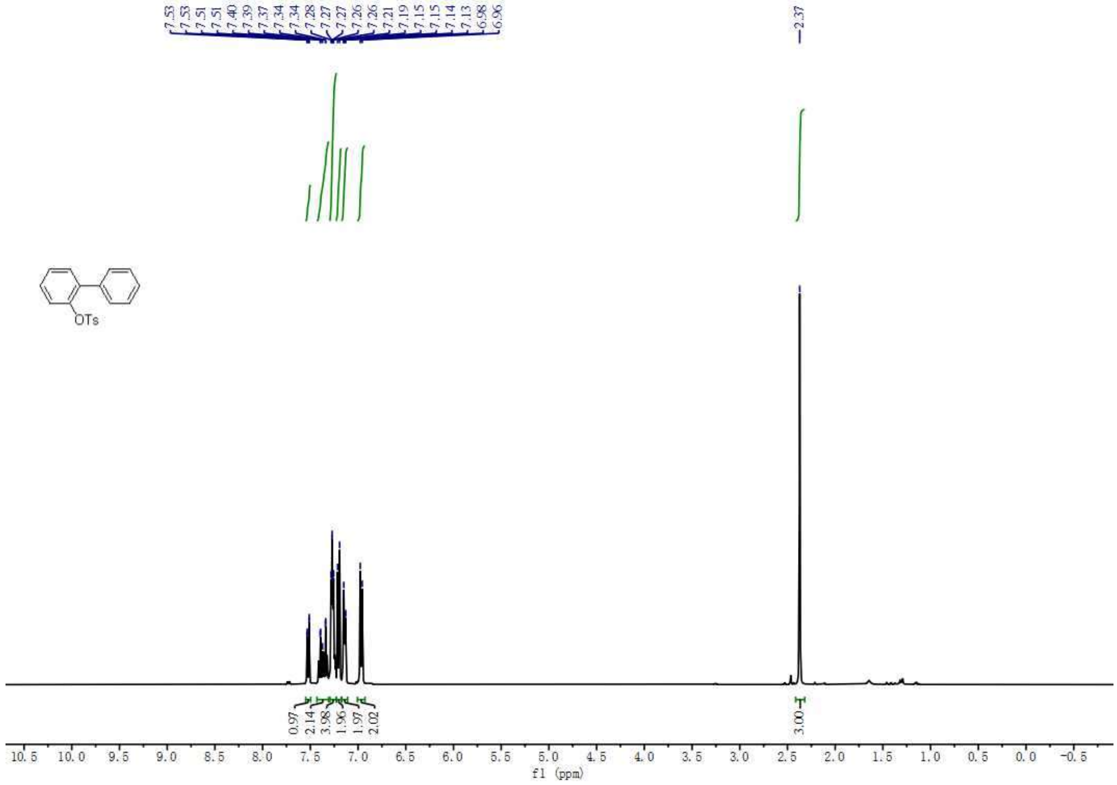

^{13}C NMR

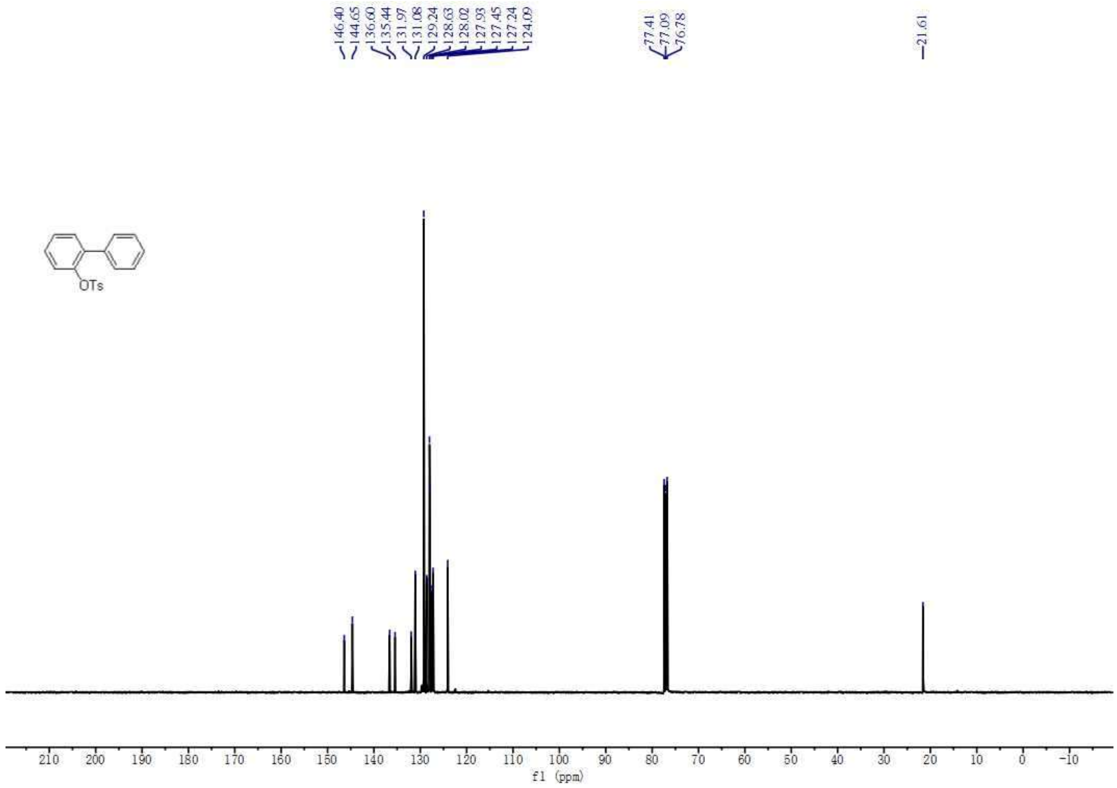

4-Phenylbiphenyl 3ta

^{1}H NMR

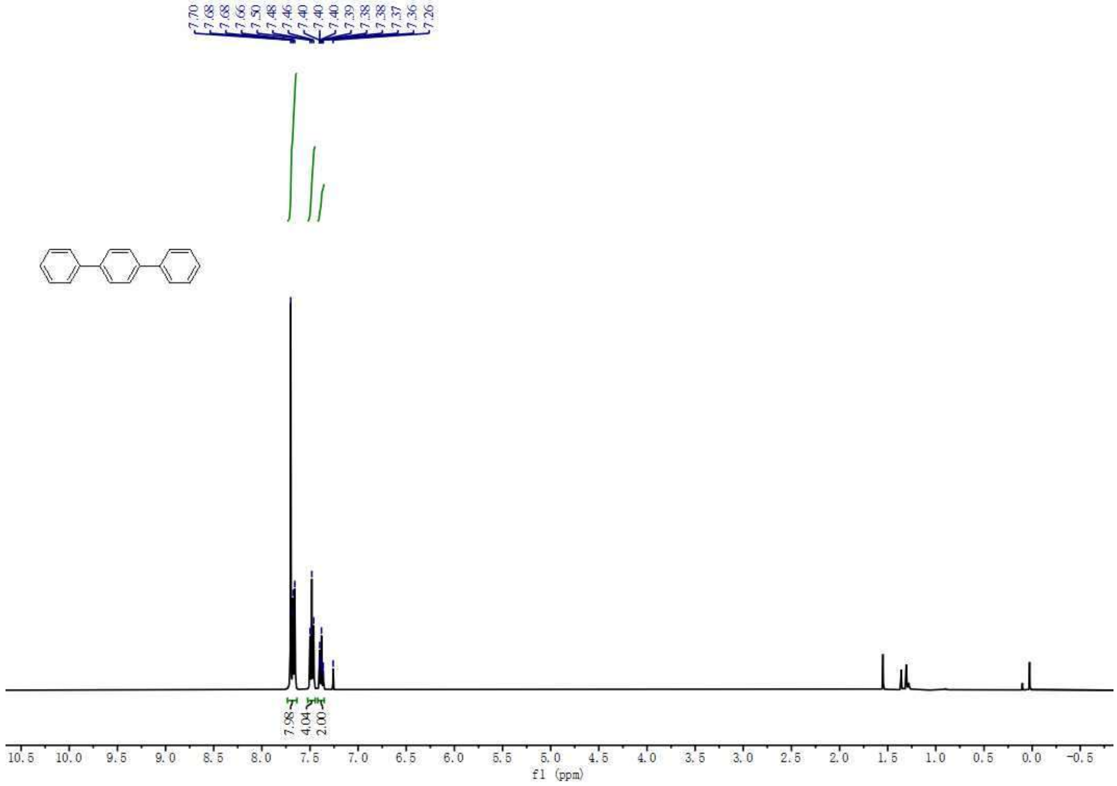

^{13}C NMR

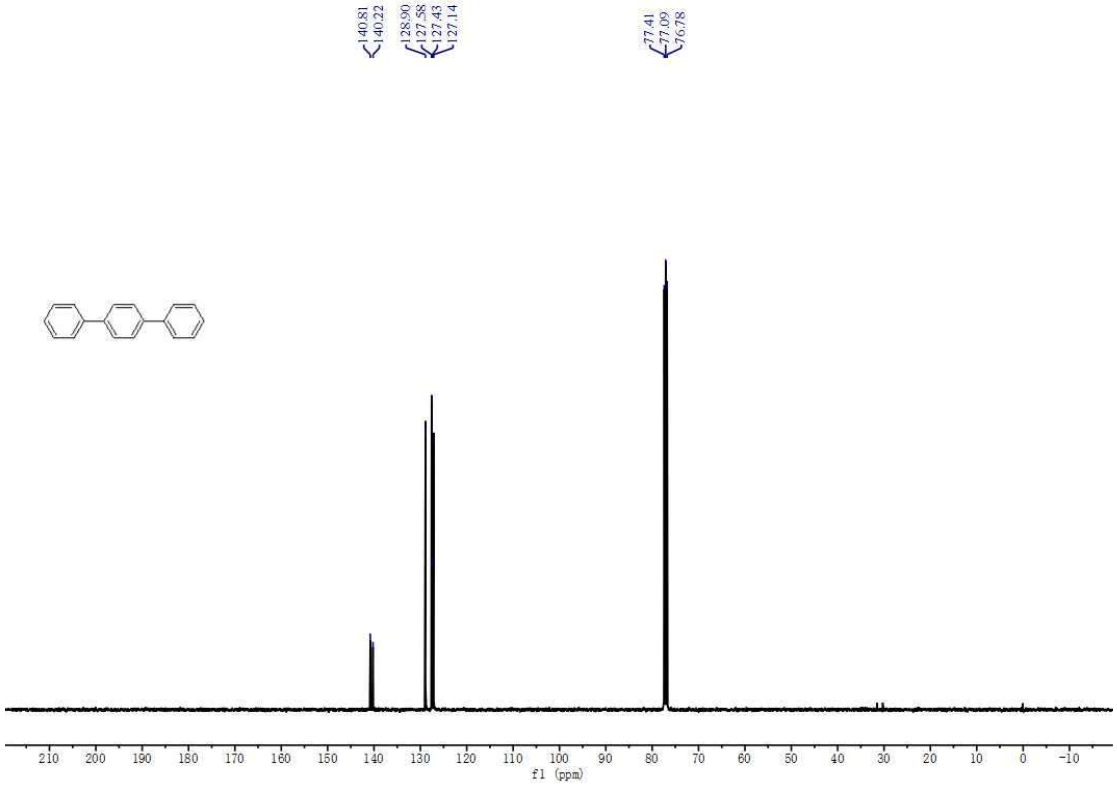

13-Methyl-3-phenyl-7,8,9,11,12,13,14,15,16,17-decahydro-6H-cyclopenta[a]phenanthren-17-ol 3ua

^{1}H NMR

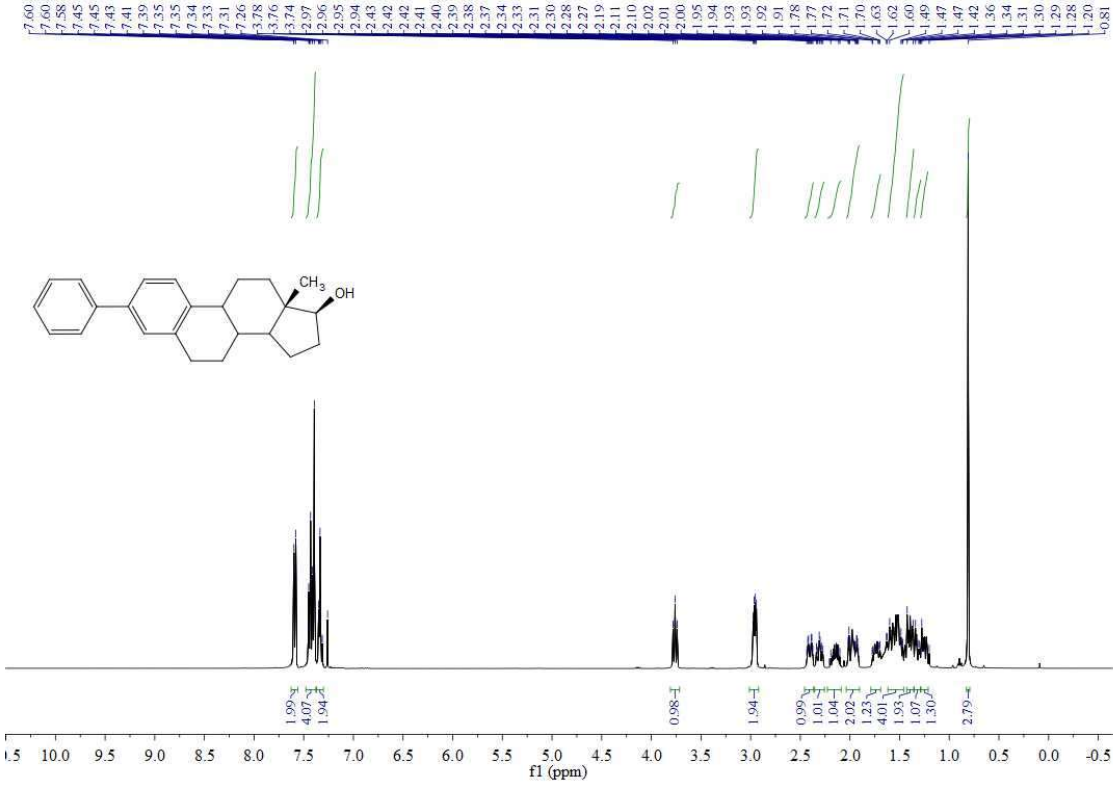

^{13}C NMR

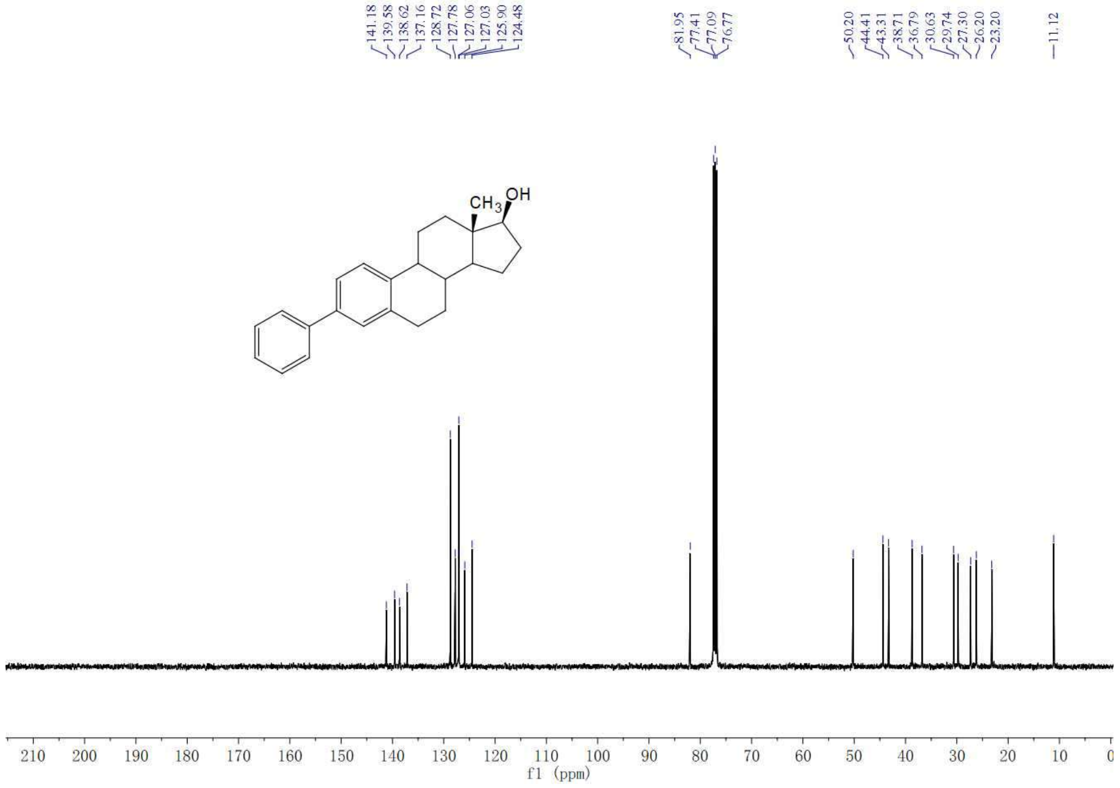

13-Methyl-3-phenyl-6,7,8,9,11,12,13,14,15,16-decahydro-17H-cyclopenta[a]phenanthren-17-one 3va

^{1}H NMR

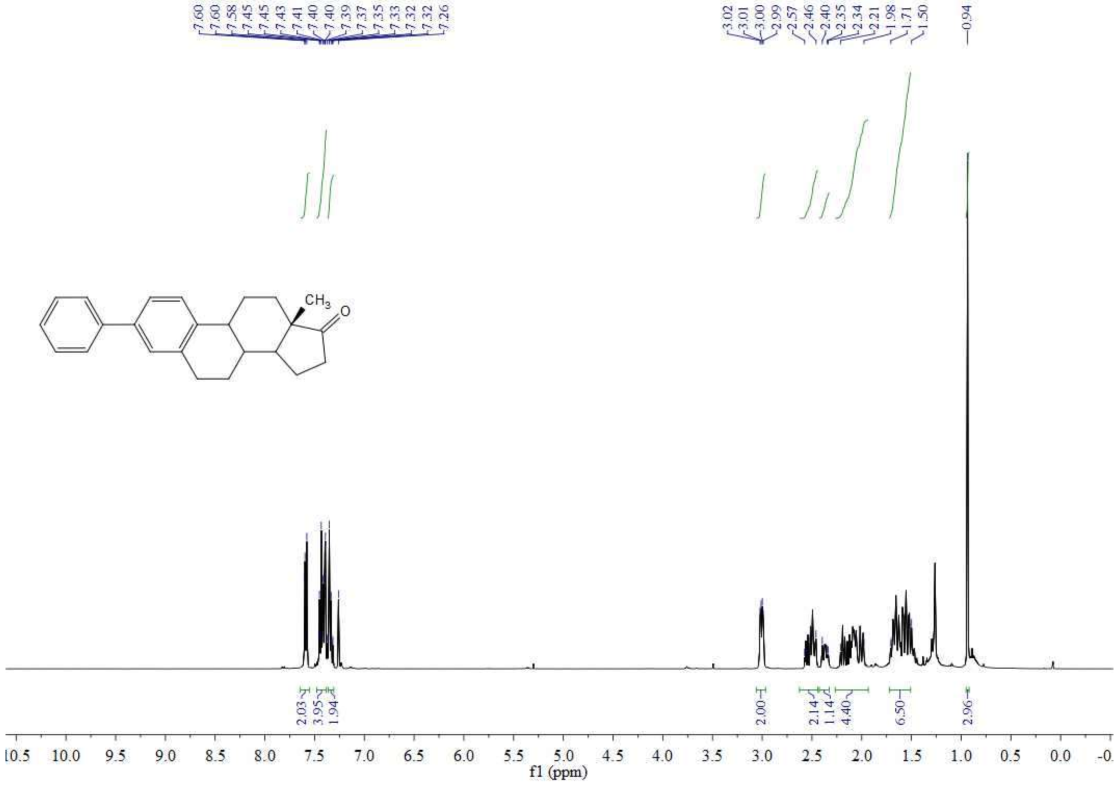

^{13}C NMR

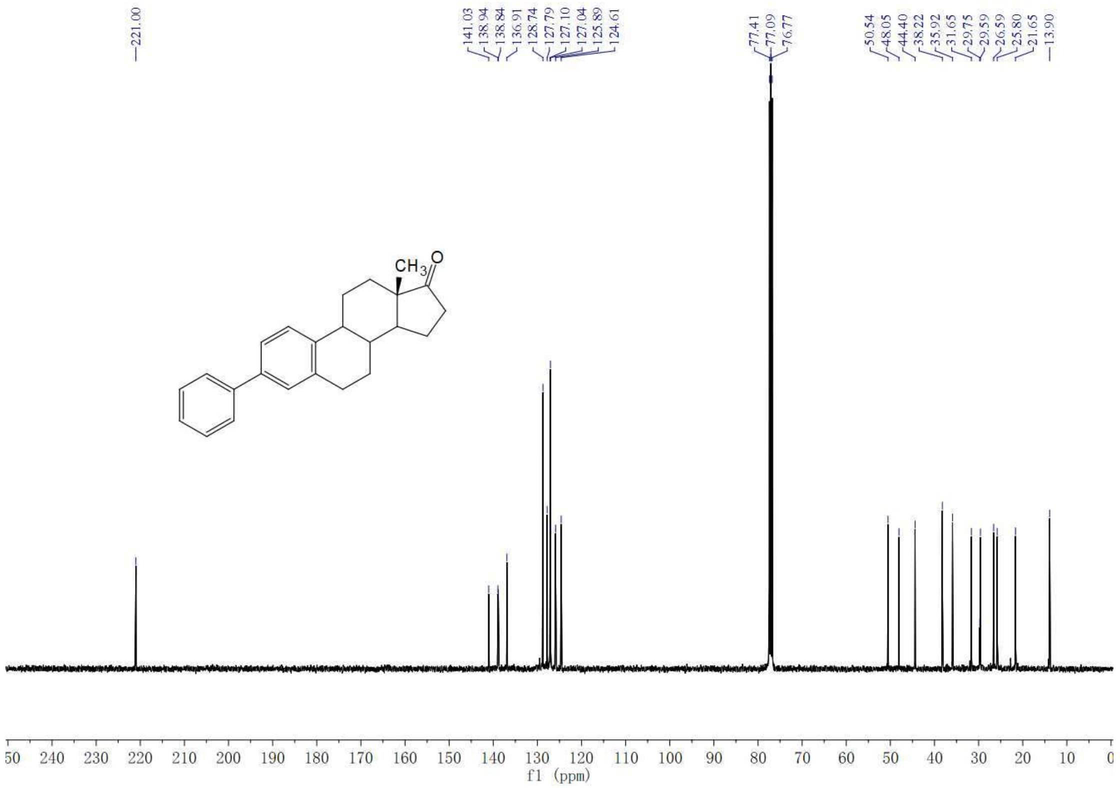

4-Acetyl-4'-methylbiphenyl 3ab

^{1}H NMR

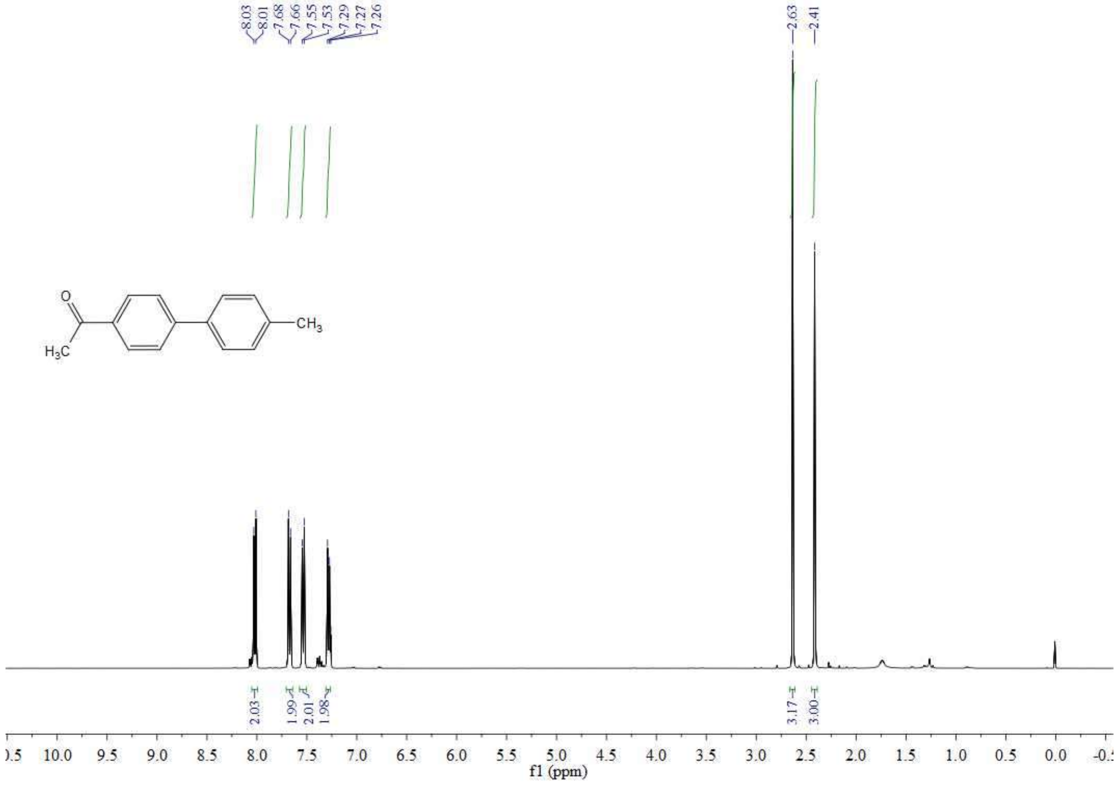

^{13}C NMR

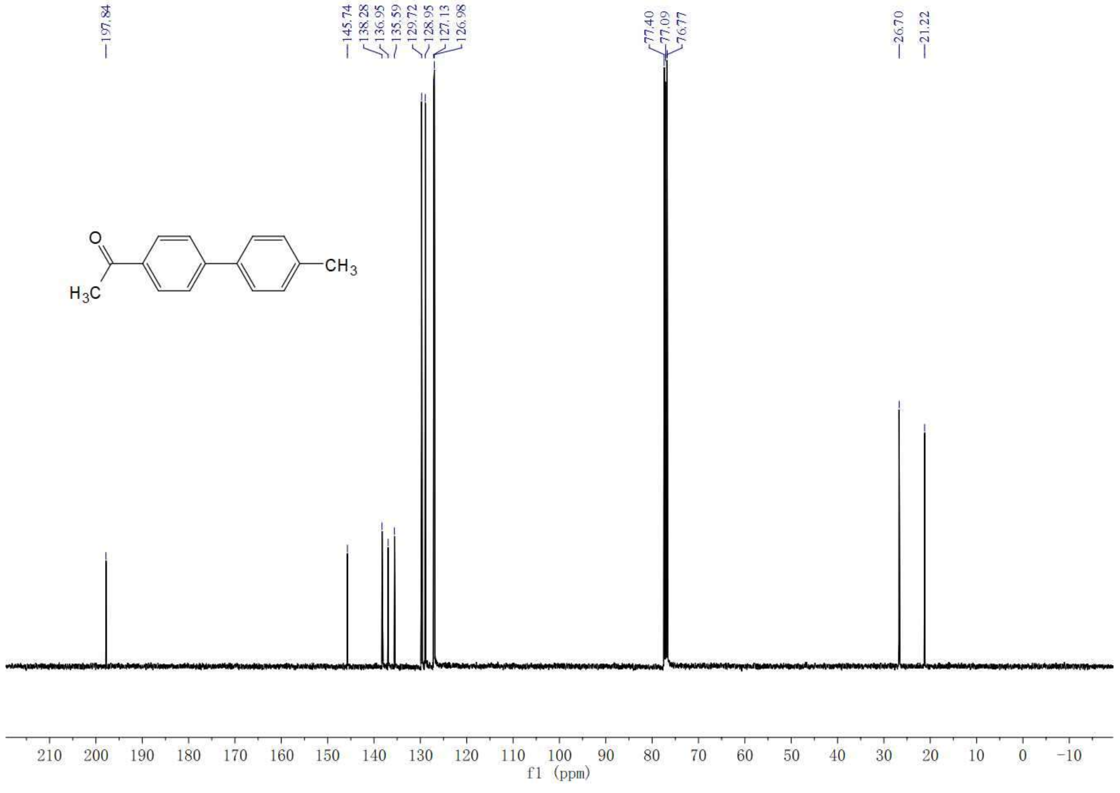

4-Acetyl-4'-tertiarybutylbiphenyl 3ac

^{1}H NMR

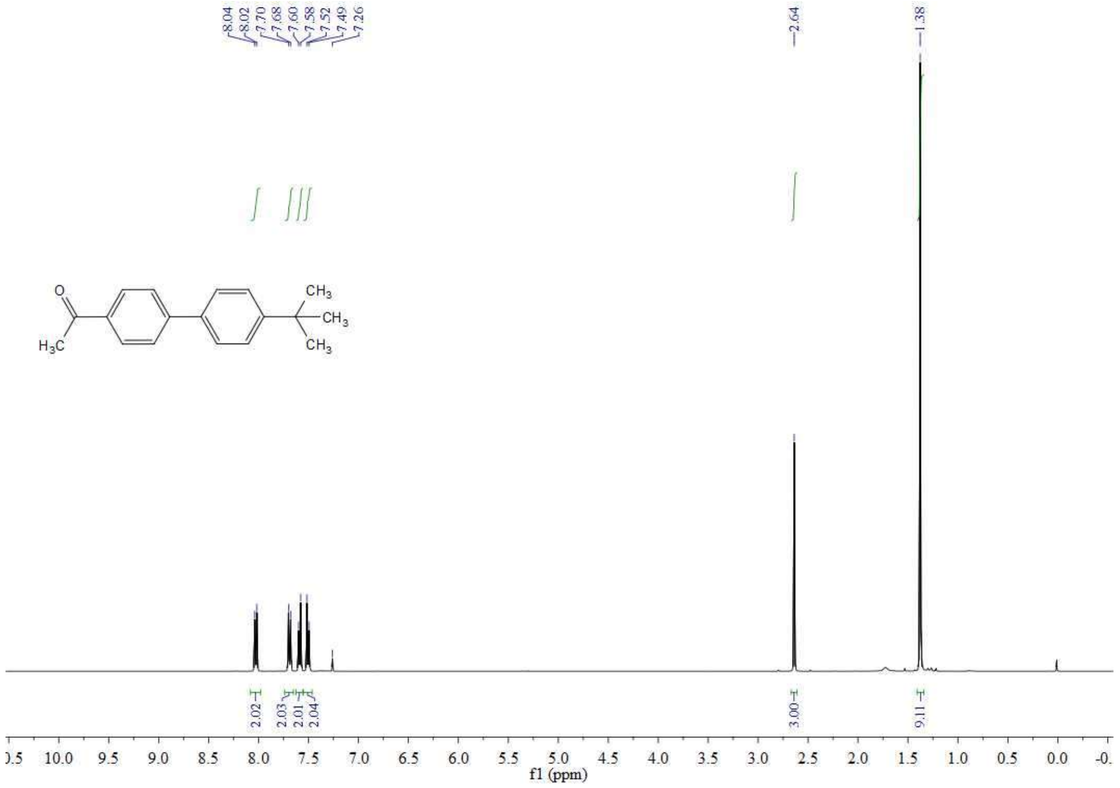

^{13}C NMR

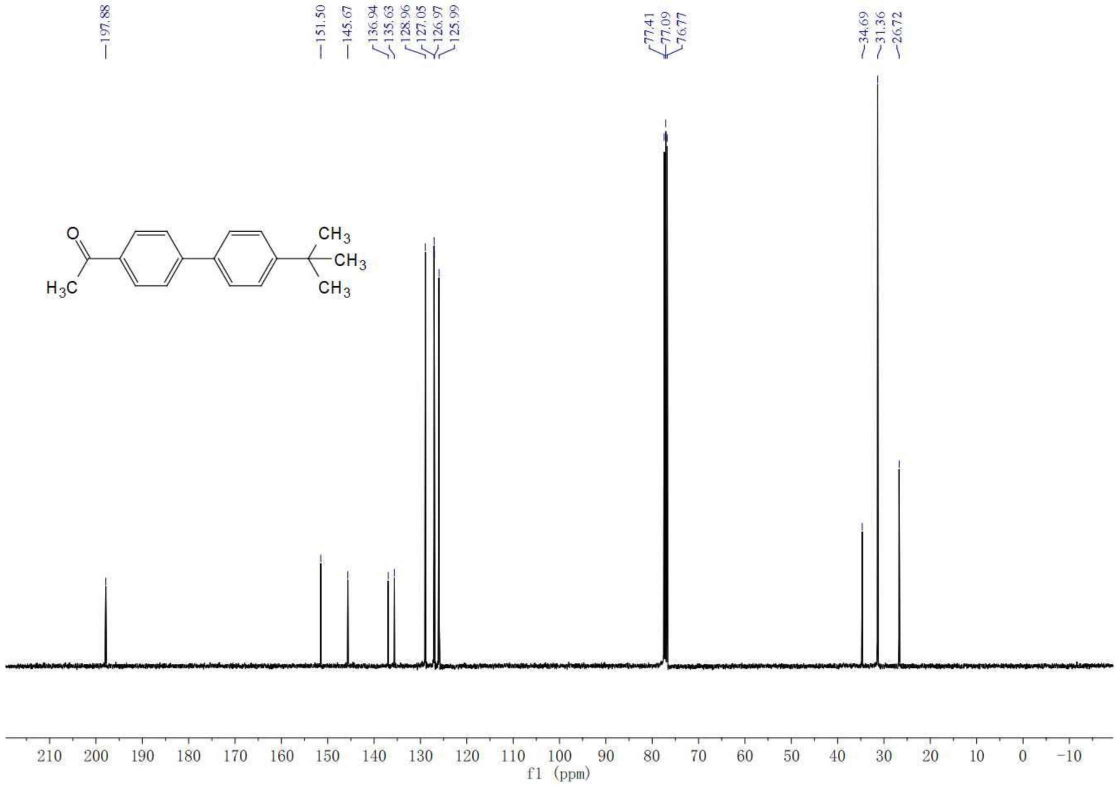

4-Acetyl-4'-methoxybiphenyl 3ad

^{1}H NMR

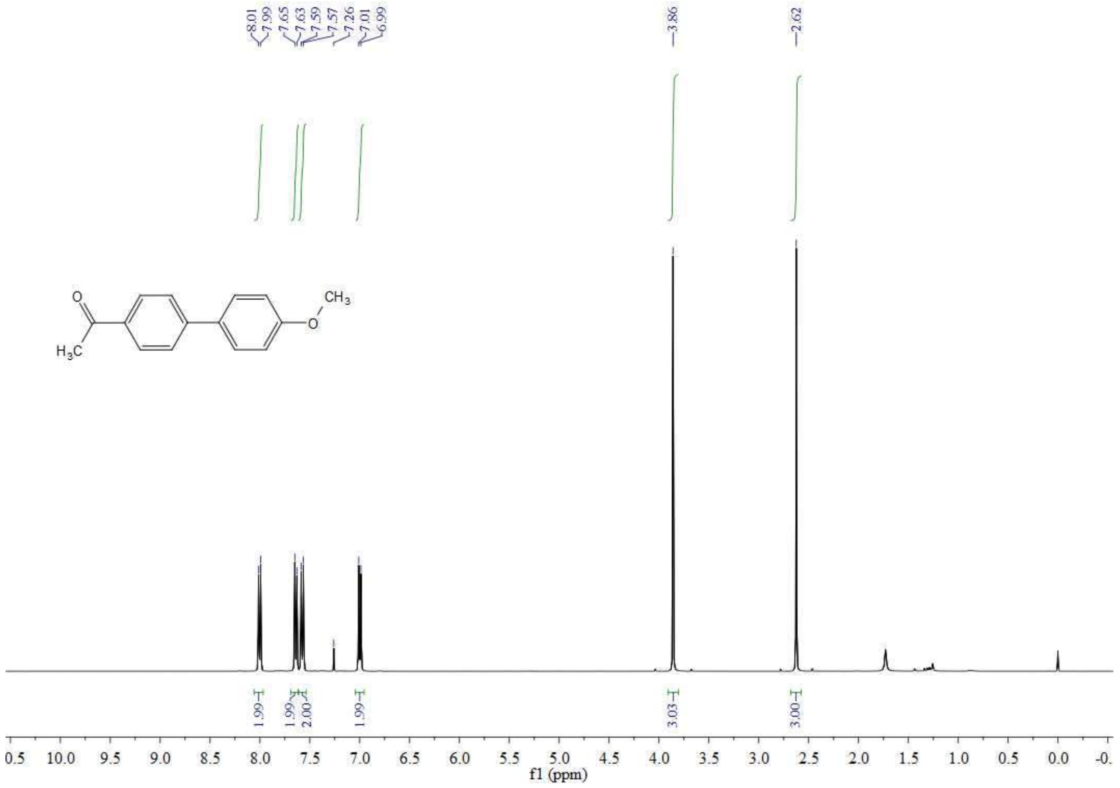

^{13}C NMR

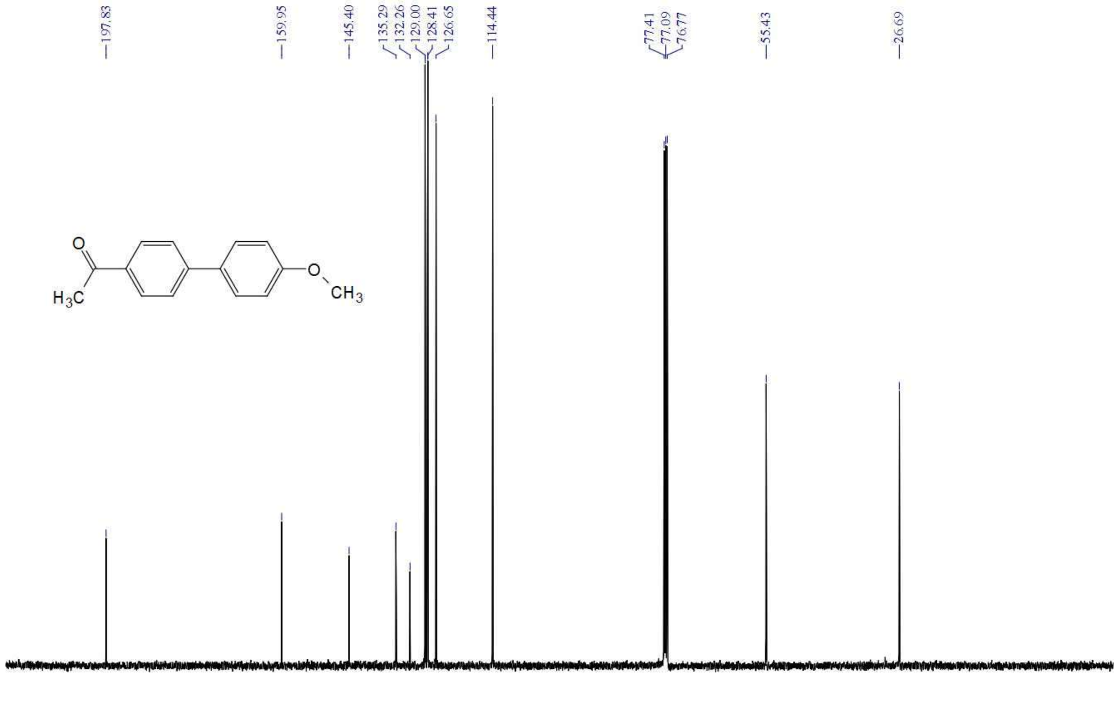

4-Acetyl-4'-fluorobiphenyl 3ae

^{1}H NMR

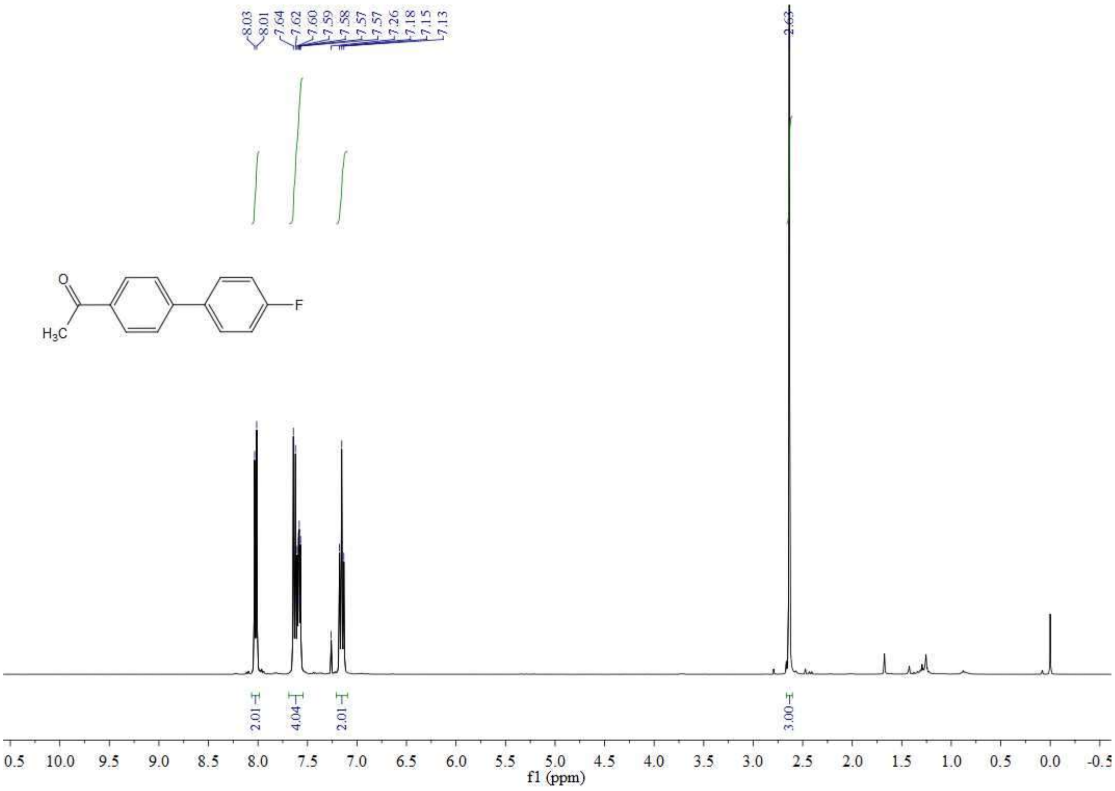

^{13}C NMR

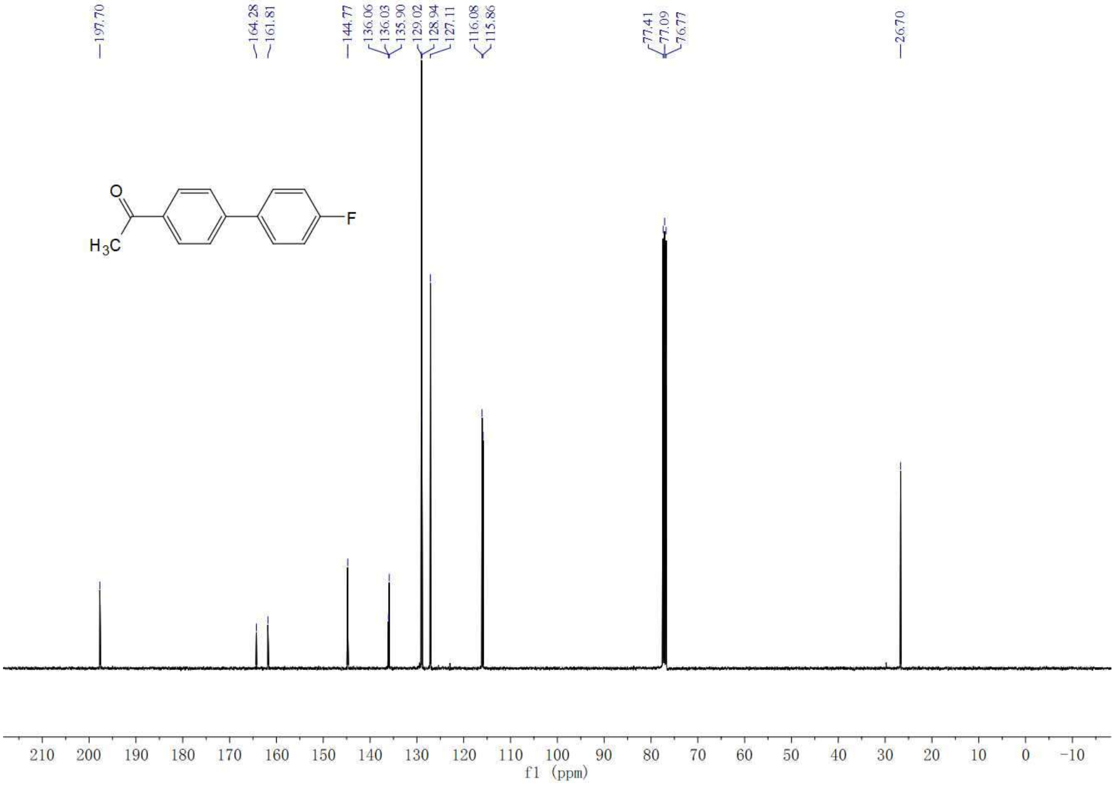

4-Acetyl-4'-trifluoromethylbiphenyl 3af

^{1}H NMR

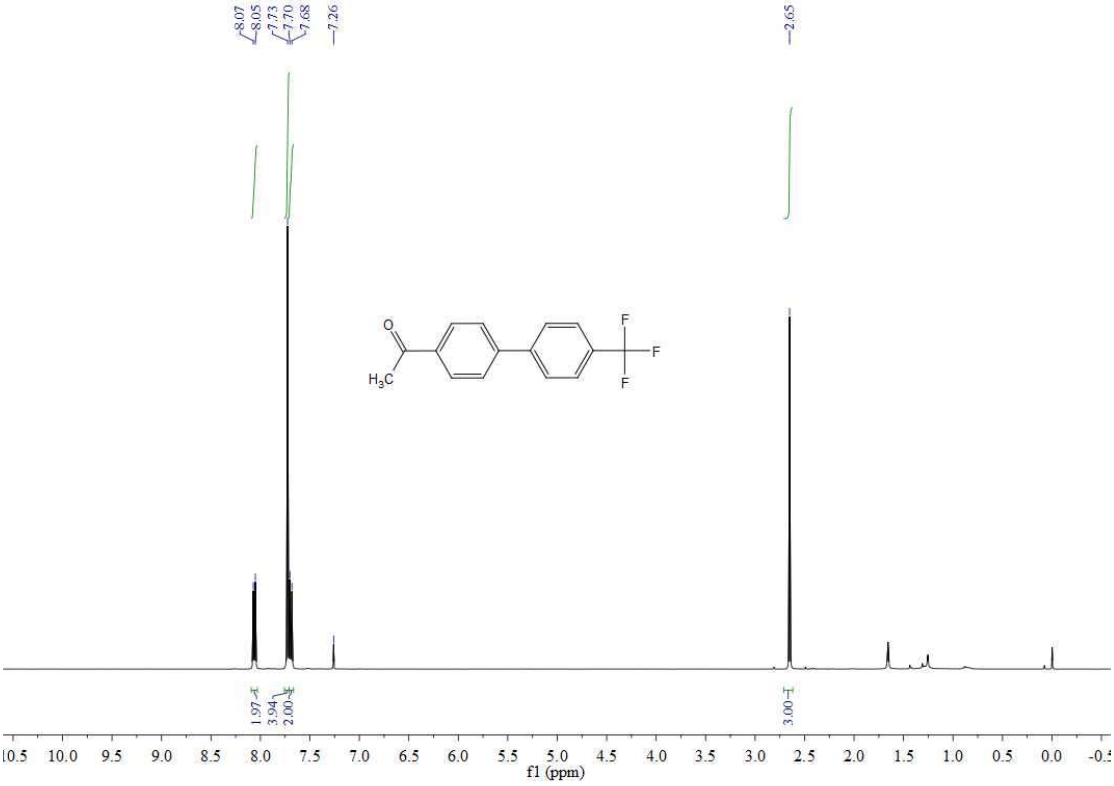

^{13}C NMR

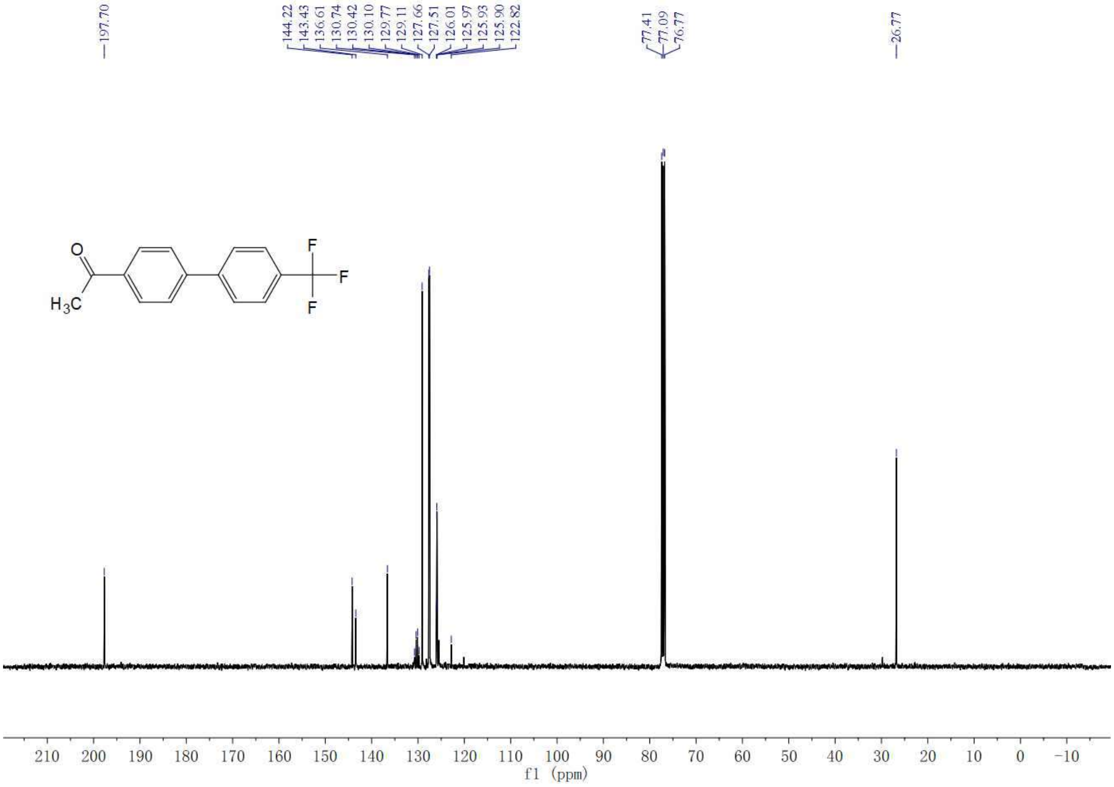

4-Acetyl-4'-formylbiphenyl 3ag

^{1}H NMR

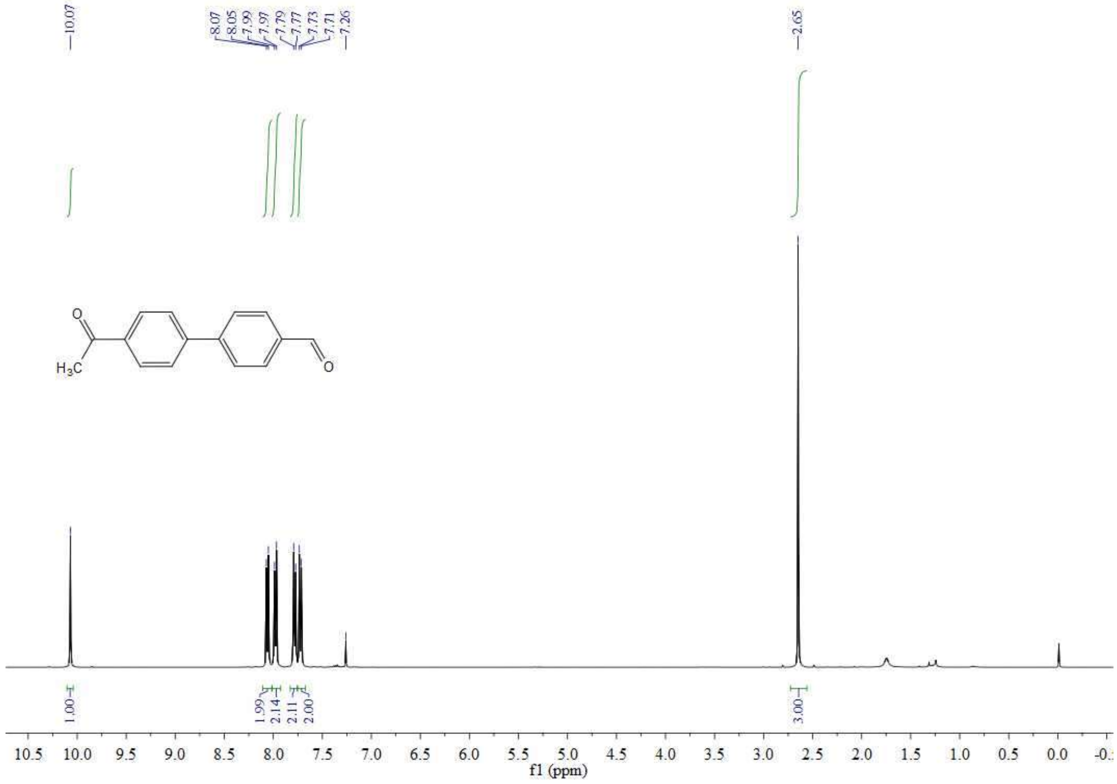

^{13}C NMR

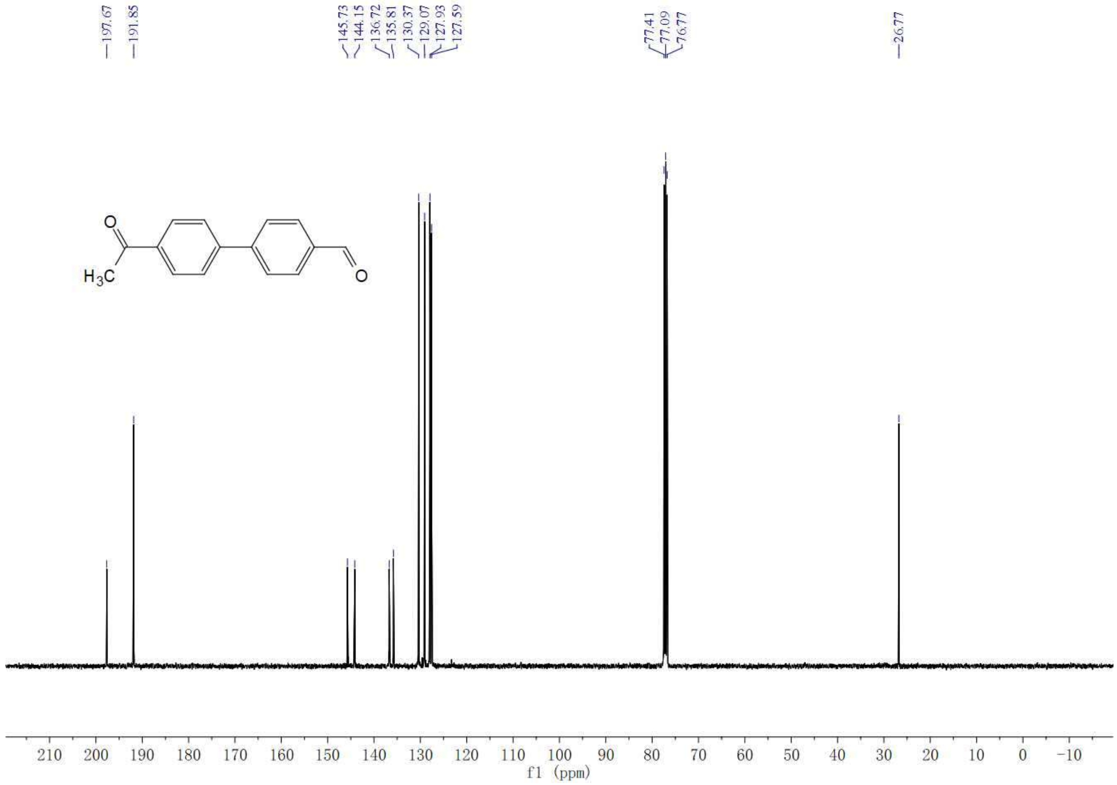

4-Acetyl-2'-ethylbiphenyl 3ah

^{1}H NMR

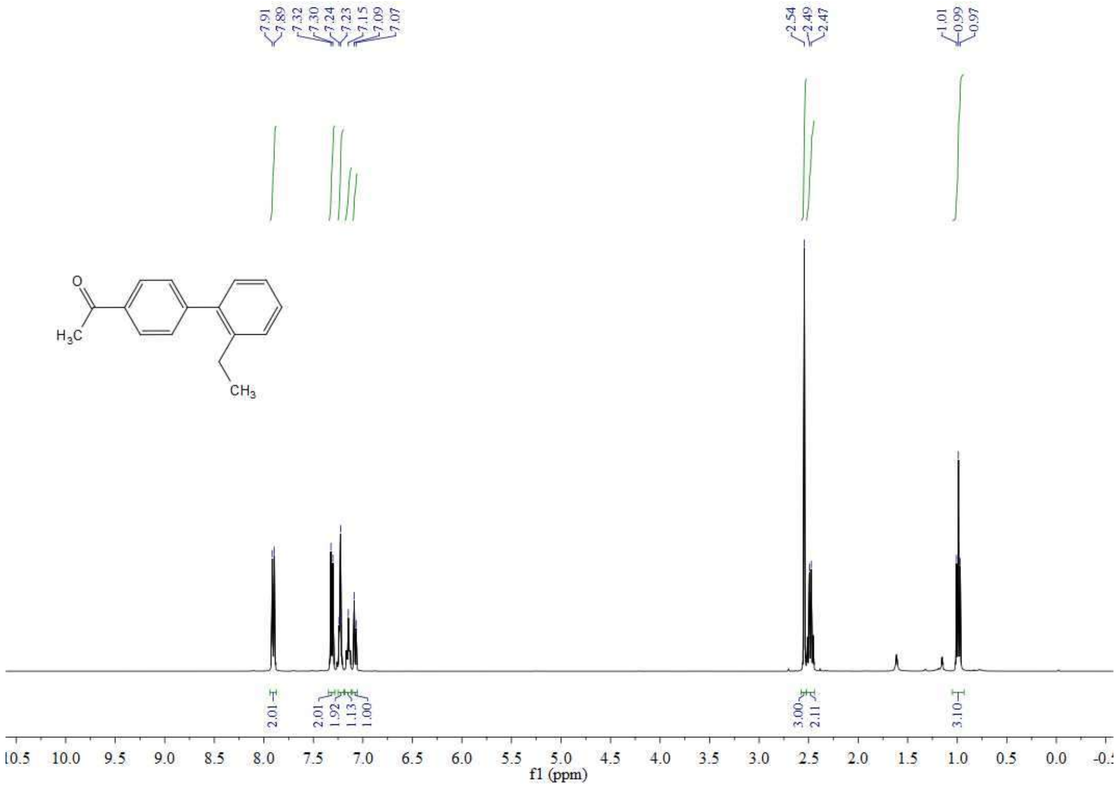

^{13}C NMR

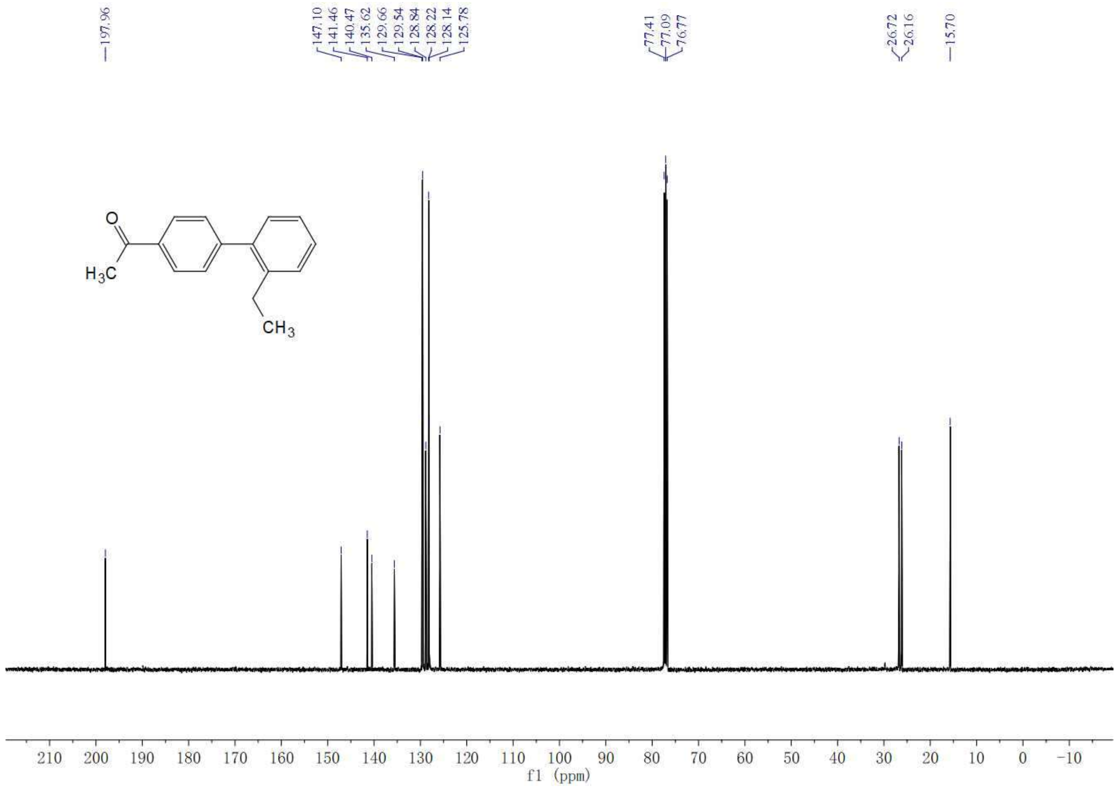

4-Acetyl-2'-isopropylbiphenyl 3ai

^{1}H NMR

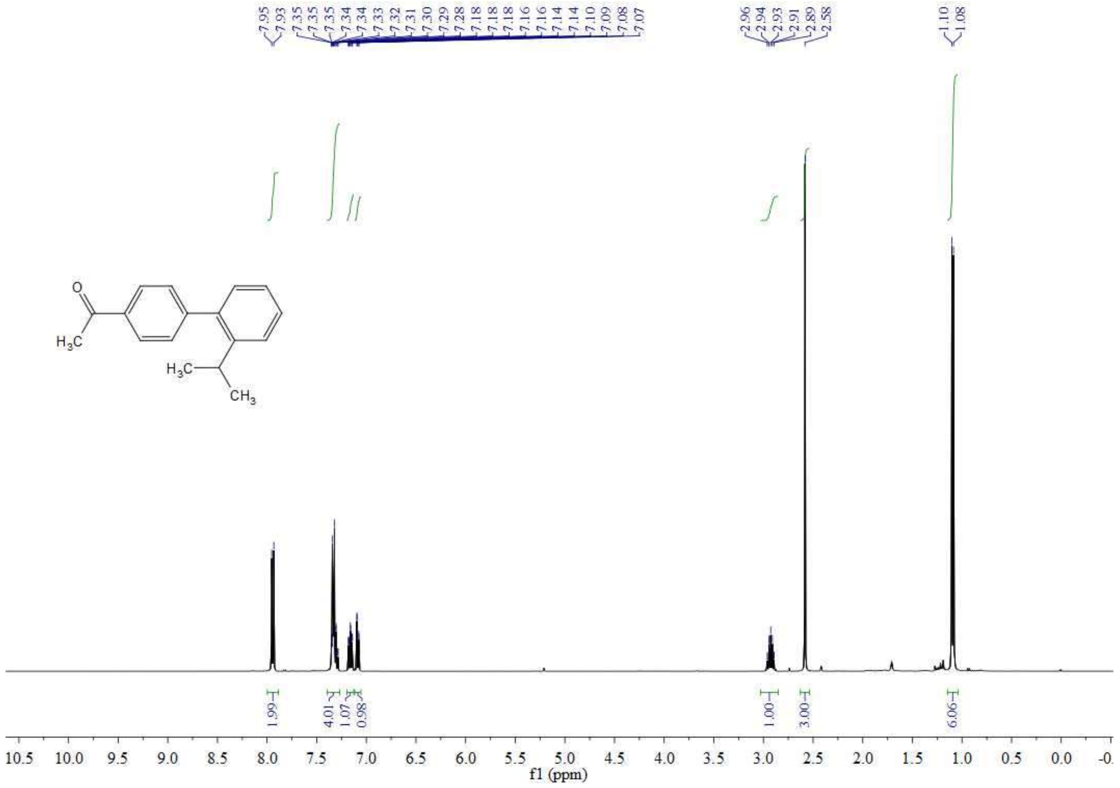

^{13}C NMR

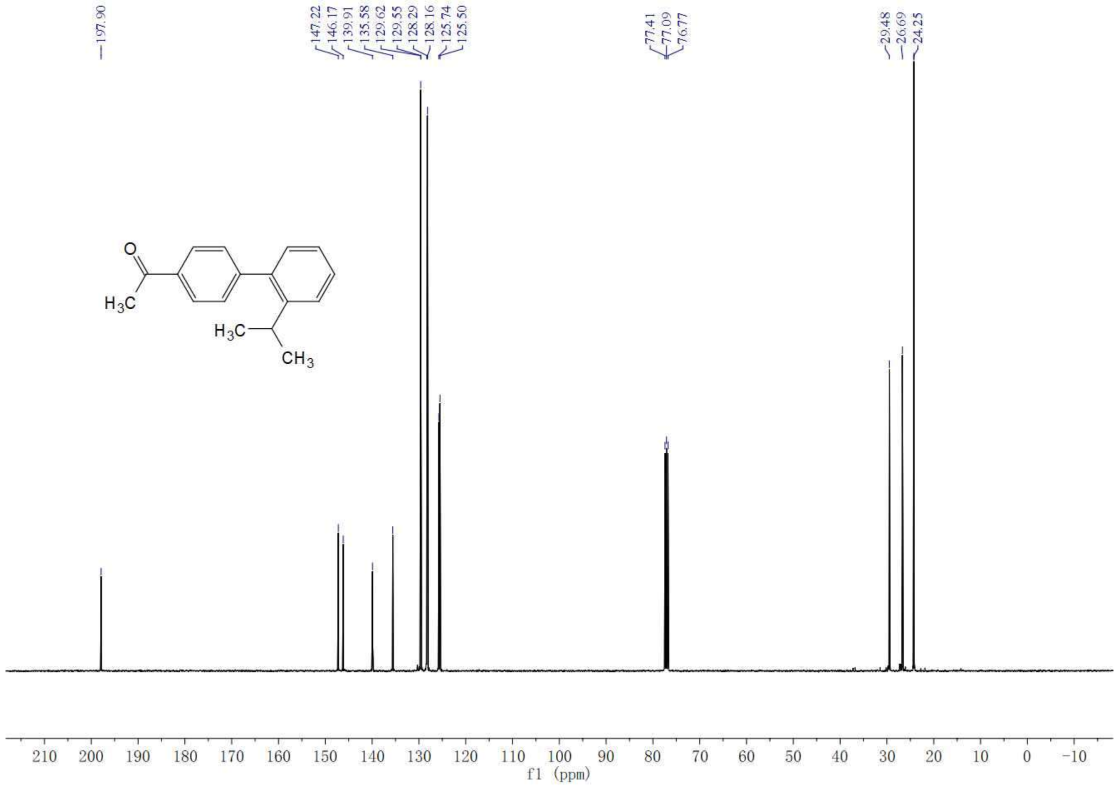

4-Acetylphenyl-2-phenylbenzene 3aj

^{1}H NMR

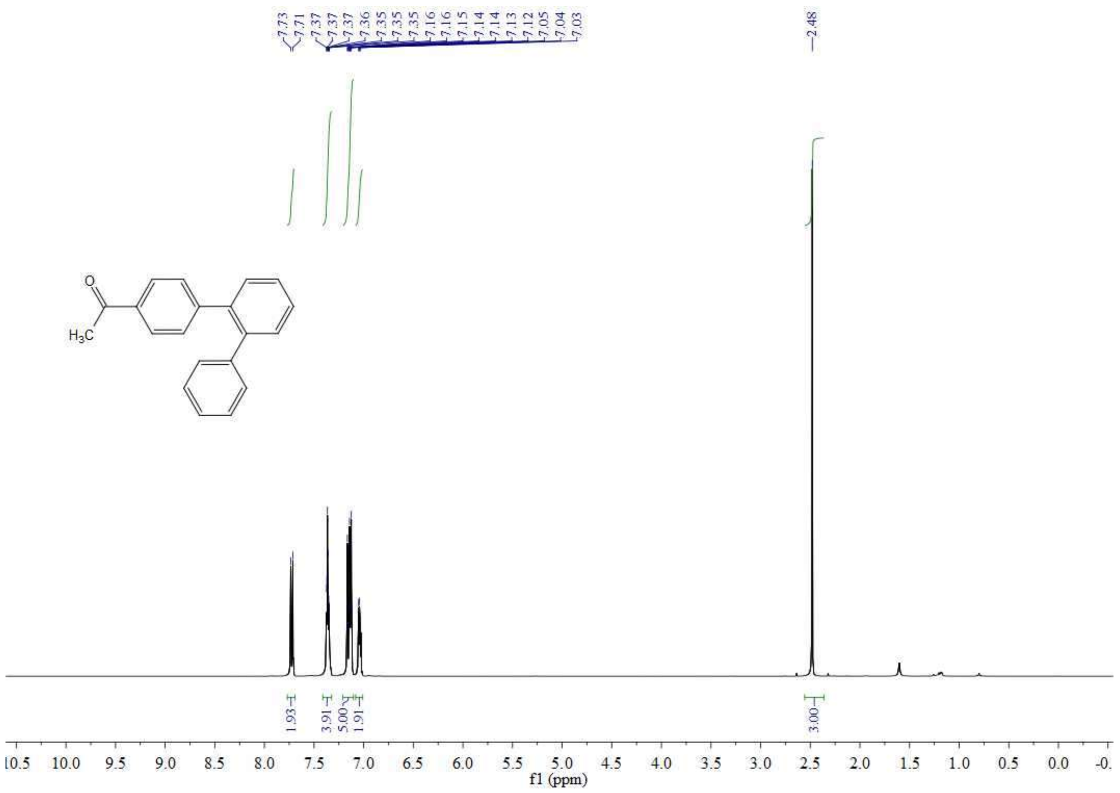

^{13}C NMR

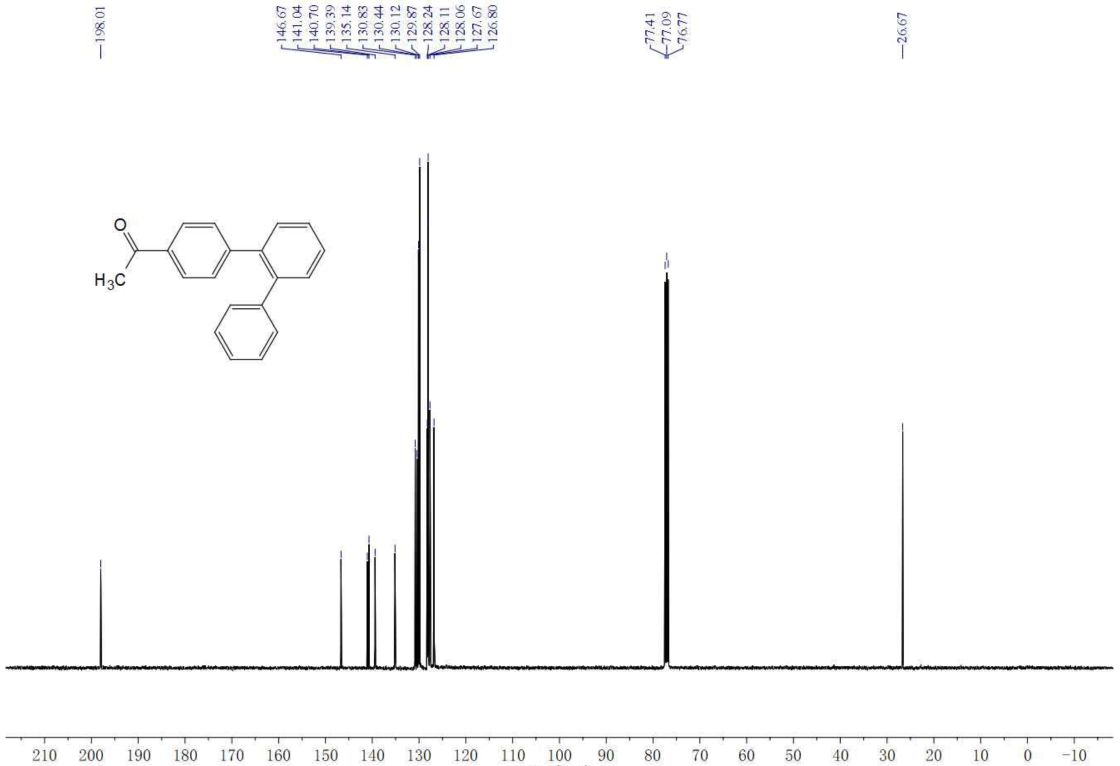

4-Acetyl-2'-formylbiphenyl 3ak

^{1}H NMR

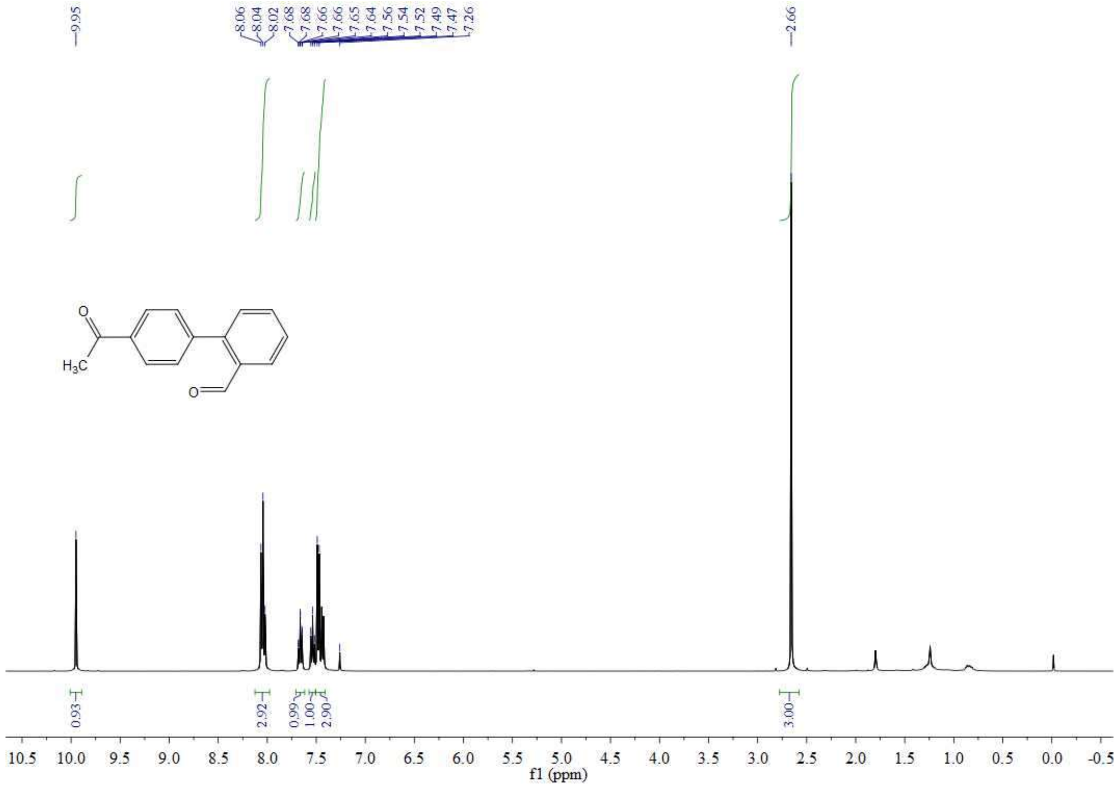

^{13}C NMR

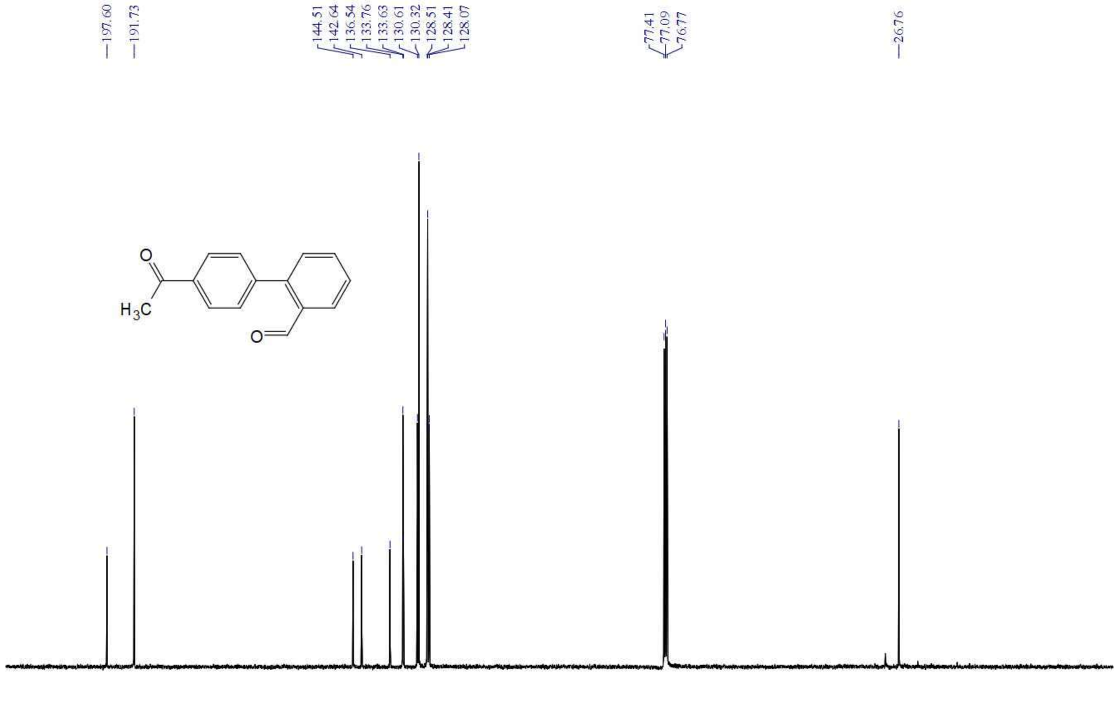

4-(2'-Thienyl)acetophenone 3al

^{1}H NMR

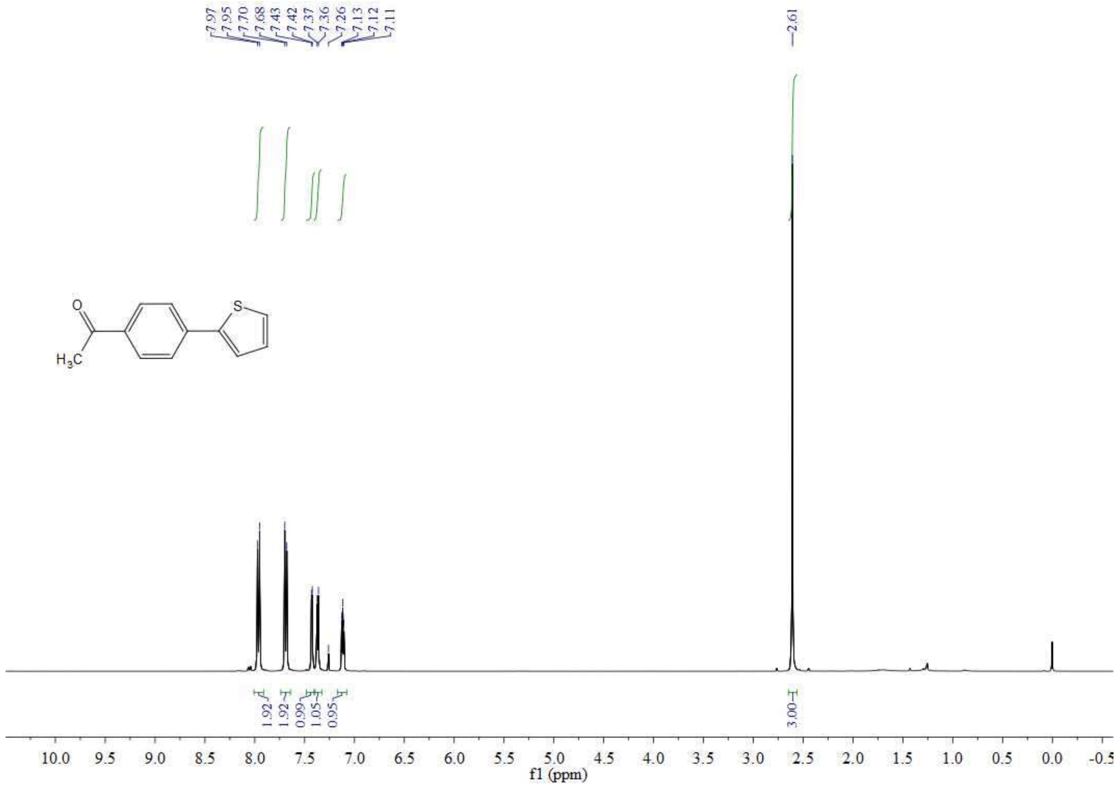

^{13}C NMR

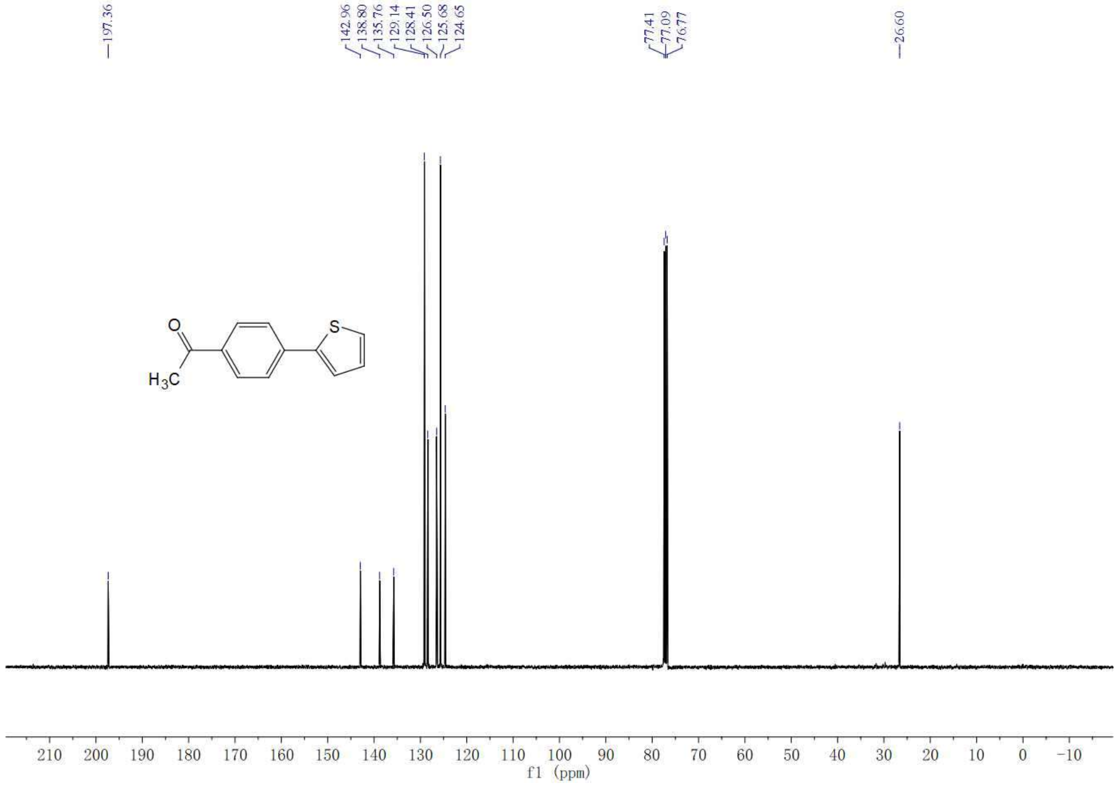

4-(3'-Furyl)acetophenone 3am

^{1}H NMR

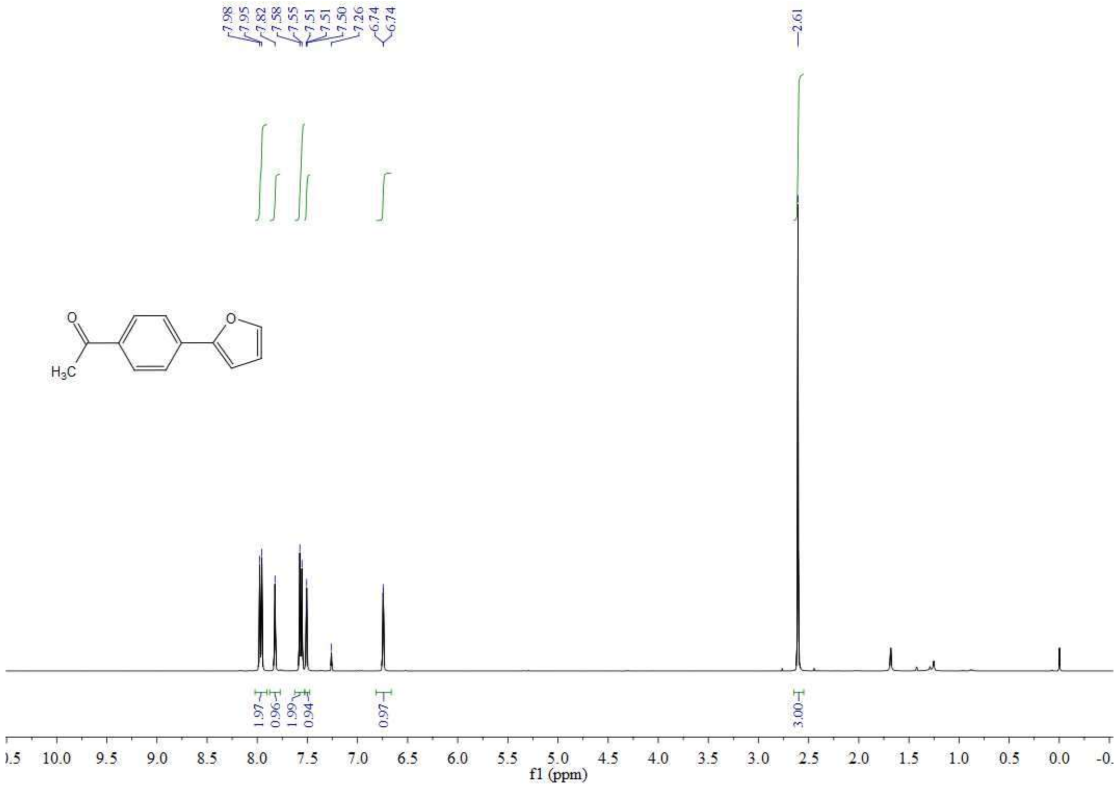

^{13}C NMR

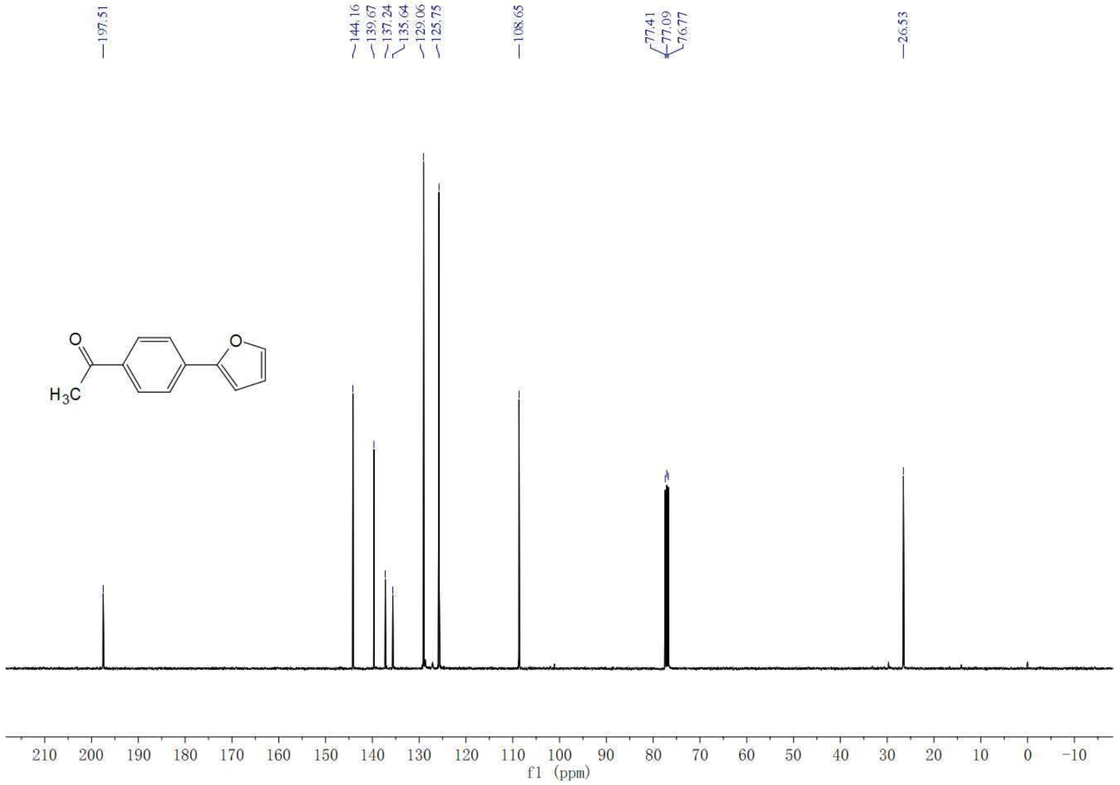

4-Tosyloxybiphenyl

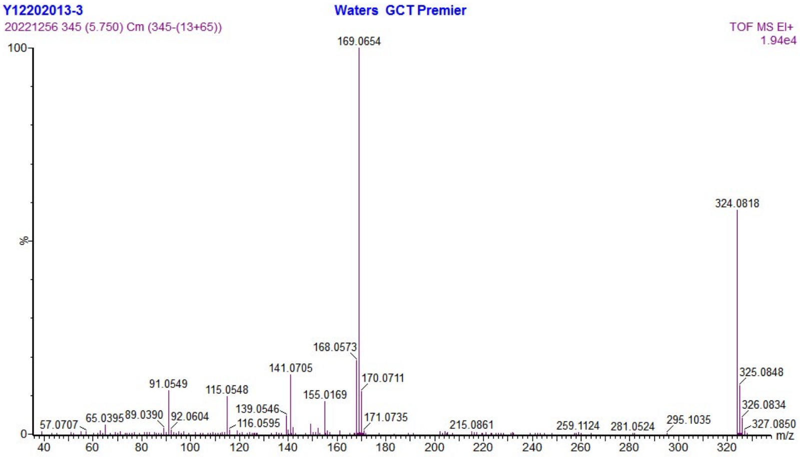

Supplement: Supplementary file 1 [file molecules-28-00636-s001.zip › molecules-2144002-supplementary.pdf]
